# Efficacy of mobilization with movement in chronic shoulder pain: a systematic review and meta-analysis of controlled trials

**DOI:** 10.1186/s12891-025-09422-0

**Published:** 2026-03-20

**Authors:** Arnstein Storås, Fabian Lillebostad, Sturla Haslerud, Jan Magnus Bjordal, Ernesto Cesar Pinto Leal-Junior, Mark I. Johnson, Martin Bjørn Stausholm

**Affiliations:** 1 Department of Global Public Health and Primary Care, Faculty of Medicine, University of Bergen, Bergen, Norway; 2 Department of Public Health, Faculty of Health Sciences, University of Stavanger, Stavanger, Norway; 3 Laboratory of Phototherapy and Innovative Technologies in Health (LaPIT), Postgraduate Program in Rehabilitation Sciences, Nove de Julho University (UNINOVE), São Paulo, Brazil; 4 Centre for Pain Research, School of Health, Leeds Beckett University, Leeds, UK; 5 The Research and Implementation Unit PROgrez, Department of Physiotherapy and Occupational Therapy, Central and West Zealand Hospital, Slagelse, Denmark; 6 Department of Occupational and Social Medicine, Copenhagen University Hospital Holbæk, Holbæk, Denmark

**Keywords:** Chronic shoulder pain, Controlled trials, Mobilization with movement, Mulligan, Systematic review

## Abstract

**Background:**

Shoulder pain is common and often slow to resolve despite treatment. We assessed the efficacy of Mobilization With Movement (MWM) in chronic shoulder pain.

**Methods:**

A systematic review was conducted. Five electronic databases, including MEDLINE, were searched from inception to August 2, 2024. Only controlled trials comparing MWM to conservative interventions, sham mobilization, or no-intervention control in adults aged 18 − 65 years with chronic shoulder pain (mean duration ≥ 3 months) were included. The outcomes were patient-reported pain (primary), patient-reported disability, quality of life, and range of motion (ROM). Random effects meta-analyses were performed. Risk of bias was assessed using the Physiotherapy Evidence Database scale.

**Results:**

Twenty-nine trials of adhesive capsulitis (AC) and six trials of subacromial pain syndrome (SAPS) were included. All active comparators were types of mobilization. AC analysis: MWM was significantly superior to other types of mobilization immediately post-therapy for improving pain (SMD = 1.03, 95% CI 0.59–1.46, *n* = 960), disability (SMD = 0.82, 95% CI 0.17–1.48, *n* = 361), external rotation ROM (SMD = 0.77, 95% CI 0.33–1.20, *n* = 992), and flexion ROM (SMD = 0.83, 95% CI 0.13–1.53, *n* = 727). Subgroup analyses demonstrated a significant superiority of MWM over Muscle Energy Techniques for reducing pain (SMD = 2.22, 95% CI 0.60–3.84, *n* = 271), and over Maitland mobilization for improving external rotation ROM (SMD = 0.74, 95% CI 0.38–1.09, *n* = 356). No significant differences were detected at 2–10-week follow-ups (*n* = 69–110). MWM was significantly superior to no-intervention control for pain, disability, and ROM immediately post-therapy. SAPS analysis: Although pain and ROM improvements were observed compared with no-intervention control, no significant differences were found versus sham mobilization. Quality of life was not assessed in any included trial. Risk of bias was moderate/high for AC trials and low/moderate for SAPS trials.

**Conclusions:**

In chronic AC, MWM resulted in significant improvements in pain, disability, and ROM immediately post-therapy. Follow-up data were too limited to draw firm conclusions. For chronic SAPS, there is insufficient evidence to support or refute an effect of MWM.

**PROSPERO registration:**

CRD42018109380.

**Supplementary Information:**

The online version contains supplementary material available at 10.1186/s12891-025-09422-0.

## Introduction

It has been estimated that shoulder pain affects between 18 and 26% of adults [1], and that approximately 67% of people will experience shoulder pain at some point in their lives [2]. The symptoms of shoulder disorders are often disabling and persistent, and a frequent reason for sick leave [1]. Only 50% of patients who begin a course of treatment for shoulder pain recover fully within 6 months [3]. Shoulder pain can arise from a variety of conditions, including adhesive capsulitis (AC), subacromial pain syndrome (SAPS), glenohumeral osteoarthritis, acromioclavicular joint disorder, and post-surgical causes, with AC and SAPS being the most prevalent [4–6].

AC, also known as “frozen shoulder”, is an inflammatory disorder characterized by fibrosis formation and restricted range of motion (ROM). It can manifest as a primary (idiopathic) condition, with onset occurring spontaneously or as a secondary condition following shoulder surgery or trauma [7]. The prevalence of AC in the adult general population is between 2 and 5%, while in individuals with diabetes, the prevalence may be as high as 13.4% [8]. Other risk factors for AC include being female and aged 40–60 years [7].

SAPS, also known as “shoulder impingement syndrome”, is characterized by pain in the acromial area, accompanied by weakness or a sensation of shoulder instability. Discomfort typically intensifies when lifting the arm [9]. Recent research suggests that SAPS is not associated with impingement of tissues, challenging previous conceptions of its mechanistic etiology [10]. SAPS is the most common shoulder disorder, accounting for approximately 36% of all shoulder diagnoses [4]. It encompasses a wide range of pathologies, including rotator cuff injuries and subacromial bursitis [11]. Risk factors for SAPS include being over the age of 50 years, diabetes, smoking, sleeping in the decubitus position, and participation in overhead sports [12–14]. There is ongoing debate in the medical community as to whether shoulder examination should focus on identifying a specific anatomical source of pain or adopt a non-specific (non-anatomical), impairment-based approach using treatment algorithms [15]. The latter emphasizes functional limitations, such as restricted ROM, weakness, or painful movements, rather than relying solely on special tests. Using this approach, SAPS can be categorized into Irritable SAPS, Non-irritable SAPS, and Degenerative SAPS subtypes [15]. Lewis et al. recommend exercise therapy for all SAPS categories, with the addition of cryotherapy for Irritable SAPS [15].

Manual therapy has also been suggested as a treatment option for reducing pain and disability in joint disorders. Variants of manual therapy include Kaltenborn mobilization [16], Maitland mobilization [17], Gong’s mobilization [18], Spencer mobilization [19], Muscle Energy Technique [20], and Brian Mulligan’s Mobilization With Movement (MWM) [16].

MWM stands out due to its patient-centered approach, emphasizing active participation alongside manual mobilization. In a recent textbook of manual therapy, Brian Mulligan and colleagues defined MWM as “The application of a sustained passive accessory force/glide to a joint while the patient actively performs a task that was previously identified as being problematic.” [21]. A key aspect of MWM involves identifying a task that the patient struggles to complete, often due to pain or joint stiffness. This task, known as the Client Specific Impairment Measure (CSIM), typically involves a movement or muscle contraction performed until the onset of pain, or to the end of the available ROM or maximum muscle contraction. The passive accessory force, which is usually a translatory or rotatory glide at the joint, must be applied close to the joint line to prevent undesirable movements. This force can be administered manually by the therapist or patient’s hands or using a treatment belt or sports tape applied to the skin. The direction of the accessory movement is determined by the one that produces the greatest improvement in the CSIM. Unlike the Maitland and Kaltenborn approaches, MWM does not involve grading the amount of force. Instead, when applying the MWM the practitioner should apply as much force in the mobilization as required to improve the CSIM without causing pain. Achieving pain-free overpressure at the symptomatic joint through MWM is thought to promote optimal recovery. If pain persists during MWM, therapists must adjust the technique [21].

Brian Mulligan initially favored a positional fault hypothesis in understanding musculoskeletal pain and MWM effects, yet this theory lacks verification [21]. Alternative hypotheses on MWM mechanisms have been proposed. It has been hypothesized that the effects of MWM are neurophysiological. Mechanoreceptors within the joint capsule are stimulated, subsequently modulating central nervous activity by inhibiting incoming nociceptive signals. This “gate control” mechanism suppresses pain. Furthermore, the stimulation of other centers, including the dorsal periaqueductal gray matter region, may induce a deep and selective analgesic effect [22]. A more recent theory proposes that pain reduction by MWM is related to habituation and excitation, suggesting that progressive exposure to feared movements that may develop due to fear-avoidance behavior can desensitize the nervous system through habituation [23].

Several systematic reviews with meta-analyses on the clinical efficacy of MWM on painful shoulders have been published since 2020; by Hassan et al. in 2021 [24], Satpute et al. in 2022 [25], Dias et al. in 2023 [26], and Çelik et al. in 2024 [27]. While the authors of the reviews generally agreed that MWM is effective, closer inspection reveals methodological limitations undermining confidence in their findings. For example, Hassan et al. included 11 trials, but only three contributed to the meta-analysis, and these compared MWM with heterogeneous controls (no-intervention and cryotherapy), undermining the validity of the pooled estimate [24]. Çelik et al. included 27 trials, of which only 12 were meta-analyzed, and they misinterpreted a statistically significant pain reduction by MWM as non-significant [27]. Both Dias et al. and Çelik et al. pooled data from patients with AC and SAPS without distinguishing between the two diagnoses [26, 27], and none of the four reviews accounted for whether participants were in the acute or chronic stage of pain [24–27]. In addition, Satpute et al. mixed distinct comparators in their meta-analyses, combining no-intervention with active controls in the AC analyses and sham, no-intervention, and cryotherapy in the SAPS analyses. Moreover, in the AC meta-analysis of pain by Satpute et al., the baseline value of a control group was mistakenly used instead of the post-intervention result [25, 28], overestimating the effectiveness of MWM.

Therefore, we decided to conduct a systematic review and meta-analysis accounting for limitations in previous reviews. Our hypothesis was that MWM can reduce pain and disability and increase quality of life (QoL) and shoulder ROM in adults with chronic shoulder pain, compared with other conservative interventions, sham mobilization, or no-intervention control.

## Methods

This systematic review was conducted in adherence to a peer-reviewed protocol article [29] based on a PROSPERO registration (CRD42018109380) and is reported in accordance with the Preferred Reporting Items of Systematic reviews and Meta-Analysis PRISMA 2020 statement [30].

### Study selection criteria and search strategy

We included any type of controlled trials involving adults aged between 18 and 65 years diagnosed with chronic shoulder pain (mean ≥ 3 months [31]), in which the efficacy of MWM was compared with that of other conservative interventions, sham mobilization, or no-intervention control on patient-reported pain, patient-reported disability, QoL, and/or shoulder ROM. Cointerventions were permitted as long as they were applied equally in all groups.

Five electronic databases, including MEDLINE (via PubMed), Embase (via Ovid), Physiotherapy Evidence Database (PEDro), Cumulative Index to Nursing and Allied Health Literature, and Cochrane Central Register of Controlled Trials, were searched from inception to August 2, 2024. The database search strings included simple highly sensitive synonyms and controlled vocabularies for the shoulder and manual therapy. The PubMed search string is provided in the online supplemental material. The search was continued by reading reference lists and Google Scholar citations of the eligible trials and relevant reviews, and by consulting experts in the field. Only studies reported in English or Nordic languages were considered for inclusion.

Two review teams (M.B.S. with either A.S. or F.L.) independently screened titles/abstracts of all records identified in the search and evaluated full-text records against the eligibility criteria. The screening process was handled using EndNote. Disagreements were resolved by discussion, with a third reviewer making the final decision when necessary. Authors of records that lacked sufficient information regarding eligibility criteria were contacted twice via email, ResearchGate, and/or LinkedIn for clarification.

### Risk of bias assessment

Two reviewers (M.B.S. and A.S.) independently assessed the included trials for risk of bias at the outcome-level using the 0–10-point PEDro scale [32]. The patients were considered the assessors when patient-reported outcomes were assessed, and a research staff member was considered the assessor in the ROM assessment [32]. Disagreements in risk of bias judgment were resolved by discussion between the two reviewers. PEDro scores of ≥ 7, 5–6, and ≤ 4 were interpreted as low, moderate, and high risk of bias, respectively [33]. We planned to assess the likelihood of publication bias with graphical funnel plots, contingent upon the availability of ≥ 10 trials per meta-analysis [34].

### Data extraction and meta-analysis

The following information was extracted from the included trials: number of participants allocated to MWM and control groups; type and duration of interventions; outcome assessment tools; time points of assessments; participant characteristics, including age, sex, body mass index, diabetes status, baseline shoulder pain intensity, duration of pain prior to enrollment; effect estimates; and adverse events. One reviewer extracted the data independently (M.B.S.), and another reviewer (F.L.) subsequently carefully checked all the work for accuracy. Data-extraction disagreements were resolved by discussion between the two reviewers. The standard deviations for meta-analysis were extracted or estimated from other variance data in the following order: (1) standard error, (2) 95% confidence interval, (3) P-value, (4) interquartile range, (5) median of correlations, (6) visually from graph, or (7) other methods [34].

The primary outcome was patient-reported pain intensity, and the secondary outcomes were patient-reported disability, QoL, and shoulder ROM. Active ROM was preferred over passive ROM, and pain-free ROM was preferred over painful ROM. Meta-analyses were conducted using standardized mean difference (SMD), allowing outcomes of the same nature reported on different measurement scales to be pooled. The SMDs were adjusted to Hedges’ *g* and interpreted as follows: SMDs of 0.2, ~ 0.5, and > 0.8 represent a small, moderate, and large difference, respectively [34]. The DerSimonian and Laird version of the random effects meta-analysis model was utilized. The impact of heterogeneity (inconsistency) on the meta-analyses was examined using I^2^ statistics. The I^2^ values were categorized as low (25%), moderate (50%), and high (75%) [35].

Changes from baseline to all reassessment time points after completion of MWM were analyzed, with separate analyses for immediate post-therapy and follow-up results, and stratification by shoulder diagnosis and comparator type. MWM was compared with a specific form of intervention when at least five trials were available, as single trials often have greater statistical power than random-effects meta-analyses with fewer trials [36]. Risk of bias sensitivity analyses were conducted by subgrouping trials by PEDro scores: ≤ 4 and ≥ 5. Additional sensitivity analyses were conducted by subgrouping trials according to diabetes status: present, allowed (not explicitly excluded), or absent. M.B.S. conducted the meta-analyses using the software program Review Manager version 5.4 under the supervision of M.I.J.

## Results

### Literature search

**Fig. 1:**
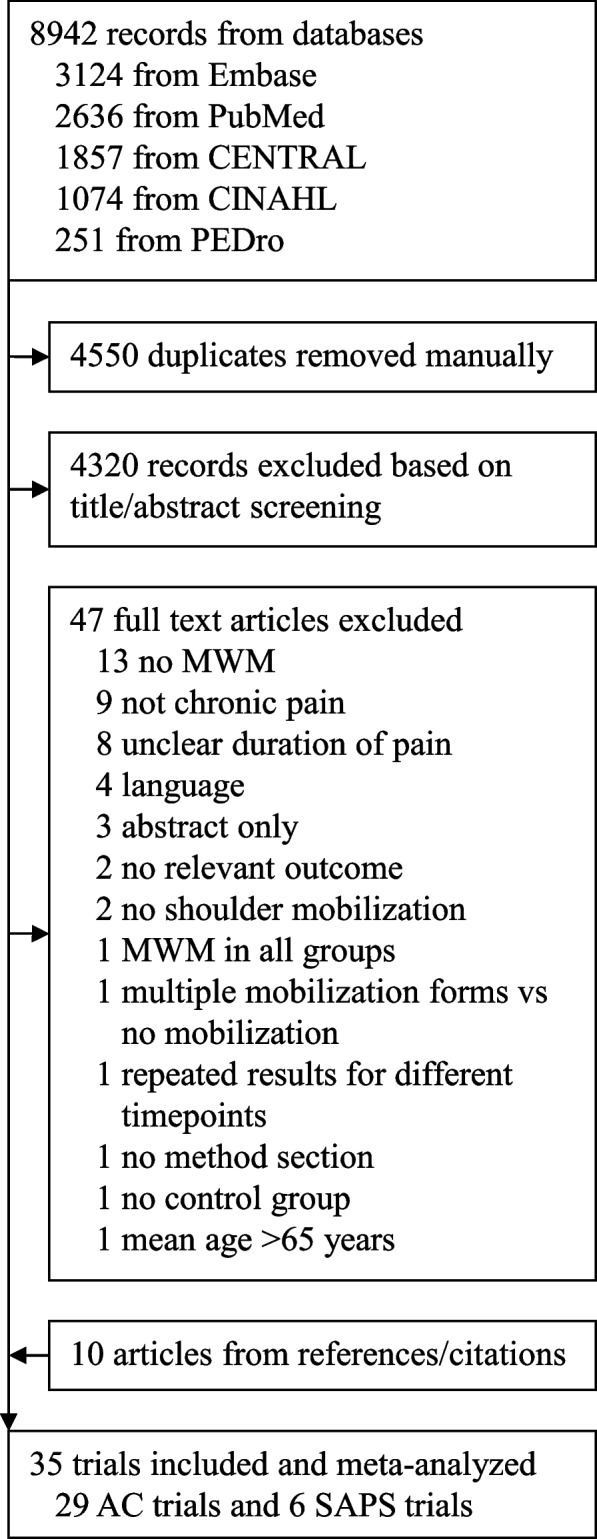
Flow chart illustrating the study selection process. AC = adhesive capsulitis; CINAHL = Cumulative Index to Nursing and Allied Health Literature; PEDro = Physiotherapy Evidence Database; SAPS = subacromial pain syndrome

The database search yielded 8,942 records, of which 72 were assessed in full-text format and 25 were ultimately included. An additional 10 eligible trials were identified through reference and citation searches, brining the total to 35 trials (29 of AC and 6 of SAPS). The trial identification process is illustrated in Fig. 1. Detailed reasons for full-text exclusions are provided in Supplemental Table S1.

### Characteristics of AC trials

The AC trials comprised 1,407 participants. There were 26 conservative comparisons, all of which were types of mobilization. MWM was compared with Maitland mobilization in eight trials [17, 37–43], Muscle Energy Technique in five trials [20, 44–47], Kaltenborn mobilization in three trials [16, 48, 49], stretching in three trials [50–52], Gong’s mobilization in two trials [18, 53], scapular mobilization in two trials [28, 54], Spencer mobilization in two trials [19, 55], and continuous passive motion in one trial [56]. MWM was compared with no-intervention control in six trials [38, 41, 55, 57–59]. None of the trials included a sham comparator. The median number of MWM sessions was 12.0 (range 5.0–24.0 sessions) (data from 25 trials), and the median number of treatment weeks was 3.0 (range 0.7–12.0 weeks) (data from 25 trials) (Table 1).

### Characteristics of SAPS trials

The SAPS trials comprised 246 participants. MWM was compared with conservative intervention (Kaltenborn mobilization) in one trial [60], sham MWM in two trials [61, 62], and no-intervention control in four trials [60, 63–65]. The median number of MWM sessions was 6.0 (range 4.0–21.0 sessions) (data from 5 trials), and the median number of treatment weeks was 4.0 (range 1.0–6.0 weeks) (data from 5 trials) (Table 1).

**Table 1:** Characteristics of the included trials

First author, year	MWM group at baseline	Control group at baseline	Interventions (differences in bold)	Outcome scales (time point of reassessment used for meta-analysis in bold)
Adhesive capsulitis				
Arshad 2015 [37]	n: 50 Women: - Age: ≥ 50, ≤ 70 yrs BMI: - Pain: - Pain duration: > 3 mo Diabetes: Allowed	n: 50 Women: - Age: ≥ 50, ≤ 70 yrs BMI: - Pain: - Pain duration: > 3 mo Diabetes: Allowed	16 sessions of **MWM** (glenohumeral glide), TENS, UT, and home-based ET versus **Maitland ERM**, TENS, UT, and home-based ET over 8 weeks	Pain: - Disability: - QoL: - ROM: External rotation and flexion Week of reassessment: **8**
Azam 2024 [43]	n: 19 Women: - Age: ≥ 35, ≤ 55 yrs BMI: - Pain: 6.37 ± 0.76 NPRS Pain duration: > 3 mo Diabetes: Allowed	n: 20 Women: - Age: ≥ 35, ≤ 55 yrs BMI: - Pain: 6.70 ± 0.47 NPRS Pain duration: > 3 mo Diabetes: Allowed	12 sessions of **MWM **(glenohumeral posterolateral, inferior, and lateral distraction glides), scapular mobilization, TENS, UT, and stretching versus 12 sessions of **Maitland mobilization**, scapular mobilization, TENS, UT, and stretching over 4 weeks	Pain: NPRS Disability: - QoL: - ROM: External rotation Week of reassessment: 2, **4**
Boruah 2015 [28]	n: 25 Women: 72.0% Age: 49.76 ± 3.49 yrs BMI: - Pain: 64.48 ± 6.46 SPADI Pain duration: 3–9 mo Diabetes: Allowed	n: 25 Women: 56.0% Age: 50.04 ± 3.34 yrs BMI: - Pain: 64.64 ± 5.76 SPADI Pain duration: 3–9 mo Diabetes: Allowed	15 sessions of **MWM** (glenohumeral posterolateral and inferior glides), HP and ET (pendular exercises) versus 15 sessions of **scapular mobilization**, HP, and ET over 3 weeks	Pain: SPADI Disability: - QoL: - ROM: Active external rotation and flexion Week of reassessment: **3**
Dilip 2016 [18]	n: 20 Women: 55.0% Age: 53.55 ± 7.57 yrs BMI: - Pain: 4.29 ± 1.01 cm VAS Pain duration: 4–12 mo Diabetes: Allowed	n: 20 Women: 55.0% Age: 51.00 ± 6.82 yrs BMI: - Pain: 4.45 ± 0.82 cm VAS Pain duration: 4–12 mo Diabetes: Allowed	10 sessions of **MWM** (glenohumeral posterolateral and inferior glide) and ET (Codman’s Pendulum exercise, Scapular stabilization, Active-assisted ROM and Finger walk) versus 10 sessions of **Gong’s mobilization** and ET over 2 weeks	Pain: VAS internal rotation Disability: - QoL: - ROM: - Week of reassessment: **2**
Doner 2013 [51]	n: 20 Women: 65.0% Age: 59.25 ± 9.17 yrs BMI: - Pain: 7.49 ± 1.57 cm VAS Pain duration: > 3 mo Diabetes: Allowed	n: 20 Women: 90.0% Age: 58.55 ± 8.57 yrs BMI: - Pain: 6.93 ± 1.39 cm VAS Pain duration: > 3 mo Diabetes: Allowed	15 sessions of **MWM** (glenohumeral glides), TENS, and HP versus 15 sessions of **stretching**, TENS, and HP over 3 weeks	Pain: VAS activity Disability: SDQ QoL: - ROM: Active external rotation and flexion Week of reassessment: **3**, **13**
Fernandes 2020 [48]	n: 28 Women: 32.1% Age: ≥ 40, ≤ 70 yrs BMI: - Pain: 59.00 ± 8.17 SPADI Pain duration: 3.29 ± 2.22 mo Diabetes: No	n: 28 Women: 50.0% Age: ≥ 40, ≤ 70 yrs BMI: - Pain: 66.5 ± 12.93 SPADI Pain duration: 3.73 ± 2.07 mo Diabetes: No	6 sessions of **MWM** (glenohumeral glide) versus 6 sessions of **Kaltenborn mobilization** over 2 weeks	Pain: SPADI pain Disability: SPADI disability QoL: - ROM: External rotation and flexion Week of reassessment: **2**
	n: 35 Women: - Age: ≥ 40, ≤ 60 yrs BMI: - Pain: - Pain duration: > 3 mo Diabetes: Allowed	n: 35 Women: - Age: ≥ 40, ≤ 60 yrs BMI: - Pain: - Pain duration: > 3 mo Diabetes: Allowed	**MWM** (glenohumeral glide), HP, and ET (AROM) versus **Maitland mobilization**, HP, and ET over 3 weeks*	Pain: VAS Disability: - QoL: - ROM: External rotation Week of reassessment: **3**
Jeyakumar 2018 [38]	n: 35 Women: - Age: ≥ 40, ≤ 60 yrs BMI: - Pain: - Pain duration: > 3 mo Diabetes: Allowed	n: 35 Women: - Age: ≥ 40, ≤ 60 yrs BMI: - Pain: - Pain duration: > 3 mo Diabetes: Allowed	**MWM** (glenohumeral glide), HP, and ET versus HP and ET over 3 weeks*	Pain: VAS Disability: - QoL: - ROM: External rotation Week of reassessment: **3**
Jung 2020 [56]	n: 15 Women: - Age: 53.93 ± 6.79 BMI: - Pain: 6.99 ± 0.70 cm VAS Pain duration: > 3 mo Diabetes: Allowed	n: 15 Women: - Age: 56.80 ± 8.24 BMI: - Pain: 7.46 ± 0.50 cm VAS Pain duration: > 3 mo Diabetes: Allowed	12 sessions of **MWM** (glenohumeral posterolateral and inferior glides) versus 12 sessions of **continuous passive motion** over 3 weeks	Pain: VAS Disability: - QoL: - ROM: Flexion Week of reassessment: **3**
Khalil 2022 [47]	n: 39 Women: 71.8% Age: 42.63 ± 11.80 yrs BMI: - Pain: 8.80 ± 0.60 cm VAS Pain duration: > 3 mo Diabetes: No	n: 39 Women: 48.7% Age: 44.90 ± 11.41 yrs BMI: - Pain: 8.74 ± 0.63 cm VAS Pain duration: > 3 mo Diabetes: No	6 weeks of **MWM** (glenohumeral glide) versus 6 weeks of **MET**	Pain: VAS Disability: SPADI disability QoL: - ROM: External rotation and flexion Week of reassessment: 3,** 6**
Khan 2023 [50]	n: 22 Women: 68.2% Age: 49.22 ± 6.67 yrs BMI: - Pain: 7.73 ± 0.99 NPRS Pain duration: > 3 mo Diabetes: Allowed	n: 22 Women: 40.9% Age: 54.13 ± 6.33 yrs BMI: - Pain: 6.77 ± 1.34 NPRS Pain duration: > 3 mo Diabetes: Allowed	16 sessions of **MWM** and conventional physiotherapy (HP, interferential therapy, and AROM exercises) versus 16 sessions of **Sleeper stretch** over 4 weeks	Pain: NPRS Disability: - QoL: - ROM: External rotation and flexion Week of reassessment: 2, **4**
Khyathi 2015 [19]	n: 20 Women: 40% Age: 50.40 ± 5.42 yrs BMI: - Pain: 6.71 ± 1.25 cm VAS Pain duration: > 3 mo Diabetes: No	n: 20 Women: 45% Age: 50.85 ± 5.14 yrs BMI: - Pain: 6.30 ± 1.11 cm VAS Pain duration: > 3 mo Diabetes: No	5 sessions of **MWM** (glenohumeral posterolateral and inferior glides) and supervised ET (pendulum exercise, isometric scapular retraction, rotator cuff exercises, scapular stabilization, finger walk on a wall, active-assisted ROM exercises) versus 5 sessions of **Spencer mobilization** and supervised ET over 5 days	Pain: VAS Disability: SPADI disability QoL: - ROM: Active external rotation Day of reassessment: **5**
Mehta 2018 [52]	n: 30 Women: - Age: 53.90 ± 6.53 yrs BMI: - Pain: 64.7 SPADI Pain duration: > 3 mo Diabetes: Allowed	n: 30 Women: - Age: 54.76 ± 6.96 yrs BMI: - Pain: 75.9 SPADI Pain duration: > 3 mo Diabetes: Allowed	6 sessions of **MWM** (glenohumeral posterolateral, lateral, and distractive glides), UT, and ET (scapular setting) versus 6 sessions of **passive stretching exercises**, UT, and ET over 6 days	Pain: SPADI pain Disability: SPADI disability QoL: - ROM: Active external rotation and flexion Day of reassessment: **6**
Minerva 2016 [40]	n: 30 Women: - Age: ≥ 40, ≤ 60 yrs BMI: - Pain: - Pain duration: > 3 mo Diabetes: Allowed	n: 30 Women: - Age: ≥ 40, ≤ 60 yrs BMI: - Pain: - Pain duration: > 3 mo Diabetes: Allowed	8 sessions of **MWM** (glenohumeral posterolateral glide) and supervised ET versus 8 sessions of **Maitland mobilization** and supervised ET over 4 weeks	Pain: - Disability: - QoL: - ROM: External rotation Week of reassessment: **4**
Nithya 2021 [20]	n: 15 Women: 53.3% Age: 54.67 ± 3.81 yrs BMI: - Pain: 6.20 ± 0.77 NPRS Pain duration: 4 mo Diabetes: No	n: 15 Women: 60.0% Age: 52.73 ± 4.83 yrs BMI: - Pain: 6.47 ± 0.52 NPRS Pain duration: 4 mo Diabetes: No	12 sessions of **MWM** (glenohumeral posterolateral glide) and 16 sessions of HP, stretching, and active ERM versus 12 sessions of **MET** (post isometric relaxation) and 16 sessions of HP, stretching, and active ERM over 4 weeks	Pain: NPRS Disability: - QoL: - ROM: External rotation Week of reassessment: **4**
Pankaj 2013 [53]	n: 15 Women: 46.7% Age: 44.9 ± 3.0 yrs BMI: - Pain: 7.64 ± 1.49 cm VAS Pain duration: > 3 mo Diabetes: No	n: 15 Women: 53.3% Age: 43.9 ± 3.5 yrs BMI: - Pain: 7.23 ± 1.46 cm VAS Pain duration: > 3 mo Diabetes: No	15 sessions of **MWM** (glenohumeral posterolateral glide) and ET (pendular exercises, isometric scapular retraction, rotator cuff strengthening, horizontal abduction and scapular stabilization) versus 15 sessions of **Gong’s mobilization** and ET over 3 weeks	Pain: VAS active abduction Disability: - QoL: - ROM: - Week of reassessment: **3**
Patel 2022 [44]	n: 21 Women: - Age: 54.38 yrs BMI: - Pain: 4.95 ± 0.86 NPRS Pain duration: > 3 mo Diabetes: No	n: 21 Women: - Age: 52.52 yrs BMI: - Pain: 3.05 ± 1.28 NPRS Pain duration: > 3 mo Diabetes: No	20 sessions of **MWM** (glenohumeral posterior, posterolateral, and inferior glides) and conventional physiotherapy (HP and Codman’s, finger walk, and wand exercises) versus 20 sessions of **MET** and conventional physiotherapy over 4 weeks	Pain: NPRS Disability: - QoL: - ROM: External rotation and flexion Week of reassessment: **4**
Patil 2010 [49]	n: 30 Women: 53.3% Age: 55.2 ± 8.78 yrs BMI: - Pain: 6.7 ± 1.0 cm VAS Pain duration: > 3 mo Diabetes: Allowed	n: 30 Women: 56.7% Age: 54.5 ± 8.6 yrs BMI: - Pain: 7.0 ± 1.1 cm VAS Pain duration: > 3 mo Diabetes: Allowed	15 sessions of **MWM** (glenohumeral glide), short wave diathermy (heat), supervised ET (pendulum and shoulder wheel exercises) versus 15 sessions of **Kaltenborn mobilization**, short wave diathermy, and supervised ET over 3 weeks	Pain: VAS Disability: - QoL: - ROM: Active external rotation and flexion Week of reassessment: **3**
Ranjana 2016 [41]	n: 15 Women: - Age: ≥ 40, ≤ 60 yrs BMI: - Pain: 6.42 ± 0.74 cm VAS Pain duration: > 3 mo Diabetes: No	n: 15 Women: - Age: ≥ 40, ≤ 60 yrs BMI: - Pain: 6.66 ± 0.35 cm VAS Pain duration: > 3 mo Diabetes: No	12 sessions of **MWM** (glenohumeral posterolateral glide), HP, and home and supervised ET (Codman exercise, shoulder wheel exercise, wall ladder exercise, and self-stretching of shoulder capsules) versus 12 sessions of **Maitland mobilization**, HP, and home and supervised ET over 4 weeks	Pain: VAS activity Disability: - QoL: - ROM: Passive external rotation Week of reassessment: **4**, **6**
Ranjana 2016 [41]	n: 15 Women: - Age: ≥ 40, ≤ 60 yrs BMI: - Pain: 6.42 ± 0.74 cm VAS Pain duration: > 3 mo Diabetes: No	n: 15 Women: - Age: ≥ 40, ≤ 60 yrs BMI: - Pain: 6.20 ± 0.66 cm VAS Pain duration: > 3 mo Diabetes: No	12 sessions of **MWM** (glenohumeral posterolateral glide), HP, and home and supervised ET versus 12 sessions HP and home and supervised ET over 4 weeks	Pain: VAS activity Disability: - QoL: - ROM: Passive external rotation Week of reassessment: **4**, **6**
Rathod 2019 [16]	n: 20 Women: - Age: - BMI: - Pain: 5.45 ± 0.89 cm VAS Pain duration: 3–9 mo Diabetes: Allowed	n: 20 Women: - Age: - BMI: - Pain: 5.30 ± 0.86 cm VAS Pain duration: 3–9 mo Diabetes: Allowed	10 sessions of **MWM** (glenohumeral posterolateral glide) versus 10 sessions of **Kaltenborn mobilization** over 3 weeks	Pain: VAS Disability: - QoL: - ROM: External rotation and flexion Week of reassessment: **3**
Rayudu 2018 [46]	n: 30 Women: - Age: < 65 yrs BMI: - Pain: 34.13 ± 4.01 SPADI Pain duration: > 3 mo Diabetes: Allowed	n: 30 Women: - Age: < 65 yrs BMI: - Pain: 33.73 ± 2.63 SPADI Pain duration: > 3 mo Diabetes: Allowed	9 sessions of **MWM** (glenohumeral posterolateral glide) and ET versus 9 sessions of **MET** and ET over 3 weeks	Pain: SPADI pain Disability: SPADI disability QoL: - ROM: External rotation Week of reassessment: **3**
Razzaq 2022 [45]	n: 32 Women: - Age: 49.93 ± 6.69 yrs BMI: - Pain: 6.56 ± 0.56 NPRS Pain duration: 4–12 mo Diabetes: Allowed	n: 32 Women: - Age: 49.17 ± 8.92 yrs BMI: - Pain: 6.41 ± 0.50 NPRS Pain duration: 4–12 mo Diabetes: Allowed	9 sessions of **MWM**, HP, and ET (pulley rope, wall climbing, shoulder wheel) versus 9 sessions of **MET**, HP, and ET over 3 weeks	Pain: NPRS Disability: - QoL: - ROM: Flexion Week of reassessment: **3**
Reddy 2015 [57]	n: 15 Women: 47.0% Age: 54.2 ± 6.52 yrs BMI: 25.98 ± 3.35 Pain: 7.16 ± 0.86 cm VAS Pain duration: 4.73 ± 2.22 mo Diabetes: Allowed	n: 15 Women: 33.0% Age: 52.7 ± 5.42 yrs BMI: 25.87 ± 3.60 Pain: 7.02 ± 1.08 cm VAS Pain duration: 4.80 ± 2.51 mo Diabetes: Allowed	15 sessions of **MWM** (glenohumeral glide), HP, TENS, and ET (shoulder mobility) versus 15 sessions of HP, TENS, and ET over 15 days	Pain: VAS Disability: DASH QoL: - ROM: Active external rotation and flexion Day of reassessment: **15**
Sai 2015 [58]	n: 34 Women: 58.8% Age: 50.5 ± 5.9 yrs BMI: - Pain: 6.63 ± 1.28 cm VAS Pain duration: 7.2 ± 2.9 mo Diabetes: Yes	n: 34 Women: 55.9% Age: 51.0 ± 7.7 yrs BMI: - Pain: 6.25 ± 0.95 cm VAS Pain duration: 6.7 ± 2.5 mo Diabetes: Yes	24 sessions **MWM** (glenohumeral posterolateral glide) and supervised ET (Codman’s exercises, stretching, active assisted range of motion exercises, scapular setting) and 168 sessions of home-based ET versus 24 sessions of supervised ET and 168 sessions of home-based ET over 12 weeks	Pain: VAS Disability: DASH QoL: - ROM: Active external rotation and flexion Week of reassessment: 3, 6, **12**
Shera 2023 [55]	n: 12 Women: 58.3% Age: ≥ 30, ≤ 55 yrs BMI: - Pain: 8.50 ± 1.16 NPRS Pain duration: > 3 mo Diabetes: Yes	n: 12 Women: 41.7% Age: ≥ 30, ≤ 55 yrs BMI: - Pain: 7.91 ± 1.14 NPRS Pain duration: > 3 mo Diabetes: Yes	16 sessions of **MWM** (glenohumeral glide) and 60 sessions of HP and home exercises (Codman’s, wand, pulley, finger ladder, isometrics, active and active aided ROM, and pectoral stretch) versus 16 sessions of **Spencer mobilization** and 60 sessions of HP and home exercises over 4 weeks	Pain: NPRS Disability: - QoL: - ROM: - Week of reassessment: **4**
Shera 2023 [55]	n: 12 Women: 58.3% Age: ≥ 30, ≤ 55 yrs BMI: - Pain: 8.50 ± 1.16 NPRS Pain duration: > 3 mo Diabetes: Yes	n: 12 Women: 58.3% Age: ≥ 30, ≤ 55 yrs BMI: - Pain: 8.16 ± 1.14 NPRS Pain duration: > 3 mo Diabetes: Yes	16 sessions of **MWM** (glenohumeral glide) and 60 sessions of HP and home exercises (Codman’s, wand, pulley, finger ladder, isometrics, active and active aided ROM, and pectoral stretch) versus 60 sessions of HP and home exercises over 4 weeks	Pain: NPRS Disability: - QoL: - ROM: - Week of reassessment: **4**
Shrivastava 2011 [42]	n: 20 Women: 40% Age: 51.15 ± 8.53 yrs BMI: - Pain: 5.85 cm VAS Pain duration: > 3 mo Diabetes: Allowed	n: 20 Women: 60% Age: 59.20 ± 7.18 yrs BMI: - Pain: 7.35 cm VAS Pain duration: > 3 mo Diabetes: Allowed	12 sessions of **MWM** (glenohumeral posterolateral glide) over 2 weeks followed by 2 weeks of home exercises versus 12 sessions of **Maitland mobilization** over 2 weeks followed by 2 weeks of exercises	Pain: VAS Disability: - QoL: - ROM: External rotation and flexion Week of reassessment: **4**
Yang 2007 [17]	n: 14 Women: 78.6% Age: 58.0 ± 10.1 yrs BMI: - Pain: - Pain duration: 5.5 ± 2.5 mo Diabetes: No	n: 14 Women: 92.9% Age: 53.3 ± 6.5 yrs BMI: - Pain: - Pain duration: 4.5 ± 2.0 mo Diabetes: No	3 weeks of Maitland/Kaltenborn MRM, followed by 3 weeks of **MWM** (glenohumeral glide) and then 3 weeks of Maitland/Kaltenborn MRM biweekly versus 3 weeks of Maitland/Kaltenborn MRM, followed by 3 weeks of **Maitland ERM** and then 3 weeks of Maitland/Kaltenborn MRM biweekly	Pain: - Disability: FLEX-SF QoL: - ROM: External rotation# Week of reassessment: 3, **6**, **9**
Yeole 2017 [59]	n: 15 Women: - Age: ≥ 40, ≤ 70 yrs BMI: - Pain: 7.93 ± 0.88 NPRS Pain duration: 3–4 mo Diabetes: Allowed	n: 15 Women: - Age: ≥ 40, ≤ 70 yrs BMI: - Pain: 8.06 ± 1.09 NPRS Pain duration: 3–4 mo Diabetes: Allowed	1 week of **MWM** (glenohumeral posterolateral and inferior glides) and supervised ET versus 1 week of supervised ET	Pain: NPRS Disability: SPADI disability QoL: - ROM: External rotation and flexion Week of reassessment: **1**
Youssef 2015 [39]	n: 15 Women: - Age: 54.8 ± 5.85 yrs BMI: - Pain: - Pain duration: > 3 mo Diabetes: Yes	n: 15 Women: - Age: 53.4 ± 5.23 yrs BMI: - Pain: - Pain duration: > 3 mo Diabetes: Yes	18 sessions of **MWM** (glenohumeral posterolateral and inferior glides) versus 18 sessions of **Maitland ERM** over 6 weeks	Pain: - Disability: - QoL: - ROM: Active external rotation and flexion Week of reassessment: **6**
Zaghloul 2022 [54]	n: 18 Women: 44.4% Age: 42.89 ± 4.28 yrs BMI: - Pain: 7.57 ± 1.37 cm VAS Pain duration: > 3 mo Diabetes: Allowed	n: 18 Women: 38.9% Age: 45.68 ± 4.35 yrs BMI: - Pain: 7.78 ± 1.29 cm VAS Pain duration: > 3 mo Diabetes: Allowed	18 sessions of **MWM** (glenohumeral posterolateral and inferior glides) and HP versus 18 sessions of **scapular mobilization** and HP over 6 weeks	Pain: VAS Disability: - QoL: - ROM: Active external rotation and flexion Week of reassessment: **6**
Subacromial pain syndrome				
Dalvi 2024 [64]	n: 40 Women: - Age: ≥ 30, ≤ 60 yrs BMI: - Pain: 6.30 ± 0.48 cm VAS Pain duration: > 3 mo Diabetes: Allowed	n: 40 Women: - Age: ≥ 30, ≤ 60 yrs BMI: - Pain: 6.09 ± 0.58 cm VAS Pain duration: > 3 mo Diabetes: Allowed	20 sessions of **MWM** (glenohumeral glide) and ET and 5 sessions of UT and cryotherapy versus 20 sessions of ET and 5 sessions of UT and cryotherapy over 4 weeks	Pain: VAS activity Disability: - QoL: - ROM: External rotation and flexion Week of reassessment: **4**
Delgado-Gil 2015 [61]	n: 21 Women: 81.0% Age: 55.4 ± 7.8 yrs BMI: - Pain: 6.5 ± 1.6 NPRS Pain duration: 9.2 ± 6.7 mo Diabetes: No	n: 21 Women: 81.0% Age: 54.3 ± 10.0 yrs BMI: - Pain: 6.6 ± 2.1 NPRS Pain duration: 11.7 ± 7.9 mo Diabetes: No	4 sessions of **MWM** (glenohumeral posterolateral glide) versus 4 sessions of **sham MWM** over 2 weeks	Pain: NPRS Disability: - QoL: - ROM: Maximum active external rotation and pain-free active flexion Week of reassessment: **2**
Guimarães 2016 [62]	n: 14 Women: 57.0% Age: 30.3 ± 6.9 yrs BMI: 24.7 ± 2.9 Pain: 41.1 ± 28.7 SPADI pain Pain duration: 31.9 ± 21.6 mo Diabetes: No	n: 13 Women: 54.0% Age: 31.9 ± 9.2 yrs BMI: 23.5 ± 3.2 Pain: 43.8 ± 25.0 SPADI pain Pain duration: 31.0 ± 37.9 mo Diabetes: No	4 sessions of **MWM** (glenohumeral posterolateral glide) versus 4 sessions of **sham MWM** over 1 week	Pain: SPADI pain Disability: SPADI disability QoL: - ROM: Pain-free active external rotation and flexion Week of reassessment: **1**
Ishaq 2023 [65]	n: 20 Women: 50.0% Age: 49.12 ± 9.12 yrs BMI: - Pain: 7.8 NPRS Pain duration: 6.6 ± 5.45 mo Diabetes: Allowed	n: 20 Women: 65.0% Age: 49.76 ± 7.58 yrs BMI: - Pain: 7.3 NPRS Pain duration: 6.8 ± 6.72 mo Diabetes: Allowed	21 sessions of **MWM** (glenohumeral posterolateral glide) and routine physiotherapy (thermotherapy, multiple angle isometrics, therapeutic band exercises, and soft tissue mobilization) versus 21 sessions of **MET** and routine physiotherapy over 6 weeks	Pain: NPRS Disability: - QoL: - ROM: External rotation and flexion Week of reassessment: **6**
Kachingwe 2008 [60]	n: 9 Women: 44.4% Age: 48.9 ± 13.7 yrs BMI: - Pain: 5.2 ± 2.5 cm VAS Pain duration: 22.6 ± 17.4 mo Diabetes: No	n: 9 Women: 55.6% Age: 43.4 ± 14.7 yrs BMI: - Pain: 6.3 ± 1.6 cm VAS Pain duration: 19.2 ± 24.6 mo Diabetes: No	6 sessions of **MWM** (glenohumeral posterior glide) and CP and 48 sessions of ET (posterior capsule stretching, postural correction, scapular stabilization, rotator cuff strengthening) versus 6 sessions of **Kaltenborn mobilization** and CP and 48 sessions of ET over 6 weeks	Pain: VAS maximum pain Disability: - QoL: - ROM: Pain-free active flexion Week of reassessment: **6**
Kachingwe 2008 [60]	n: 9 Women: 44.4% Age: 48.9 ± 13.7 yrs BMI: - Pain: 5.2 ± 2.5 cm VAS Pain duration: 22.6 ± 17.4 mo Diabetes: No	n: 8 Women: 50.0% Age: 47.3 ± 20.1 yrs BMI: - Pain: 5.7 ± 3.0 cm VAS Pain duration: 32.5 ± 60.2 mo Diabetes: No	6 sessions of **MWM** (glenohumeral posterior glide) and CP and 48 sessions of ET versus 6 sessions of CP and 48 sessions of ET over 6 weeks	Pain: VAS maximum pain Disability: - QoL: - ROM: Pain-free active flexion Week of reassessment: **6**
Neelapala 2016 [63]	n: 15 Women: 26.7% Age: 40.23 ± 10.55 yrs BMI: - Pain: 6.69 ± 1.05 cm VAS Pain duration: 5.08 ± 3.45 mo Diabetes: Allowed	n: 16 Women: 25.0% Age: 42.41 ± 10.38 yrs BMI: - Pain: 5.96 ± 1.13 cm VAS Pain duration: 6.00 ± 4.04 mo Diabetes: Allowed	3 sessions of **MWM** (glenohumeral posterolateral glide) and ET (pain-free AROM) versus 3 sessions of ET over an unknown time-period	Pain: VAS pain on movement Disability: - QoL: - ROM: - Week of reassessment: Immediately after completed therapy

*AROM* Active Range of Motion, *BMI* Body Mass Index, *CP* Cold Packs, *ERM* End-Range Mobilization, *ET* exercise therapy, *FLEX-SF* Flexilevel Scale of Shoulder Function, *GM* Glenohumeral Mobilization, *HP* Hot Packs, *M* mobilization, *MRM* Mid-Range Mobilization, *MWM* Mobilization With Movement, *MET* Muscle Energy Technique, *NPRS* Numeric Pain Rating Scale, *QoL* Quality of Life, *ROM* Range Of Motion, *SDQ* Shoulder Disability Questionnaire, *SPADI* Shoulder Pain and Disability Index, *TENS* Transcutaneous Electrical Nerve Stimulation, *UT* Ultrasound Therapy, *VAS* Visual Analogue Scale

^*^The authors stated that the intervention lasted both 3 and 4 weeks – we assumed the intervention lasted 3 weeks

^#^ROM not reported at week 9 due to dropouts

### Risk of bias within studies

A mean PEDro score is presented alongside each meta-analysis effect estimate. Risk of bias results for individual trials are shown in Supplemental Tables S2–S3, with corresponding reasons described immediately below. For AC trials, lower scores were mainly due to insufficient reporting of allocation concealment and intention-to-treat analysis, as well as lack of blinding of subjects, therapists, and assessors of pain, disability, and ROM. For SAPS trials, lower scores were primarily attributed to insufficient reporting of allocation concealment and lack of blinding of subjects, therapists, and assessors of pain and disability.

### Risk of publication bias

Minor asymmetries in the funnel plots with ≥ 10 trials were observed for MWM versus other types of mobilization for pain, external rotation ROM, and flexion ROM in AC (Supplemental Figures S1–S3).

### AC pain results

**Fig. 2:**
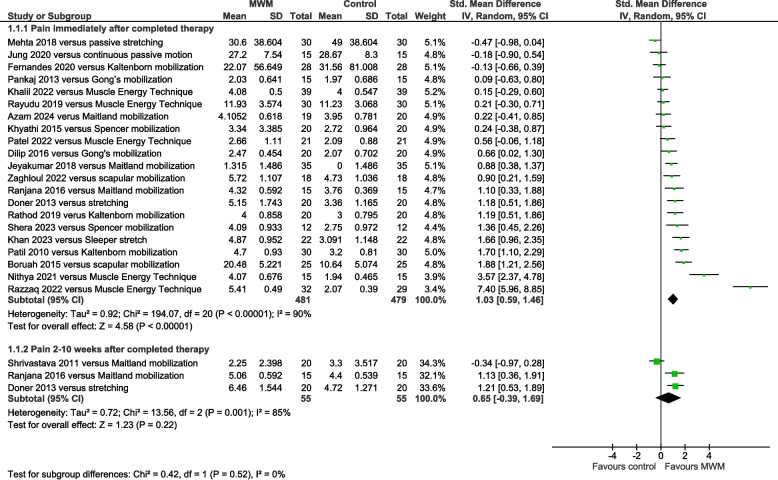
Forest plot of AC pain results immediately post-therapy and 2–10 weeks later – MWM versus other types of mobilization. AC = adhesive capsulitis; MWM = Mobilization With Movement

**Fig. 3:**
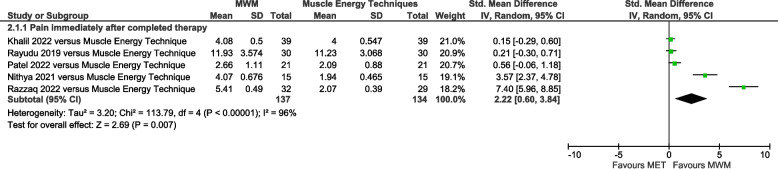
Forest plot of AC pain results immediately post-therapy – MWM versus Muscle Energy Techniques. AC = adhesive capsulitis; MWM = Mobilization With Movement

Pain was significantly reduced by MWM compared with other types of mobilization immediately post-therapy (SMD = 1.03, 95% CI 0.59 to 1.46, I^2^ = 90%, *n* = 960, PEDro score = 4.8), but not at the 2–10 weeks follow-up (SMD = 0.65, 95% CI −0.39 to 1.69, I^2^ = 85%, *n* = 110, PEDro score = 4.7) (Fig. 2). Compared with Muscle Energy Techniques, pain was significantly reduced by MWM immediately post-therapy (SMD = 2.22, 95% CI 0.60 to 3.84, I^2^ = 96%, *n* = 271, PEDro score = 4.6) (Fig. 3). Compared with no-intervention control, pain was significantly reduced by MWM immediately post-therapy (SMD = 1.49, 95% CI 0.75 to 2.23, I^2^ = 82%, *n* = 234, PEDro score = 5.2) (Supplemental Figure S4). A single trial showed that this was also evident 2 weeks later [41].

### AC disability results

**Fig. 4:**
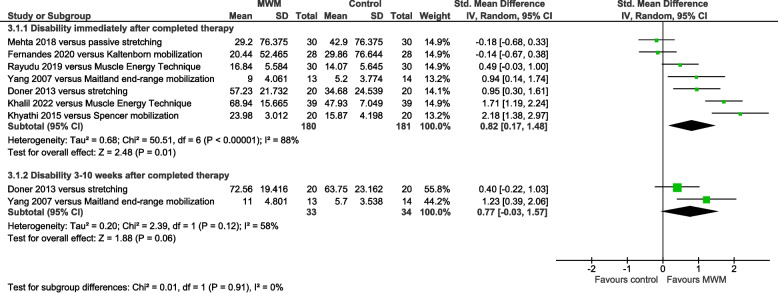
Forest plot of AC disability results immediately post-therapy and 3–10 weeks later – MWM versus other types of mobilization. AC = adhesive capsulitis; MWM = Mobilization With Movement

Disability was significantly reduced by MWM compared with other types of mobilization immediately post-therapy (SMD = 0.82, 95% CI 0.17 to 1.48, I^2^ = 88%, *n* = 361, PEDro score = 5.1), but not 3–10 weeks later (SMD = 0.77, 95% CI −0.03 to 1.57, I^2^ = 58%, *n* = 67, PEDro score = 6.0) (Fig. 4). Compared with no-intervention control, disability was significantly reduced by MWM immediately post-therapy (SMD = 0.58, 95% CI 0.22 to 0.93, I^2^ = 0%, *n* = 128, PEDro score = 5.0) (Supplemental Figure S5).

### AC external rotation ROM results

**Fig. 5:**
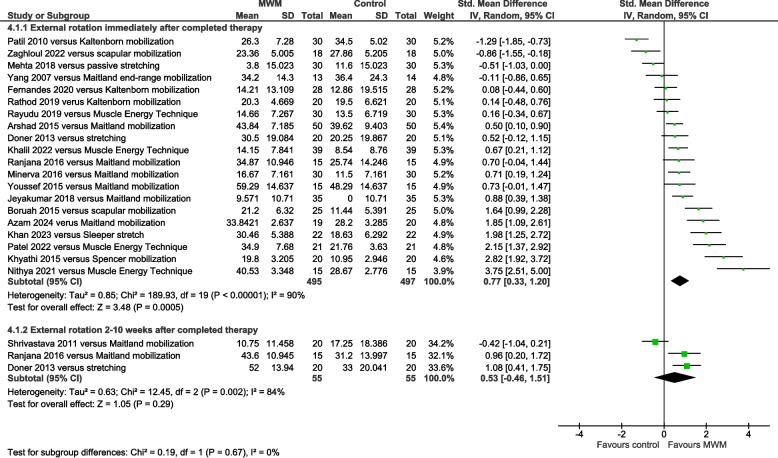
Forest plot of AC external rotation range of motion results immediately post-therapy and 2–10 weeks later – MWM versus other types of mobilization. AC = adhesive capsulitis; MWM = Mobilization With Movement

**Fig. 6:**
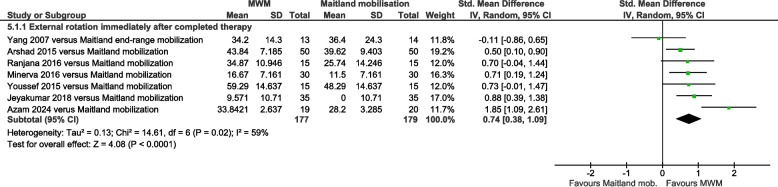
Forest plot of AC external rotation range of motion results immediately post-therapy – MWM versus Maitland mobilization. AC = adhesive capsulitis; MWM = Mobilization With Movement

External rotation ROM was significantly increased by MWM compared with other types of mobilization immediately post-therapy (SMD = 0.77, 95% CI 0.33 to 1.20, I^2^ = 90%, *n* = 992, PEDro score = 5.0), but not 2–10 weeks later (SMD = 0.53, 95% CI −0.46 to 1.51, I^2^ = 84%, *n* = 110, PEDro score = 5.0) (Fig. 5). Compared with Maitland mobilization, external rotation ROM was significantly increased by MWM immediately post-therapy (SMD = 0.74, 95% CI 0.38 to 1.09, I^2^ = 59%, *n* = 356, PEDro score = 5.6) (Fig. 6). Compared with no-intervention control, external rotation ROM was significantly increased by MWM immediately post-therapy (SMD = 1.29, 95% CI 0.88 to 1.71, I^2^ = 48%, *n* = 228, PEDro score = 5.4) (Supplemental Figure S6). A single trial showed that this was also evident 2 weeks later [42].

### AC flexion ROM results

**Fig. 7:**
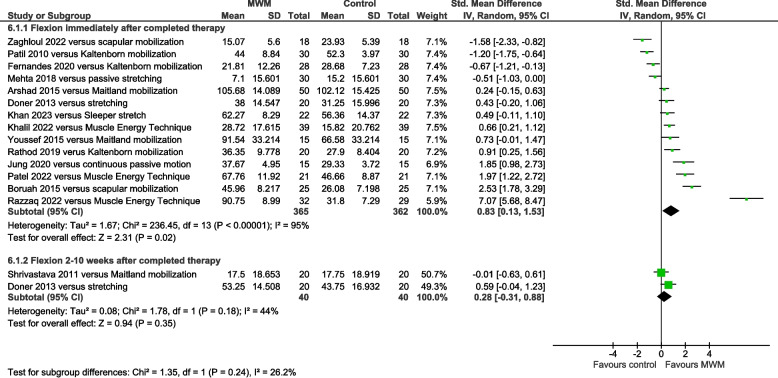
Forest plot of AC flexion range of motion results immediately post-therapy – MWM versus other types of mobilization. AC = adhesive capsulitis; MWM = Mobilization With Movement

Flexion ROM was significantly increased by MWM compared with other types of mobilization immediately post-therapy (SMD = 0.83, 95% CI 0.13 to 1.53, I^2^ = 95%, *n* = 727, PEDro score = 5.1), but not 2–10 weeks later (SMD = 0.28, 95% CI −0.31 to 0.88, I^2^ = 44%, *n* = 80, PEDro score = 5.5) (Fig. 7). Compared with no-intervention control, flexion ROM was significantly increased by MWM immediately post-therapy (SMD = 1.07, 95% CI 0.69 to 1.44, I^2^ = 0%, *n* = 128, PEDro score = 5.3) (Supplemental Figure S7).

### SAPS pain results

Pain was not significantly reduced by MWM compared with sham mobilization immediately post-therapy (SMD = 0.24, 95% CI −0.24 to 0.73, I^2^ = 3%, *n* = 69, PEDro score = 9.0) (Supplemental Figure S8). Compared with no-intervention control, pain was significantly reduced by MWM immediately post-therapy (SMD = 0.98, 95% CI 0.15 to 1.81, I^2^ = 81%, *n* = 168, mean PEDro score = 5.5) (Supplemental Figure S9). In a single trial (*n* = 18), pain did not differ significantly between MWM and Kaltenborn mobilization immediately post-therapy [60].

### SAPS disability results

A single trial showed no significant difference between MWM and sham mobilization for disability immediately post-therapy [62].

### SAPS external rotation ROM results

Immediately post-therapy, external rotation ROM was neither significantly improved by MWM compared with sham mobilization (SMD = 0.56, 95% CI −0.13 to 1.25, I^2^ = 49%, *n* = 69, PEDro score = 9.0) (Supplemental Figure S10) nor compared with no-intervention control (SMD = −0.08, 95% CI −0.44 to 0.27, I^2^ = 0%, *n* = 120, PEDro score = 5.5) (Supplemental Figure S11).

### SAPS flexion ROM results

Flexion ROM was not significantly improved by MWM compared with sham mobilization immediately post-therapy (SMD = 1.02, 95% CI −0.50 to 2.53, I^2^ = 88%, *n* = 69, PEDro score = 9.0) (Supplemental Figure S12). Compared with no-intervention control, flexion ROM was significantly increased by MWM immediately post-therapy (SMD = 0.45, 95% CI 0.11 to 0.79, I^2^ = 0%, *n* = 137, PEDro score = 5.7) (Supplemental Figure S13). In a single trial (*n* = 18), flexion ROM did not differ significantly between MWM and Kaltenborn mobilization immediately post-therapy [60].

### Sensitivity analyses—risk of bias and diabetes status

The immediate post-therapy results of MWM versus other mobilization types in AC trials were subgrouped by risk of bias and diabetes status. In most analyses, overall heterogeneity was comparable to the mean heterogeneity within the subgroups, and no significant differences in effect estimates were observed (Supplemental Figures S14–S20). An exception was noted for disability, where trials with higher PEDro scores demonstrated significantly more favorable results for MWM (Supplemental Figure S15). The remaining meta-analyses included too few trials for a meaningful sensitivity analysis.

## Discussion

To our knowledge, this is the first systematic review focusing on the efficacy of MWM exclusively in chronic shoulder pain. Given the emphasis on chronicity, a considerable number of the patients with AC will have transitioned beyond the freezing stage, the most painful and inflamed period, to the frozen stage characterized by stiffness and restricted ROM due to fibrosis [66]. Beyond three months, acute inflammation typically subsides, and structural or capsular changes become more apparent, allowing clinicians to more reliably distinguish between conditions such as AC and SAPS [66]. Moreover, patients in this stage are thought to tolerate joint mobilization better [66]. No adverse events during the trials were reported, supporting the safety of mobilization techniques in this population.

### AC results

The immediate post-therapy meta-analyses demonstrated significant superiority of MWM over other types of mobilization, showing large effects on pain, disability, and flexion ROM, and a moderate effect on external rotation ROM. However, these results were generally associated with a high level of statistical heterogeneity, lowering our confidence in their precision. Variations in risk of bias and diabetes status did not affect the effect estimates or the heterogeneity meaningfully, suggesting that these factors are unlikely to have undermined the validity of our conclusions. We observed a slight asymmetry in the funnel plots. However, given the head-to-head design of the trials and the high level of statistical heterogeneity, this asymmetry is more likely to reflect methodological heterogeneity or random variation rather than publication bias [34].

Interestingly, MWM significantly outperformed Muscle Energy Techniques in reducing pain, a large effect size immediately post-therapy, although accompanied by with high statistical heterogeneity, warranting cautious interpretation. MWM also demonstrated a moderate and significant superiority over Maitland mobilization in improving external rotation ROM immediately post-therapy, with moderate statistical heterogeneity.

At the 2–10-week follow-up, no significant differences were observed between MWM and other types of mobilization. These results should be interpreted cautiously, as they were based on only 2–3 trials and were associated with moderate to high heterogeneity, which reduces statistical certainty [36].

We also found that MWM was superior to no-intervention control for pain, disability, external rotation ROM, and flexion ROM immediately post-therapy. However, the absence of placebo-controlled trials prohibits drawing strong conclusions about the efficacy of the intervention.

The working mechanisms of MWM in AC are not yet fully understood. In the chronic phase of AC, capsular contracture and coracohumeral ligament thickening are major contributors to restricted ROM [67]. In the AC trial by Jeyakumar et al., MWM significantly reduced coracohumeral ligament thickness compared with no-intervention control, though reductions were smaller than those reported for Maitland mobilization [38]. Because MWM generally involves less external force than other mobilization techniques, these findings suggest that its effects are unlikely to depend primarily on force magnitude, and that structural changes alone may not fully explain its clinical benefits.

### SAPS results

The analyses of SAPS revealed a significant positive effect of MWM compared with no-intervention control on pain and flexion ROM, but no significant effects on disability or external rotation ROM. However, when compared with placebo, MWM showed no significant effects on any of these outcomes. There are several explanations for MWM not showing significant effects in SAPS. The diagnosis encompasses a range of shoulder conditions with varying etiologies, including rotator cuff tendinopathy and bursitis [11, 68]. The included trials likely involved patients with mixed SAPS subtypes, contributing to heterogeneity in treatment responses. The non-significant results may also be due to the small number of placebo-controlled SAPS trials (only two), i.e., lack of statistical power.

### Strengths and limitations of this review

Our review was conducted in line with a published protocol article based on an a priori registration [29], and the selection of trials, risk of bias assessment, and data extraction were handled in a two-person procedure. Since the review included both patient- and therapist-assessed outcomes, we opted to evaluate the risk of bias on the outcome-level, which is a novel strength.

Unlike most prior systematic reviews on the topic, ours encompassed a citation search, and this led to the inclusion of several trials, including one by Patil [49] that had not previously been reviewed [24–27, 69–71]. However, we excluded four trials due to language restrictions. Notably, according to their English abstracts, these trials yielded results generally consistent with our meta-analyses. Specifically, MWM was reportedly better than no-intervention control in one AC trial [72] and in two SAPS trials [73, 74], and superior to conventional physiotherapy in one trial with non-specific shoulder pain [75]. Since none of the abstracts indicated sham mobilization, the exclusion of these trials was unlikely to meaningfully impact our conclusions. In addition, 19 trial reports lacked information on duration of shoulder pain, initially preventing them from being included. After multiple contact attempts, 10 of the author groups provided the necessary information, enabling the inclusion of six of the trials [40, 46, 47, 50, 54, 64]. Of these, four were included in the immediate post-therapy meta-analysis of AC pain, contributing to a slightly less positive result for MWM (Supplemental Figure S22). Additionally, four AC trials that lacked data on participants’ ages [16, 37, 48, 59] were included on the assumption that the mean age did not exceed our exclusion threshold (> 65 years), given that most patients with AC (approximately 84%) are between 40 and 59 years of age [7]. Finally, it should be noted that one trial was excluded due to repeated results, with identical means and standard deviations for post-therapy and follow-up assessments [76]. We sought clarification from the authors about the repeated results. They provided an initial response but did not answer our inquiry.

It is important to acknowledge that many of the trial reports did not describe whether the ROM was assessed actively or passively, or whether it was measured to the first onset of pain (pain-free range) or beyond (end-range). A proportion of the observed heterogeneity could therefore stem from pooling of results obtained with different assessment methods. In the a priori protocol for the review, the specific type of ROM to be analyzed was not explicitly defined. We prioritized active, pain-free ROM, as it provides a more direct reflection of functional capability. The analyses focused on external rotation and flexion, as these were the most frequently assessed movement directions in the included trials.

Furthermore, variations in mobilization types and procedures are additional potential sources of statistical heterogeneity, but the available data did not allow this to be examined.

Finally, it should be noted that our review lacks a QoL analysis, since this outcome was not assessed in any of the trials.

## Conclusions

In individuals with chronic AC, MWM showed significantly greater improvements in pain, disability, and ROM compared with other types of mobilization immediately post-therapy. Subgroup analyses specifically demonstrated that MWM was significantly superior to Muscle Energy Techniques for pain reduction and Maitland mobilization for improving external rotation ROM immediately post-therapy in AC. These results from active comparisons should be interpreted with caution due to statistical heterogeneity. Follow-up data for AC were too sparse to draw reliable conclusions regarding comparative effects. MWM was also significantly better than no-intervention control for pain, disability, and ROM immediately post-therapy in AC. The absence of placebo-controlled studies limits the ability to fully confirm its efficacy in this condition. In chronic SAPS, significant improvements in pain and ROM were observed compared with no-intervention control, but not versus sham mobilization. Due to the limited number of SAPS trials, no firm conclusions can be drawn regarding this population.

## Supplementary Information

Supplementary Material 1.

## Data Availability

The datasets used and/or analyzed during the current study are available from the corresponding author on reasonable request.

## Abbreviations

AC: Adhesive capsulitis
CI: Confidence interval
MWM: Mobilization With Movement
ROM: Range of motion
SAPS: Subacromial pain syndrome
SMD: Standardized mean difference
