# Supplementary material for: Efficacy of mobilization with movement in chronic shoulder pain: a systematic review and meta-analysis of controlled trials
Source: BMC Musculoskelet Disord. 2026 Mar 20;27:374. doi: 10.1186/s12891-025-09422-0 (PMC13137551; doi:10.1186/s12891-025-09422-0)
Supplement: Supplementary file 1 — Supplementary Material 1. [file 12891_2025_9422_MOESM1_ESM.pdf]

BMC Musculoskeletal Disorders supplementary materials

Efficacy of mobilization with movement in chronic shoulder pain: a systematic review and meta-analysis of controlled trials

Arnstein Storås, Fabian Lillebostad, Sturla Haslerud, Jan Magnus Bjordal, Ernesto Cesar Pinto Leal-Junior, Mark I Johnson, Martin Bjørn Stausholm

Contents

PubMed search string....2

Table S1 Studies excluded by full-text evaluation 2

Table S2 Risk of bias judgments – symptoms 4

Table S3 Risk of bias judgments – range of motion 5

Support for risk of bias judgments 6

Figure S1 Funnel plot of AC pain results immediately post-therapy – MWM versus other types of mobilization. 27

Figure S2 Funnel plot of AC external rotation ROM results immediately post-therapy – MWM versus other types of mobilization. 27

Figure S3 Funnel plot of AC flexion ROM results immediately post-therapy – MWM versus other types of mobilization. 28

Figure S4 Forest plot of AC pain results immediately post-therapy – MWM versus no-intervention control 28

Figure S5 Forest plot of AC disability results immediately post-therapy – MWM versus no-intervention control....28

Figure S6 Forest plot of AC external rotation ROM results immediately post-therapy – MWM versus no-intervention control 29

Figure S7 Forest plot of AC flexion ROM results immediately post-therapy – MWM versus no-intervention control....29

Figure S8 Forest plot of SAPS pain results immediately post-therapy – MWM versus sham mobilization 29

Figure S9 Forest plot of SAPS pain results immediately post-therapy – MWM versus no-intervention control 29

Figure S10 Forest plot of SAPS external rotation ROM results immediately post-therapy – MWM versus sham mobilization....30

Figure S11 Forest plot of SAPS external rotation ROM results immediately post-therapy – MWM versus no-intervention control 30

Figure S12 Forest plot of SAPS flexion ROM results immediately post-therapy – MWM versus sham mobilization 30

Figure S13 Forest plot of SAPS flexion ROM results immediately post-therapy – MWM versus no-intervention control....30

Figure S14 Forest plot of AC pain results immediately post-therapy – MWM versus other types of mobilization – subgrouped by risk of bias 31
Figure S15 Forest plot of AC disability results immediately post-therapy – MWM versus other types of mobilization – subgrouped by risk of bias 31
Figure S16 Forest plot of AC external rotation ROM results immediately post-therapy – MWM versus other types of mobilization – subgrouped by risk of bias 32
Figure S17 Forest plot of AC flexion ROM results immediately post-therapy – MWM versus other types of mobilization – subgrouped by risk of bias 32
Figure S18 Forest plot of AC pain results immediately post-therapy – MWM versus other types of mobilization – subgrouped by diabetes status 33
Figure S19 Forest plot of AC disability results immediately post-therapy – MWM versus other types of mobilization – subgrouped by diabetes status 33
Figure S20 Forest plot of AC external rotation ROM results immediately post-therapy – MWM versus other types of mobilization – subgrouped by diabetes status 34
Figure S21 Forest plot of AC flexion ROM results immediately post-therapy – MWM versus other types of mobilization – subgrouped by diabetes status 34
Figure S22 Forest plot of difference in immediate AC pain results between trials with and without duration of pain specified in manuscripts (post-hoc analysis). 35
References 35

PubMed search string

(Shoulder[Title/Abstract] OR Capsulitis[Title/Abstract] OR Bursitis[Title/Abstract] OR Bursitis[Mesh] OR Shoulder[Mesh] OR Shoulder Pain[Mesh] OR Shoulder Impingement Syndrome[Mesh] OR Shoulder Joint[Mesh]) AND (Mulligan[Title/Abstract] OR MWM[Title/Abstract] OR Mobilization*[Title/Abstract] OR Manual therap*[Title/Abstract] OR Manipulation*[Title/Abstract] OR Musculoskeletal Manipulations[Mesh])

Table S1 Studies excluded by full-text evaluation

**First author, reference**	**Reason for exclusion**
Abu 2024 [1]	No shoulder mobilization
Ajis 2024 [2]	No shoulder mobilization
Anwar 2023 [3]	Not chronic pain
Azin 2023 [4]	No MWM
Çelik 2024 [5]	No MWM
Choi 2023 [6]	No MWM
Dabholkar 2013 [7]	No control group
Eliason 2022 [8]	No MWM
Goyal 2013 [9]	Unclear duration of pain – no response to email
Gumucio 2022 [10]	Spanish language
Gutiérrez-Espinoza 2023 [11]	No MWM

Haider 2014 [12]	Not chronic pain
Hasbiah 2018 [13]	Unclear duration of pain – no response to email
Haveela 2018 [14]	Not chronic pain – confirmed via email
Hussein 2019 [15]	No method text section
Jacob 2024 [16]	No MWM
Jain 2023 [17]	No MWM
Jie 2015 [18]	Abstract only
Karakus 2014 [19]	Abstract only
Karasuno 2023 [20]	No MWM
Kazmi 2013 [21]	Unclear duration of pain – no response to email
Khandelwal 2023 [22]	No MWM
Lin 2022 [23]	No MWM
Madhumita 2024 [24]	No relevant outcome
Mariarathinam 2024 [25]	Unclear duration of pain – no response to email
Menek 2018 [26]	Abstract only
Menek 2019 [27]	Not chronic pain – confirmed via email
Michener 2024[28]	Multiple forms of mobilization versus no mobilization
Moradi 2023 [29]	No MWM
Raghav 2019 [30]	Unclear duration of pain – no response to email
Rana 2021 [31]	Unclear duration of pain – no response to email
Razaq 2023 [32]	Numerous repeated results – no response to email
Romero 2015 [33]	Mean age of participants >65 years
Saha 2015 [34]	Unclear duration of pain – no response to email
Satpute 2015 [35]	Not chronic pain
Sharma 2022 [36]	Unclear duration of pain – no response to email
Si 2016 [37]	Chinese language
Silva 2023 [38]	MWM applied in all groups
Srivastava 2017 [39]	No relevant outcome
Srivastava 2018 [40]	Not chronic pain – confirmed via email
Subashini 2024 [41]	Not chronic pain
Subhash 2020 [42]	Not chronic pain
Taghipour 2023 [43]	No MWM
Tauqeer 2024 [44]	No MWM
Teys 2008 [45]	Not chronic pain – confirmed via email
Wang 2018 [46]	Chinese language
Yeonki 2019 [47]	Korean language

MWM = Mobilization With Movement.

Table S2 Risk of bias judgments – symptoms

First author, year	1	2	3	4	5	6	7	8	9	10	11	Total
**Adhesive capsulitis**												
Azam 2024	Yes	+	?	+	-	-	-	+	?	+	+	5
Boruah 2015	Yes	+	?	+	-	-	-	+	?	+	+	5
Dilip 2016	Yes	+	?	+	-	-	-	+	?	+	+	5
Doner 2013	Yes	+	?	+	-	-	-	+	?	+	+	5
Fernandes 2020	Yes	+	?	+	-	-	-	?	?	+	+	4
Jeyakumar 2018	Yes	+	?	+	-	-	-	+	+	+	+	6
Jung 2020	Yes	+	?	+	-	-	-	+	+	+	+	6
Khalil 2022	Yes	+	?	+	-	-	-	+	+	+	+	6
Khan 2023	Yes	+	?	+	-	-	-	?	?	+	+	4
Khyathi 2015	Yes	+	?	+	-	-	-	+	?	+	+	5
Mehta 2018	Yes	+	?	+	-	-	-	?	?	+	+	4
Nithya 2021	Yes	-	-	+	-	-	-	?	?	+	+	3
Pankaj 2013	Yes	+	-	+	-	-	-	?	?	+	+	4
Patel 2022	Yes	-	-	-	-	-	-	+	+	+	+	4
Patil 2010	Yes	+	?	+	-	-	-	+	?	+	+	5
Ranjana 2016	Yes	+	?	+	-	-	-	?	?	+	+	4
Rathod 2019	Yes	+	?	+	-	-	-	?	?	+	+	4
Rayudu 2019	No	+	+	+	-	-	-	?	?	+	+	5
Razzaq 2022	Yes	+	?	+	-	-	-	+	?	+	+	5
Reddy 2015	Yes	+	?	+	-	-	-	?	?	+	+	4
Sai 2015	Yes	+	+	+	-	-	-	+	+	+	+	7
Shera 2023	Yes	+	?	+	-	-	-	+	+	+	+	6
Shrivastava 2011	Yes	+	+	+	-	-	-	?	?	+	+	5
Yang 2007	Yes	+	+	+	-	-	-	+	+	+	+	7
Yeole 2017	Yes	+	?	+	-	-	-	?	?	+	+	4
Zaghloul 2022	Yes	+	?	+	-	-	-	+	?	+	+	5
Mean score		*0.92*	*0.15*	*0.96*	*0.00*	*0.00*	*0.00*	*0.58*	*0.27*	*1.00*	*1.00*	*4.88*
**Subacromial pain syndrome**												
Dalvi 2024	Yes	+	?	+	-	-	-	?	?	+	+	4
Delgado-Gil 2015	Yes	+	+	+	+	-	+	+	+	+	+	9
Guimarães 2016	Yes	+	+	+	+	-	+	+	+	+	+	9
Ishaq 2023	Yes	+	?	+	-	-	-	+	+	+	+	6
Kachingwe 2008	Yes	+	?	+	-	-	-	+	?	+	+	5
Neelapala 2016	Yes	+	+	+	-	-	-	+	+	+	+	7
Mean score		*1*	*0.5*	*1*	*0.33*	*0*	*0.33*	*0.83*	*0.67*	*1*	*1*	*6.67*

1. Eligibility criteria specified (not included in the total score).
2. Random allocation.
3. Concealed allocation.
4. Groups similar at baseline.
5. Subject blinding.
6. Therapist blinding.
7. Assessor blinding.
8. Less than 15% dropout.
9. Intention-to-treat analysis.
10. Between-group statistical comparisons.
11. Point measures and variability data.

Table S3 Risk of bias judgments – range of motion

First author, year	1	2	3	4	5	6	7	8	9	10	11	Total
**Adhesive capsulitis**												
Arshad 2015	Yes	+	?	+	-	-	?	+	?	+	+	5
Azam 2024	Yes	+	?	+	-	-	?	+	?	+	+	5
Boruah 2015	Yes	+	?	+	-	-	?	+	?	+	+	5
Doner 2013	Yes	+	?	+	-	-	?	+	?	+	+	5
Fernandes 2020	Yes	+	?	+	-	-	?	?	?	+	+	4
Jeyakumar 2018	Yes	+	?	+	-	-	+	+	+	+	+	7
Jung 2020	Yes	+	?	+	-	-	+	+	+	+	+	7
Khalil 2022	Yes	+	?	+	-	-	+	+	+	+	+	7
Khan 2023	Yes	+	?	+	-	-	?	?	?	+	+	4
Khyathi 2015	Yes	+	?	+	-	-	-	+	?	+	+	5
Mehta 2018	Yes	+	?	+	-	-	?	?	?	+	+	4
Minerva 2016	Yes	+	+	+	-	-	?	?	?	+	+	5
Nithya 2021	Yes	-	-	+	-	-	?	?	?	+	+	3
Patel 2022	Yes	-	-	+	-	-	?	+	+	+	+	5
Patil 2010	Yes	+	?	+	-	-	-	+	?	+	+	5
Ranjana 2016	Yes	+	?	+	-	-	?	?	?	+	+	4
Rathod 2019	Yes	+	?	+	-	-	?	?	?	+	+	4
Rayudu 2019	No	+	+	+	-	-	?	?	?	+	+	5
Razzaq 2022	Yes	+	?	+	-	-	+	+	?	+	+	6
Reddy 2015	Yes	+	?	+	-	-	-	?	?	+	+	4
Sai 2015	Yes	+	+	+	-	-	+	+	+	+	+	8
Shrivastava 2011	Yes	+	+	+	-	-	+	?	?	+	+	6
Yang 2007	Yes	+	+	+	-	-	+	+	+	+	+	8
Yeole 2017	Yes	+	?	+	-	-	?	?	?	+	+	4
Youssef 2015	Yes	+	+	+	-	-	?	?	?	+	+	5
Zaghloul 2022	Yes	+	?	+	-	-	?	+	?	+	+	5
Mean score		*0.92*	*0.23*	*1.00*	*0.00*	*0.00*	*0.27*	*0.54*	*0.23*	*1.00*	*1.00*	*5.19*
**Subacromial pain syndrome**												
Dalvi 2024	Yes	+	?	+	-	-	?	?	?	+	+	4
Delgado-Gil 2015	Yes	+	+	+	+	-	+	+	+	+	+	9
Guimarães 2016	Yes	+	+	+	+	-	+	+	+	+	+	9
Ishaq 2023	Yes	+	?	+	-	-	+	+	+	+	+	7
Kachingwe 2008	Yes	+	?	+	-	-	+	+	?	+	+	6
Mean score		*1.00*	*0.40*	*1.00*	*0.40*	*0.00*	*0.80*	*0.80*	*0.60*	*1.00*	*1.00*	*7.00*

1. Eligibility criteria specified (not included in the total score).
2. Random allocation.
3. Concealed allocation.
4. Groups similar at baseline.
5. Subject blinding.
6. Therapist blinding.
7. Assessor blinding.
8. Less than 15% dropout.
9. Intention-to-treat analysis.
10. Between-group statistical comparisons.
11. Point measures and variability data.

*Item not included in the total score.

Support for risk of bias judgments

Arshad et al. 2015

Type of bias	Judgment	Support for judgment
Eligibility criteria were specified	Yes	Page 2764.
Subjects were randomly allocated to groups	Yes	Quote: "All the subjects was interviewed and evaluated for inclusion, and they signed the consent form then they were asked to pick up a card for entitlement randomly in each of two groups. i.e., either group A or B and was included in the study."
Allocation was concealed	Unclear	Not enough information to make a qualified judgment.
The groups were similar at baseline	Yes	Tables 1 and 2.
There was blinding of all subjects	No	Not feasible.
There was blinding of all therapists	No	Not feasible.
There was blinding of all assessors – symptoms	-	Symptoms not assessed.
There was blinding of all assessors – range of motion	Unclear	Not enough information to make a qualified judgment.
Measures of at least one key outcome obtained >85% of the participants	Yes	Table 6.
Intention-to-treat	Unclear	Not enough information to make a qualified judgment.
The results of between-group statistical comparisons are reported for at least one key outcome	Yes	Table 6.
The study provides both point measures and measures of variability for at least one key outcome	Yes	Table 6.

Azam et al. 2024

Type of bias	Judgment	Support for judgment
Eligibility criteria were specified	Yes	Page 20.
Subjects were randomly allocated to groups	Yes	Quote: "A randomized clinical trial was performed using a nonprobability convenient sampling technique."
Allocation was concealed	Unclear	Not enough information to make a qualified judgment.
The groups were similar at baseline	Yes	Pain varied between 6.3684 and 6.7000, and external rotation varied between 31.3158 and 31.6500.
There was blinding of all subjects	No	Not feasible.
There was blinding of all therapists	No	Not feasible.
There was blinding of all assessors – symptoms	No	Not feasible.
There was blinding of all assessors – range of motion	Unclear	Too little information to make a qualified judgment.
Measures of at least one key outcome obtained >85% of the participants	Yes	Quote: "Forty participants were assessed for eligibility". Tables 2-3 displays results from 39 participants.
Intention-to-treat	Unclear	Too little information to make a qualified judgment.

The results of between-group statistical comparisons are reported for at least one key outcome	Yes	Tables 2-3.
The study provides both point measures and measures of variability for at least one key outcome	Yes	Tables 2-3.

Boruah et al. 2015

Type of bias	Judgment	Support for judgment
Eligibility criteria were specified	Yes	Page 813.
Subjects were randomly allocated to groups	Yes	Quote: "25 subjects were assigned in two groups randomly."
Allocation was concealed	Unclear	Not enough information to make a qualified judgment.
The groups were similar at baseline	Yes	Table 2.
There was blinding of all subjects	No	Not feasible.
There was blinding of all therapists	No	Not feasible.
There was blinding of all assessors – symptoms	No	Not feasible.
There was blinding of all assessors – range of motion	Unclear	Not enough information to make a qualified judgment.
Measures of at least one key outcome obtained >85% of the participants	Yes	Table 5.
Intention-to-treat	Unclear	Not enough information to make a qualified judgment.
The results of between-group statistical comparisons are reported for at least one key outcome	Yes	Table 5.
The study provides both point measures and measures of variability for at least one key outcome	Yes	Table 5.

Dalvi et al. 2024

Type of bias	Judgment	Support for judgment
Eligibility criteria were specified	Yes	Page 2
Subjects were randomly allocated to groups	Yes	Quote: "The individuals were randomly divided into two groups, namely Group A and Group B, using a simple random sampling method through IBM SPSS Statistics for Windows, Version 26 (Released 2019; IBM Corp., Armonk, NY, USA). This software was employed to ensure a random and unbiased allocation of participants to each group (…)."
Allocation was concealed	Unclear	Not enough information to make a qualified judgment.
The groups were similar at baseline	Yes	Tables 3-4.
There was blinding of all subjects	No	The authors mention that a double-blind procedure was used. There is no mention of any blinding procedures in the manuscript. It is possible to blind the patients, therapist, assessor, and statistician, rendering the term double-blinding useless without further specification. The authors also stated "(…)

		Group A, which was administered conventional therapy alone.", indicating that this control group did not receive sham mobilization.
There was blinding of all therapists	No	Not feasible.
There was blinding of all assessors – symptoms	No	The patients who assessed their own symptoms were not blinded.
There was blinding of all assessors – range of motion	Unclear	Too little information to make a qualified judgment.
Measures of at least one key outcome obtained >85% of the participants	Unclear	Too little information to make a qualified judgment.
Intention-to-treat	Unclear	Too little information to make a qualified judgment.
The results of between-group statistical comparisons are reported for at least one key outcome	Yes	Tables 3-4.
The study provides both point measures and measures of variability for at least one key outcome	Yes	Tables 3-4.

Delgado-Gil et al. 2015

Type of bias	Judgment	Support for judgment
Eligibility criteria were specified	Yes	Page 246.
Subjects were randomly allocated to groups	Yes	Quote: "Concealed allocation was performed using a computer generated randomized table of numbers (…)"
Allocation was concealed	Yes	Quotes: "Concealed allocation was performed using a computer generated randomized table of numbers (…)" and "A second therapist, blinded to baseline examination findings, opened the envelope and proceeded with treatment according to the group assignment."
The groups were similar at baseline	Yes	Table 1.
There was blinding of all subjects	Yes	Quote: "The absence of previous experience with manual therapy applied to the shoulder of all participants (naive) assisted with patient blinding. Patients were informed that the current study investigated the effects of manual handling on shoulder pain, without any information of the real objective of testing-specific technique effects."
There was blinding of all therapists	No	Quote: "A second therapist, blinded to baseline examination findings, opened the envelope and proceeded with treatment according to the group assignment.". The therapist was aware of the group allocation.
There was blinding of all assessors – symptoms	Yes	Quote: "Outcomes were taken by an assessor blinded to group allocation at baseline and 24-hour postintervention (after 4 treatments over a 2-week timeframe).". Furthermore, the participants were blinded and scored their own symptoms.
There was blinding of all assessors – range of motion	Yes	Quote: "(…) active range of motion were assessed by a clinician blinded to group allocation."

Measures of at least one key outcome obtained >85% of the participants	Yes	Figure 5.
Intention-to-treat	Yes	Figure 5.
The results of between-group statistical comparisons are reported for at least one key outcome	Yes	Table 2.
The study provides both point measures and measures of variability for at least one key outcome	Yes	Table 2.

Dilip et al. 2016

Type of bias	Judgment	Support for judgment
Eligibility criteria were specified	Yes	Page 133.
Subjects were randomly allocated to groups	Yes	Quote: "Subjects who meet inclusion criteria were recruited by simple random sampling method using closed envelops (…)"
Allocation was concealed	Unclear	It is unclear whether the envelopes were opaque.
The groups were similar at baseline	Yes	Table 2.
There was blinding of all subjects	No	Not feasible.
There was blinding of all therapists	No	Not feasible.
There was blinding of all assessors – symptoms	No	Not feasible.
There was blinding of all assessors – range of motion	NA	Not applicable.
Measures of at least one key outcome obtained >85% of the participants	Yes	Quote: "An Experimental study design, 40 subjects with unilateral frozen shoulder were selected and randomized (...)" and quote: "Total 40 Subject (n=40), 20 in each group completed the studied."
Intention-to-treat	Unclear	Not enough information to make a qualified judgment.
The results of between-group statistical comparisons are reported for at least one key outcome	Yes	Table 2.
The study provides both point measures and measures of variability for at least one key outcome	Yes.	Table 2.

Doner et al. 2013

Type of bias	Judgment	Support for judgment
Eligibility criteria were specified	Yes	Page 87.
Subjects were randomly allocated to groups	Yes	Quote: "A total of 40 subjects were randomized using a table of random numbers."
Allocation was concealed	Unclear	Not enough information to make a qualified judgment.
The groups were similar at baseline	Yes	Table 1.
There was blinding of all subjects	No	Quote: "Since Mulligan's technique is a hands-on treatment it is not possible to perform since Mulligan's technique is a hands-on treatment it is not possible to perform."

There was blinding of all therapists	No	Quote: "since the study is about a manual treatment, the therapy is applied by the same experienced therapist."
There was blinding of all assessors – symptoms	No	Not feasible.
There was blinding of all assessors – range of motion	Unclear	Not enough information to make a qualified judgment.
Measures of at least one key outcome obtained >85% of the participants	Yes	Table 4.
Intention-to-treat	Unclear	Not enough information to make a qualified judgment.
The results of between-group statistical comparisons are reported for at least one key outcome	Yes	Table 2.
The study provides both point measures and measures of variability for at least one key outcome	Yes	Table 2.

Fernandes et al. 2020

Type of bias	Judgment	Support for judgment
Eligibility criteria were specified	Yes	Page 19.
Subjects were randomly allocated to groups	Yes	Quote: "Study design, duration A Prospective Randomized Study, 1 year."
Allocation was concealed	Unclear	Not enough information to make a qualified judgment.
The groups were similar at baseline	Yes	Table 2 and 3.
There was blinding of all subjects	No	Quote: "Limitations of the study: 1. Lack of subject blinding (…)"
There was blinding of all therapists	No	Not feasible.
There was blinding of all assessors – symptoms	No	Not feasible.
There was blinding of all assessors – range of motion	Unclear	Not enough information to make a qualified judgment.
Measures of at least one key outcome obtained >85% of the participants	Unclear	Not enough information to make a qualified judgment.
Intention-to-treat	Unclear	Not enough information to make a qualified judgment.
The results of between-group statistical comparisons are reported for at least one key outcome	Yes	Tables 2-3.
The study provides both point measures and measures of variability for at least one key outcome	Yes	Tables 2-3.

Guimarães et al. 2016

Type of bias	Judgment	Support for judgment
Eligibility criteria were specified	Yes	Page 606.
Subjects were randomly allocated to groups	Yes	Quote: "The randomization of the individuals to groups, as well as the order of ROM assessments and the dynamometry tests, were conducted before the beginning of the research using the website randomization.com."

Allocation was concealed	Yes	Quote: "The randomization of the individuals to groups, as well as the order of ROM assessments and the dynamometry tests, were conducted before the beginning of the research using the website randomization.com."
The groups were similar at baseline	Yes	Table 1.
There was blinding of all subjects	Yes	Quote: "The sham condition replicated the treatment condition with the therapist using a different hand positioning."
There was blinding of all therapists	No	The therapist knew who received the real and sham intervention.
There was blinding of all assessors – symptoms	Yes	The participants were blinded and scored their own symptoms.
There was blinding of all assessors – range of motion	Yes	Quote: "Assessments were performed by Researcher A (ALSJ), who was blinded to the participant allocation."
Measures of at least one key outcome obtained >85% of the participants	Yes	Figure 5.
Intention-to-treat	Yes	Figure 5.
The results of between-group statistical comparisons are reported for at least one key outcome	Yes	Table 2.
The study provides both point measures and measures of variability for at least one key outcome	Yes	Table 2.

Ishaq et al. 2023

Type of bias	Judgment	Support for judgment
Eligibility criteria were specified	Yes	Page 16
Subjects were randomly allocated to groups	Yes	Quote: "Online randomization was performed through a web service (www.randomizer.org)."
Allocation was concealed	Unclear	Too little information to make a qualified judgment.
The groups were similar at baseline	Yes	Table 2.
There was blinding of all subjects	No	Not feasible.
There was blinding of all therapists	No	Not feasible.
There was blinding of all assessors – symptoms	No	The patients who assessed their own symptoms were not blinded.
There was blinding of all assessors – range of motion	Yes	Quote: "All outcomes were measured by the senior consultant physical therapist, who was blinded for the treatment regimen given to any particular patient."
Measures of at least one key outcome obtained >85% of the participants	Yes	Figure 1.
Intention-to-treat	Yes	Figure 1.
The results of between-group statistical comparisons are reported for at least one key outcome	Yes	Table 2.
The study provides both point measures and measures of variability for at least one key outcome	Yes	Table 2.

Jeyakumar et al. 2018

Type of bias	Judgment	Support for judgment
Eligibility criteria were specified	Yes	Page 2.
Subjects were randomly allocated to groups	Yes	Quote: "Patients were included in the study after signing informed consent and randomly assigned to Group A and Group Band Group C."
Allocation was concealed	Unclear	It is unclear whether the envelopes were opaque.
The groups were similar at baseline	Yes	Table 2.
There was blinding of all subjects	No	Not feasible.
There was blinding of all therapists	No	Not feasible.
There was blinding of all assessors – symptoms	No	Not feasible.
There was blinding of all assessors – range of motion	Yes	As this was a head-to-head study and the authors mentioned that it was a "Randomized single-blinded controlled clinical trial.", it is reasonable to assume that the assessor was blinded.
Measures of at least one key outcome obtained >85% of the participants	Yes	Figure 6-8.
Intention-to-treat	Yes	Quote: "All the participants have received the intervention for a period of 4 weeks (…)"
The results of between-group statistical comparisons are reported for at least one key outcome	Yes	Table 2.
The study provides both point measures and measures of variability for at least one key outcome	Yes	Table 2.

Jung and Chung 2020

Type of bias	Judgment	Support for judgment
Eligibility criteria were specified	Yes	Quote: "Forty-five surgical patients with shoulder adhesive capsulitis participated in this study and were randomly divided into three groups (…)"
Subjects were randomly allocated to groups	Yes	Page 90.
Allocation was concealed	Unclear	Not enough information to make a qualified judgment.
The groups were similar at baseline	Yes	Table 1.
There was blinding of all subjects	No	Not feasible.
There was blinding of all therapists	No	Not feasible.
There was blinding of all assessors – symptoms	No	Not feasible.
There was blinding of all assessors – range of motion	Yes	Quote: "One physical therapist with over 10 years of experience in the treatment of shoulder joint surgery was evaluated using a single blind method without knowing about the group of each patient."
Measures of at least one key outcome obtained >85% of the participants	Yes	Figure 1.
Intention-to-treat	Yes	Figure 1.

The results of between-group statistical comparisons are reported for at least one key outcome	Yes	Table 2.
The study provides both point measures and measures of variability for at least one key outcome	Yes	Table 2.

Khalil et al. 2022

Type of bias	Judgment	Support for judgment
Eligibility criteria were specified	Yes	Page 212
Subjects were randomly allocated to groups	Yes	Quote: "After taking the consent from participants the subjects were randomised using the lottery method into MMT Group A and MET Group B."
Allocation was concealed	Unclear	Not enough information to make a qualified judgment.
The groups were similar at baseline	Yes	Table 3.
There was blinding of all subjects	No	Not feasible.
There was blinding of all therapists	No	Not feasible.
There was blinding of all assessors – symptoms	No	Not feasible.
There was blinding of all assessors – range of motion	Yes	Quote: "The outcome assessor was kept blinded to the treatment plan."
Measures of at least one key outcome obtained >85% of the participants	Yes	Figure 3.
Intention-to-treat	Yes	Flow chart.
The results of between-group statistical comparisons are reported for at least one key outcome	Yes	Table 3.
The study provides both point measures and measures of variability for at least one key outcome	Yes	Table 3.

Kachingwe et al. 2013

Type of bias	Judgment	Support for judgment
Eligibility criteria were specified	Yes	Page 239.
Subjects were randomly allocated to groups	Yes	Quote: "Participants were randomly assigned to one of four intervention groups according to the block randomization method."
Allocation was concealed	Unclear	Not enough information to make a qualified judgment.
The groups were similar at baseline	Yes	Table 1.
There was blinding of all subjects	No	Quote: "Each subject was informed of his/her treatment protocol (…)"
There was blinding of all therapists	No	Quote: "The principle investigator (…) performed all treatment interventions." The therapist knew who received the different interventions.
There was blinding of all assessors – symptoms	No	The participants were not blinded, and they scored their own symptoms.
There was blinding of all assessors – range of motion	Yes	Quote: "This assessor was blinded to group assignment and all intervention protocols."

Measures of at least one key outcome obtained >85% of the participants	Yes	There were no dropouts reported (Tables 1-2).
Intention-to-treat	Unclear	Not enough information to make a qualified judgment.
The results of between-group statistical comparisons are reported for at least one key outcome	Yes	Table 2.
The study provides both point measures and measures of variability for at least one key outcome	Yes	Table 2.

Khan et al. 2023

Type of bias	Judgment	Support for judgment
Eligibility criteria were specified	Yes	Page 4.
Subjects were randomly allocated to groups	Yes	Quote: "A Randomized controlled trial (RCT) was conducted (…)"
Allocation was concealed	Unclear	It is unclear whether the envelopes were opaque.
The groups were similar at baseline	Yes	Figure 1.
There was blinding of all subjects	No	Not feasible.
There was blinding of all therapists	No	Not feasible.
There was blinding of all assessors – symptoms	No	Not feasible.
There was blinding of all assessors – range of motion	Unclear	Too little information to make a qualified judgment.
Measures of at least one key outcome obtained >85% of the participants	Unclear	Too little information to make a qualified judgment.
Intention-to-treat	Unclear	Too little information to make a qualified judgment.
The results of between-group statistical comparisons are reported for at least one key outcome	Yes	Pages 5-6.
The study provides both point measures and measures of variability for at least one key outcome	Yes	Pages 5-6.

Khyathi et al. 2015

Type of bias	Judgment	Support for judgment
Eligibility criteria were specified	Yes	Pages 449-450.
Subjects were randomly allocated to groups	Yes	Quote: "Subjects who meet inclusion criteria were recruited by simple random sampling method using closed envelops, randomly allocated subjects into two groups."
Allocation was concealed	Unclear	It is unclear whether the envelopes were opaque.
The groups were similar at baseline	Yes	Tables 2-3.
There was blinding of all subjects	No	Quote: "Subjects were blinded throughout the treatment sessions, subjects from both the groups were not allowed to have any interaction to each other and the subjects were not aware of what kind of treatment they received (…)". While this may reduce bias to some point, the placebo effects of

		mobilization with movement and Spencer mobilization may differ.
There was blinding of all therapists	No	It says it was a single blind study in the title.
There was blinding of all assessors – symptoms	No	The participants were not truly blinded.
There was blinding of all assessors – range of motion	No	The study is described as a single-blind trial. Since the authors stated that the subjects were blinded, the assessor was not. Also, there is no mention of assessor blinding in the report.
Measures of at least one key outcome obtained >85% of the participants	Yes	Quote: "An experimental study design, 40 subjects with unilateral frozen shoulder were randomized into 2 groups with 20 subjects each (…)" and Quote: "The study was completed with total 40 subjects (Table 1)."
Intention-to-treat	Unclear	Not enough information to make a qualified judgment.
The results of between-group statistical comparisons are reported for at least one key outcome	Yes	Table 4.
The study provides both point measures and measures of variability for at least one key outcome	Yes	Table 4.

Mehta et al. 2018

Type of bias	Judgment	Support for judgment
Eligibility criteria were specified	Yes	Page 2786.
Subjects were randomly allocated to groups	Yes	Quote: "60 patients, both male and female, of the age group 40-60 years, diagnosed with Adhesive Capsulitis were distributed by Stratified Random Allocation (…)"
Allocation was concealed	Unclear	Not enough information to make a qualified judgment.
The groups were similar at baseline	Yes	Page 2787.
There was blinding of all subjects	No	Not feasible.
There was blinding of all therapists	No	Not feasible.
There was blinding of all assessors – symptoms	No	Not feasible.
There was blinding of all assessors – range of motion	Unclear	Not enough information to make a qualified judgment.
Measures of at least one key outcome obtained >85% of the participants	Unclear	Not enough information to make a qualified judgment.
Intention-to-treat	Unclear	Not enough information to make a qualified judgment.
The results of between-group statistical comparisons are reported for at least one key outcome	Yes	Page 2787.
The study provides both point measures and measures of variability for at least one key outcome	Yes	Page 2787.

Minerva et al. 2016

Type of bias	Judgment	Support for judgment
Eligibility criteria were specified	Yes	Page 238.
Subjects were randomly allocated to groups	Yes	Quote: "A concealed block randomization was done and finally each group ended up with 30 subjects each."
Allocation was concealed	Yes	Quote: "A concealed block randomization was done and finally each group ended up with 30 subjects each."
The groups were similar at baseline	Yes	Tables 5-7.
There was blinding of all subjects	No	Not feasible.
There was blinding of all therapists	No	Not feasible.
There was blinding of all assessors – symptoms	-	Symptoms not assessed.
There was blinding of all assessors – range of motion	Unclear	Not enough information to make a qualified judgment.
Measures of at least one key outcome obtained >85% of the participants	Unclear	Not enough information to make a qualified judgment.
Intention-to-treat	Unclear	Not enough information to make a qualified judgment.
The results of between-group statistical comparisons are reported for at least one key outcome	Yes	Table 9.
The study provides both point measures and measures of variability for at least one key outcome	No	Table 9.

Neelapala et al. 2016

Type of bias	Judgment	Support for judgment
Eligibility criteria were specified	Yes	Page 3.
Subjects were randomly allocated to groups	Yes	Quote: "Randomization was done using a computer generated sequence (…)"
Allocation was concealed	Yes	Quote: "allocation concealment of participants was done using sequentially numbered closed envelopes." It is not stated in the report whether the envelopes were opaque, but the trial has been indexed with a "yes" for allocation concealment in the PEDro database.
The groups were similar at baseline	Yes	Table 2.
There was blinding of all subjects	No	Quote: "The study is a single-blind randomized controlled trial where the outcome assessor was blinded (…)"
There was blinding of all therapists	No	Only the outcome assessor was blinded, i.e., a staff member.
There was blinding of all assessors – symptoms	No	The patient scored their own symptoms, and the patients were not blinded.
There was blinding of all assessors – range of motion	NA	Range of motion not included as an outcome.
Measures of at least one key outcome obtained >85% of the participants	Yes	Figure 1.
Intention-to-treat	Yes	Figure 1.
The results of between-group statistical comparisons are reported for at least one key outcome	Yes	Table 3.

The study provides both point measures and measures of variability for at least one key outcome	Yes	Table 3.

Nithya et al. 2021

Type of bias	Judgment	Support for judgment
Eligibility criteria were specified	Yes	Page 186.
Subjects were randomly allocated to groups	No	Quote: “30 subjects were selected and divided into 2 groups of 15 subjects in each group by purposive sampling method.”
Allocation was concealed	No	Quote: “30 subjects were selected and divided into 2 groups of 15 subjects in each group by purposive sampling method.”
The groups were similar at baseline	Yes	Table 1.
There was blinding of all subjects	No	Not feasible.
There was blinding of all therapists	No	Not feasible.
There was blinding of all assessors – symptoms	No	Not feasible.
There was blinding of all assessors – range of motion	Unclear	Not enough information to make a qualified judgment.
Measures of at least one key outcome obtained >85% of the participants	Unclear	Not enough information to make a qualified judgment.
Intention-to-treat	Unclear	Not enough information to make a qualified judgment.
The results of between-group statistical comparisons are reported for at least one key outcome	Yes	Page 187.
The study provides both point measures and measures of variability for at least one key outcome	Yes	Table 2.

Pankaj et al. 2013

Type of bias	Judgment	Support for judgment
Eligibility criteria were specified	Yes	Page 229.
Subjects were randomly allocated to groups	Yes	Quote: “Randomization was done by using thirty pieces of paper with 15 pieces having the words Gong’s Mobilization written on them, 15 pieces having the words MWM Mobilization. All pieces of paper were tightly folded and placed in a box. After shaking the box thoroughly, piece of paper was withdrawn, each piece of paper individually having the group name on it would be added to a list that corresponded with patient numbers from 1-30.”
Allocation was concealed	No	Researchers and individuals enrolling participants may have been aware of the group assignment because they could see the words "Gong’s Mobilization" or "MWM Mobilization" written on the pieces of paper before they withdrew from the box.
The groups were similar at baseline	Yes	Table 3.
There was blinding of all subjects	No	Not feasible.
There was blinding of all therapists	No	Not feasible.

There was blinding of all assessors – symptoms	No	Not feasible.
There was blinding of all assessors – range of motion	Unclear	Not enough information to make a qualified judgment.
Measures of at least one key outcome obtained >85% of the participants	Unclear	Not enough information to make a qualified judgment.
Intention-to-treat	Unclear	Not enough information to make a qualified judgment.
The results of between-group statistical comparisons are reported for at least one key outcome	Yes	Table 2.
The study provides both point measures and measures of variability for at least one key outcome	Yes	Table 2.

Patel et al. 2022

Type of bias	Judgment	Support for judgment
Eligibility criteria were specified	Yes	Page 396.
Subjects were randomly allocated to groups	No	Quote: "Subjects were allocated into two groups, group A (MET) and group B (MWM) by using quasi-randomization procedure as follows. First subject with ACS was allocated to Group A, second visiting subject to Group B once they fulfilled the inclusion and exclusion criteria. The same sequence of procedure was followed throughout for consecutive subjects."
Allocation was concealed	No	Quote: "First subject with ACS was allocated to Group A, second visiting subject to Group B once they fulfilled the inclusion and exclusion criteria. The same sequence of procedure was followed throughout for consecutive subjects."
The groups were similar at baseline	No and yes	The baseline pain intensity differed substantially; 4.95 and 3.05 in the Mulligan and control group, respectively (Tables 3-4) . However, the participants were similar at baseline in terms of range of motion (Tables 3-4).
There was blinding of all subjects	No	Not feasible.
There was blinding of all therapists	No	Not feasible.
There was blinding of all assessors – symptoms	No	Not feasible.
There was blinding of all assessors – range of motion	Unclear	Not enough information to make a qualified judgment.
Measures of at least one key outcome obtained >85% of the participants	Yes	According to table 6, all the participants were assessed post-therapy.
Intention-to-treat	Yes	Not needed as all the participants were assessed post-therapy. Also, it is highly unlikely that any of the participants shifted treatment group.
The results of between-group statistical comparisons are reported for at least one key outcome	Yes	Table 6.

The study provides both point measures and measures of variability for at least one key outcome	Yes	Table 6.

Patil 2010

Type of bias	Judgment	Support for judgment
Eligibility criteria were specified	Yes	Page 38.
Subjects were randomly allocated to groups	Yes	Quote: "A simple random method was used for assigning patients in to two groups. Group A (n=30 Group B (n=30) using (envelope method)."
Allocation was concealed	Unclear	It is unclear whether the envelopes were sealed and opaque.
The groups were similar at baseline	Yes	Table 4.
There was blinding of all subjects	No	Not feasible.
There was blinding of all therapists	No	Not feasible.
There was blinding of all assessors – symptoms	No	Not feasible.
There was blinding of all assessors – range of motion	No	Quote: "No blinding of evaluators of outcomes was done."
Measures of at least one key outcome obtained >85% of the participants	Yes	Quote: "All subjects received 6 therapy sessions consisting of application of therapeutic ultrasound, joint mobilization, and upper-body ergometer exercise.". Since all the subjects completed the study, more than 85% of them were probably reassessed.
Intention-to-treat	Unclear	Not enough information to make a qualified judgment.
The results of between-group statistical comparisons are reported for at least one key outcome	Yes	Table 4.
The study provides both point measures and measures of variability for at least one key outcome	Yes	Table 4.

Ranjana et al. 2016

Type of bias	Judgment	Support for judgment
Eligibility criteria were specified	Yes	Page 92.
Subjects were randomly allocated to groups	Yes	Quote: "Subjects were allocated to three different treatment groups, Group A (Maitland's mobilization along with supervised exercise program), Group B (Mulligan's mobilization along with supervised exercise program) and Group C (Supervised exercise program alone) by simple random sampling (chit picking method), consisting of 15 subjects in each group."
Allocation was concealed	Unclear	It is unclear whether the chit picking was done in a way that enables concealed allocation.
The groups were similar at baseline	Yes	Table 1.
There was blinding of all subjects	No	Not feasible.
There was blinding of all therapists	No	Not feasible.

There was blinding of all assessors – symptoms	No	Not feasible.
There was blinding of all assessors – range of motion	Unclear	Not enough information to make a qualified judgment.
Measures of at least one key outcome obtained >85% of the participants	Unclear	Not enough information to make a qualified judgment.
Intention-to-treat	Unclear	Not enough information to make a qualified judgment.
The results of between-group statistical comparisons are reported for at least one key outcome	Yes	Table 1.
The study provides both point measures and measures of variability for at least one key outcome	Yes	Table 1.

Rathod et al. 2019

Type of bias	Judgment	Support for judgment
Eligibility criteria were specified	Yes	Page 322.
Subjects were randomly allocated to groups	Yes	Quote: "The subjects were divided randomly into two equal groups."
Allocation was concealed	Unclear	Not enough information to make a qualified judgment.
The groups were similar at baseline	Yes	Table 1.
There was blinding of all subjects	No	Not feasible.
There was blinding of all therapists	No	Not feasible.
There was blinding of all assessors – symptoms	No	Not feasible.
There was blinding of all assessors – range of motion	Unclear	Not enough information to make a qualified judgment.
Measures of at least one key outcome obtained >85% of the participants	Unclear	Not enough information to make a qualified judgment.
Intention-to-treat	Unclear	Not enough information to make a qualified judgment.
The results of between-group statistical comparisons are reported for at least one key outcome	Yes	Table 2.
The study provides both point measures and measures of variability for at least one key outcome	Yes	Table 2.

Rayudu et al. 2019

Type of bias	Judgment	Support for judgment
Eligibility criteria were specified	No	Not reported.
Subjects were randomly allocated to groups	Yes	Quote: "The study design was experimental, and randomisation of the individuals were done by using concealed block randomisation."
Allocation was concealed	Yes	Quote: "The study design was experimental, and randomisation of the individuals were done by using concealed block randomisation."
The groups were similar at baseline	Yes	Table 1.
There was blinding of all subjects	No	Not feasible.
There was blinding of all therapists	No	Not feasible.

There was blinding of all assessors – symptoms	No	Not feasible.
There was blinding of all assessors – range of motion	Unclear	Not enough information to make a qualified judgment.
Measures of at least one key outcome obtained >85% of the participants	Unclear	Not enough information to make a qualified judgment.
Intention-to-treat	Unclear	Not enough information to make a qualified judgment.
The results of between-group statistical comparisons are reported for at least one key outcome	Yes	Page 25640.
The study provides both point measures and measures of variability for at least one key outcome	Yes	Table 1.

Razzaq et al. 2022

Type of bias	Judgment	Support for judgment
Eligibility criteria were specified	Yes	Page 14.
Subjects were randomly allocated to groups	Yes	Quote: "After taking informed consent from the subjects included, they were randomised using the lottery method into MWM group A and MET group B."
Allocation was concealed	Unclear	Not enough information to make a qualified judgment.
The groups were similar at baseline	Yes	Table 1.
There was blinding of all subjects	No	Quote: "The single-blind (in which assessor was kept blind), randomised controlled trial (…)". No attempt to blind the subjects was made.
There was blinding of all therapists	No	Quote: "The single-blind (in which assessor was kept blind), randomised controlled trial (…)". No attempt to blind the therapist was made.
There was blinding of all assessors – symptoms	No	Quote: "The single-blind (in which assessor was kept blind), randomised controlled trial (…)". The patients who scored their own pain were not blinded.
There was blinding of all assessors – range of motion	Yes	Quote: "The single-blind (in which assessor was kept blind), randomised controlled trial (…)"
Measures of at least one key outcome obtained >85% of the participants	Yes	Flow-chart on page 14.
Intention-to-treat	Unclear	Not enough information to make a qualified judgment.
The results of between-group statistical comparisons are reported for at least one key outcome	Yes	Page 15.
The study provides both point measures and measures of variability for at least one key outcome	Yes	Table 1.

Reddy et al. 2015

Type of bias	Judgment	Support for judgment
Eligibility criteria were specified	Yes	Page 56.
Subjects were randomly allocated to groups	Yes	Quote: "The participants were randomly allocated into two groups (…)"
Allocation was concealed	Unclear	Not enough information to make a qualified judgment.
The groups were similar at baseline	Yes	Table 1.
There was blinding of all subjects	No	Not feasible.
There was blinding of all therapists	No	Not feasible.
There was blinding of all assessors – symptoms	No	Not feasible.
There was blinding of all assessors – range of motion	No	Quote: "no blinding of evaluators was done."
Measures of at least one key outcome obtained >85% of the participants	Unclear	Not enough information to make a qualified judgment.
Intention-to-treat	Unclear	Not enough information to make a qualified judgment.
The results of between-group statistical comparisons are reported for at least one key outcome	Yes	Table 2.
The study provides both point measures and measures of variability for at least one key outcome	Yes	Table 2.

Sai and Kumar 2015

Type of bias	Judgment	Support for judgment
Eligibility criteria were specified	Yes	Page 188.
Subjects were randomly allocated to groups	Yes	Quote: "Randomization was done by a random number generator with permuted blocks of 4. The allocation sequence was concealed from the person enrolling the participants, in sequentially numbered, opaque, sealed and stapled envelops."
Allocation was concealed	Yes	Quote: "The allocation sequence was concealed from the person enrolling the participants, in sequentially numbered, opaque, sealed and stapled envelops."
The groups were similar at baseline	Yes	Table 1.
There was blinding of all subjects	No	Not feasible.
There was blinding of all therapists	No	Not feasible.
There was blinding of all assessors – symptoms	No	Not feasible.
There was blinding of all assessors – range of motion	Yes	Quote: "Outcome assessor was kept blinded to the allocation process."
Measures of at least one key outcome obtained >85% of the participants	Yes	Quote: "68 patients accepted the invitation and were randomized. Both groups were well matched at the baseline (Table 1 & 2). Two participants allocated to the experimental group withdrew from the study at six weeks of outcomes measurement after the commencement of the study. In control group two participants withdrew, one at six weeks and one at twelve weeks of outcomes measurement."

Intention-to-treat	Yes	Quote: "All the analyses were done on an intention-to-treat principle and all the randomized subjects were included in the analysis."
The results of between-group statistical comparisons are reported for at least one key outcome	Yes	Table 3.
The study provides both point measures and measures of variability for at least one key outcome	Yes	Table 3.

Shera et al. 2023

Type of bias	Judgment	Support for judgment
Eligibility criteria were specified	Yes	Page 77.
Subjects were randomly allocated to groups	Yes	Quote: "Three groups of frozen shoulder patients were randomly assigned. (…). The computer-generated list used to divide the patients into the three groups (…)"
Allocation was concealed	Unclear	Not enough information to make a qualified judgment.
The groups were similar at baseline	Yes	Page 80.
There was blinding of all subjects	No and unclear	There were three relevant groups, two with active intervention. Blinding of subjects in the active groups was not feasible. Sham mobilization was not mentioned for the third group.
There was blinding of all therapists	No	Not feasible.
There was blinding of all assessors – symptoms	No and unclear	There were three relevant groups, two with active intervention. Blinding of subjects in the active groups was not feasible. Sham mobilization was not mentioned for the third group.
There was blinding of all assessors – range of motion	Unclear	Too little information to make a qualified judgment.
Measures of at least one key outcome obtained >85% of the participants	Yes	According to table 3, all participants were assessed post therapy.
Intention-to-treat	Yes	Not needed.
The results of between-group statistical comparisons are reported for at least one key outcome	Yes	Table 3.
The study provides both point measures and measures of variability for at least one key outcome	Yes	Table 2.

Shrivastava et al. 2011

Type of bias	Judgment	Support for judgment
Eligibility criteria were specified	Yes	Page 12.
Subjects were randomly allocated to groups	Yes	Quote: "A prospective randomized double blind study was performed (…)"
Allocation was concealed	Yes	Quote: "Subjects were randomly allotted to the two groups, Maitland group and Mulligan group by computerised random sequence generator."
The groups were similar at baseline	Yes	Table 1.

There was blinding of all subjects	No	Not feasible.
There was blinding of all therapists	No	Not feasible.
There was blinding of all assessors – symptoms	No	Not feasible.
There was blinding of all assessors – range of motion	Yes	Quote: "Double blinding was done with the assessment therapist and the patient both being blinded with respect to treatment protocol followed"
Measures of at least one key outcome obtained >85% of the participants	Unclear	Not enough information to make a qualified judgment.
Intention-to-treat	Unclear	Not enough information to make a qualified judgment.
The results of between-group statistical comparisons are reported for at least one key outcome	Yes	Table 3.
The study provides both point measures and measures of variability for at least one key outcome	Yes	Table 3.

Yang et al. 2007

Type of bias	Judgment	Support for judgment
Eligibility criteria were specified	Yes	Page 1309.
Subjects were randomly allocated to groups	Yes	Quote: "Consenting subjects were randomly assigned by computer-generated permuted block randomization of 5 by sequentially numbered, sealed, opaque envelopes to receive different mobilization treatments."
Allocation was concealed	Yes	Quote: "Consenting subjects were randomly assigned by computer-generated permuted block randomization of 5 by sequentially numbered, sealed, opaque envelopes to receive different mobilization treatments."
The groups were similar at baseline	Yes	Table 1.
There was blinding of all subjects	No	Not feasible.
There was blinding of all therapists	No	Not feasible.
There was blinding of all assessors – symptoms	No	Not feasible.
There was blinding of all assessors – range of motion	Yes	Quote: "To minimize bias, an independent trained outcome assessor, masked to treatment allocation, evaluated the participants (…)"
Measures of at least one key outcome obtained >85% of the participants	Yes	Figure 1.
Intention-to-treat	Yes	Quote: "(…) we excluded these data to avoid biasing our results. Additionally, similar results were found by including dropout data in the intention-to-treat analysis, which further validates our findings.". The intention-to-treat analysis results were not reported, but the authors mentioned that these results were similar to the results of the completer analysis. We were not sure how to rate this, so we used the score in the PEDro database.

The results of between-group statistical comparisons are reported for at least one key outcome	Yes	Table 3.
The study provides both point measures and measures of variability for at least one key outcome	Yes	Table 3.

Yeole et al. 2017

Type of bias	Judgment	Support for judgment
Eligibility criteria were specified	Yes	Page 3.
Subjects were randomly allocated to groups	Yes	Quote: "Total 30 patients were recruited randomly using simple random sampling technique for the research."
Allocation was concealed	Unclear	Not enough information to make a qualified judgment.
The groups were similar at baseline	Yes	Table 1.
There was blinding of all subjects	No	MWM versus wait-and-see.
There was blinding of all therapists	No	MWM versus wait-and-see.
There was blinding of all assessors – symptoms	No	The patients were not blinded, and they scored their own pain.
There was blinding of all assessors – range of motion	Unclear	Not enough information to make a qualified judgment.
Measures of at least one key outcome obtained >85% of the participants	Unclear	Not enough information to make a qualified judgment.
Intention-to-treat	Unclear	Not enough information to make a qualified judgment.
The results of between-group statistical comparisons are reported for at least one key outcome	Yes	Page 4.
The study provides both point measures and measures of variability for at least one key outcome	Yes	Table 1.

Youssef et al. 2015

Type of bias	Judgment	Support for judgment
Eligibility criteria were specified	Yes	Table 1.
Subjects were randomly allocated to groups	Yes	Quote: "Patients were randomly assigned using sealed envelopes (...)"
Allocation was concealed	Yes	Quote: "Patients were randomly assigned using sealed envelopes (...)"
The groups were similar at baseline	Yes	Figures 1-3.
There was blinding of all subjects	No	Not feasible.
There was blinding of all therapists	No	Not feasible.
There was blinding of all assessors – symptoms	-	Symptoms not assessed.
There was blinding of all assessors – range of motion	Unclear	Not enough information to make a qualified judgment.
Measures of at least one key outcome obtained >85% of the participants	Unclear	Not enough information to make a qualified judgment.
Intention-to-treat	Unclear	Not enough information to make a qualified judgment.
The results of between-group statistical comparisons are reported for at least one key outcome	Yes	Table 2.

The study provides both point measures and measures of variability for at least one key outcome	Yes	Table 2.

Zaghloul et al. 2022

Type of bias	Judgment	Support for judgment
Eligibility criteria were specified	Yes	Pages 3251-3252.
Subjects were randomly allocated to groups	Yes	Quote: "In this study, 36 patients with a diagnosis of adhesive capsulitis (stage II) were randomly divided into two groups."
Allocation was concealed	Unclear	Not enough information to make a qualified judgment.
The groups were similar at baseline	Yes	Table 1.
There was blinding of all subjects	No	Not feasible.
There was blinding of all therapists	No	Not feasible.
There was blinding of all assessors – symptoms	No	Not feasible.
There was blinding of all assessors – range of motion	Unclear	Not enough information to make a qualified judgment.
Measures of at least one key outcome obtained >85% of the participants	Yes	Quote: "We examined forty-three patients, only thirty-six patients completed the study". It is likely that all participants that completed the study were reassessed post therapy.
Intention-to-treat	Unclear	Not enough information to make a qualified judgment.
The results of between-group statistical comparisons are reported for at least one key outcome	Yes	Quote: "(…) patients who got mobilization with movement had substantially less pain than those who got scapular mobilization, with a mean difference of 1.184 ± 0.410 on a ten-point classical VAS (p-value = 0.001, 95% CI = -0.539–1.854)."
The study provides both point measures and measures of variability for at least one key outcome	Yes	Quote: "(…) patients who got mobilization with movement had substantially less pain than those who got scapular mobilization, with a mean difference of 1.184 ± 0.410 on a ten-point classical VAS (p-value = 0.001, 95% CI = -0.539–1.854)."

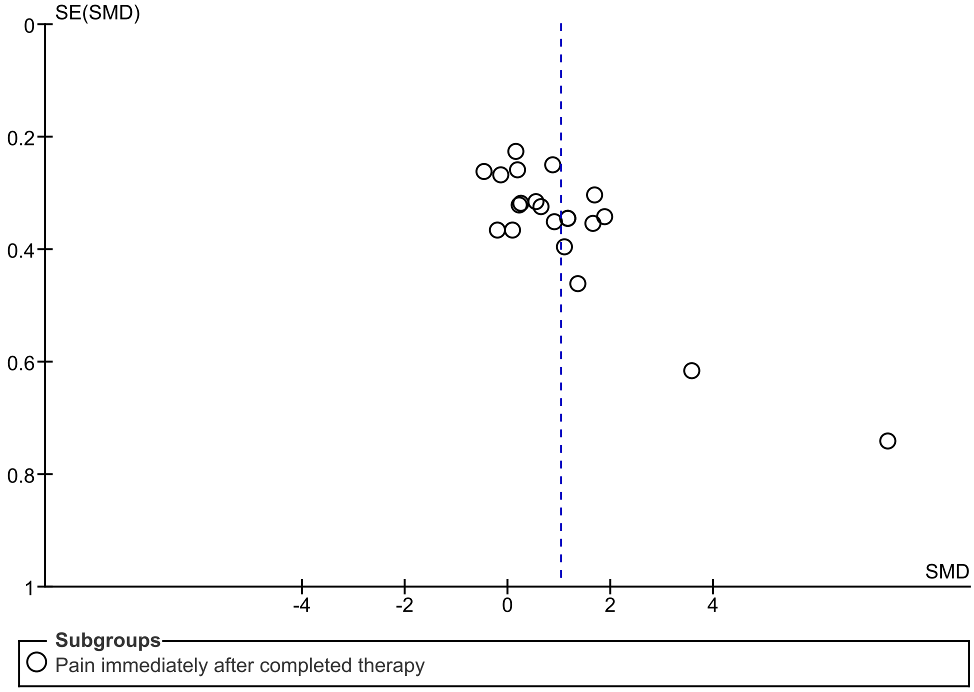

Figure S1 Funnel plot of AC pain results immediately post-therapy – MWM versus other types of mobilization.
AC = adhesive capsulitis; MWM = Mobilization With Movement.

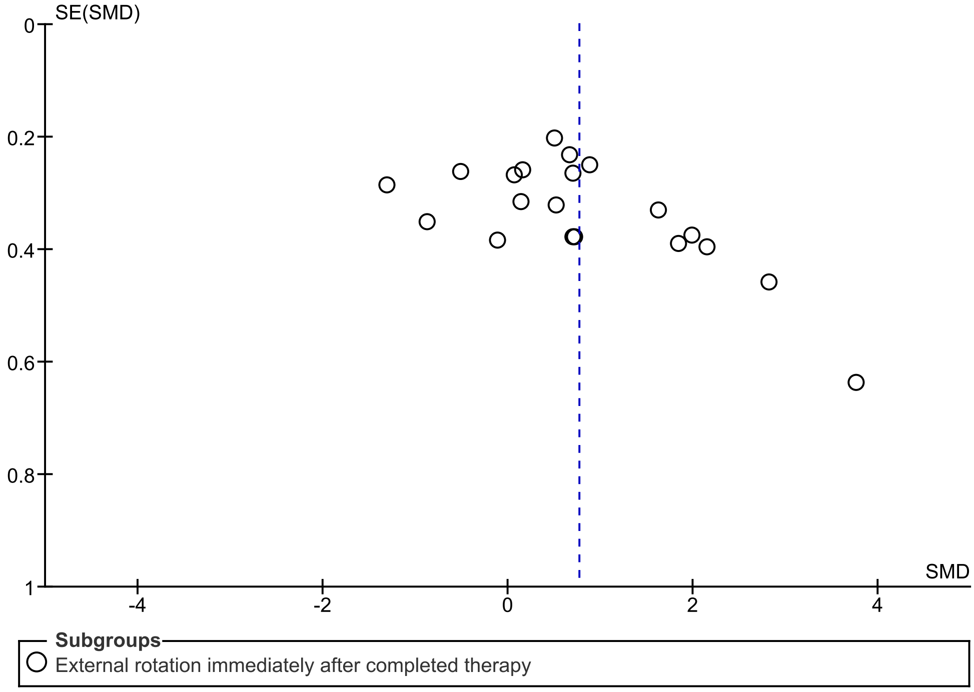

Figure S2 Funnel plot of AC external rotation ROM results immediately post-therapy – MWM versus other types of mobilization.
AC = adhesive capsulitis; MWM = Mobilization With Movement; ROM = range of motion.

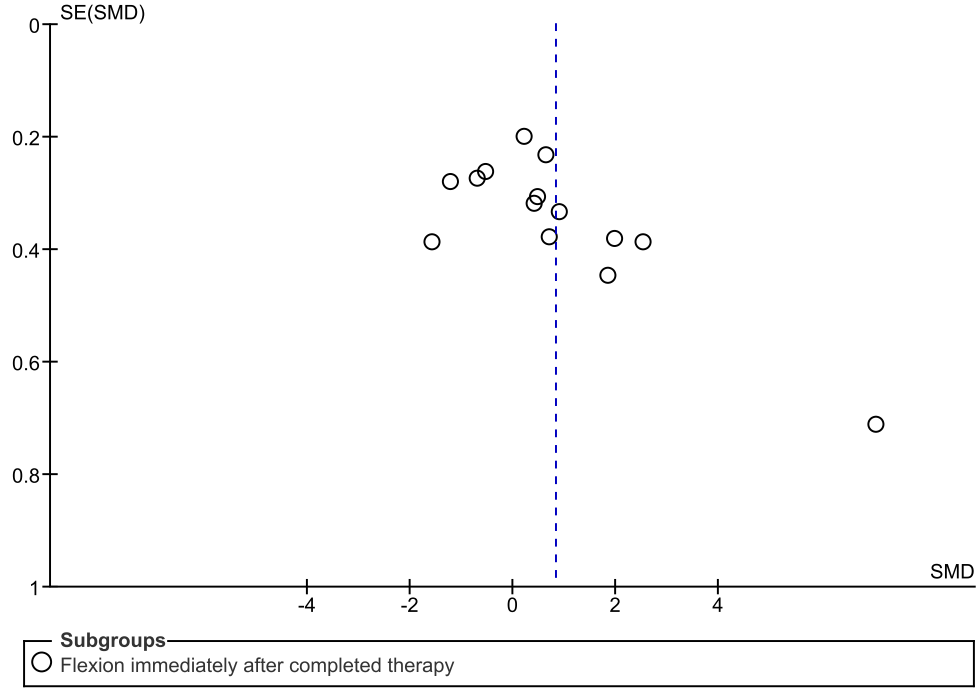

Figure S3 Funnel plot of AC flexion ROM results immediately post-therapy – MWM versus other types of mobilization.
AC = adhesive capsulitis; MWM = Mobilization With Movement; ROM = range of motion.

	MWM			Control				Std. Mean Difference
**Study or Subgroup**	**Mean**	**SD**	**Total**	**Mean**	**SD**	**Total**	**Weight**	**IV, Random, 95% CI**
Sai 2015 versus no intervention	4.09	4.708	34	1.8	4.708	34	19.3%	0.48 [-0.00, 0.96]
Reddy 2015 versus no intervention	5.76	1.31	15	4.58	1.31	15	17.2%	0.88 [0.12, 1.63]
Yeole 2017 versus no intervention	4.53	3.851	15	1.66	1.429	15	17.2%	0.96 [0.20, 1.72]
Jeyakumar 2018 versus no intervention	2.315	1.486	17	0	1.486	35	18.0%	1.53 [0.88, 2.19]
Shera 2023 versus no intervention	4.09	0.933	12	1.83	0.99	12	14.6%	2.27 [1.20, 3.33]
Ranjana 2016 versus no intervention	4.32	0.592	15	1.86	0.78	15	13.7%	3.46 [2.28, 4.64]
**Total (95% CI)**			**108**			**126**	**100.0%**	**1.49 [0.75, 2.23]**

Heterogeneity: Tau² = 0.67; Chi² = 28.36, df = 5 (P < 0.0001); I² = 82%
Test for overall effect: Z = 3.96 (P < 0.0001)

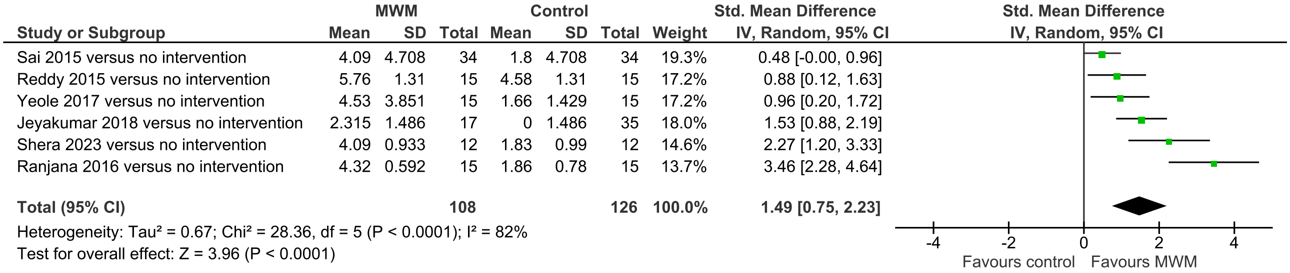

Figure S4 Forest plot of AC pain results immediately post-therapy – MWM versus no-intervention control.
AC = adhesive capsulitis; MWM = Mobilization With Movement.

	MWM			Control				Std. Mean Difference
**Study or Subgroup**	**Mean**	**SD**	**Total**	**Mean**	**SD**	**Total**	**Weight**	**IV, Random, 95% CI**
**11.1.1 Disability immediately after completed therapy**								
Sai 2015 versus no intervention	24.4	26.52	34	11.5	26.52	34	54.2%	0.48 [-0.00, 0.96]
Reddy 2015 versus no intervention	55.8	16.729	15	47.4	16.729	15	23.8%	0.49 [-0.24, 1.22]
Yeole 2017 versus no intervention	34.56	32.327	15	11.87	10.194	15	22.0%	0.92 [0.16, 1.68]
**Subtotal (95% CI)**			**64**			**64**	**100.0%**	**0.58 [0.22, 0.93]**

Heterogeneity: Tau² = 0.00; Chi² = 1.00, df = 2 (P = 0.61); I² = 0%
Test for overall effect: Z = 3.20 (P = 0.001)

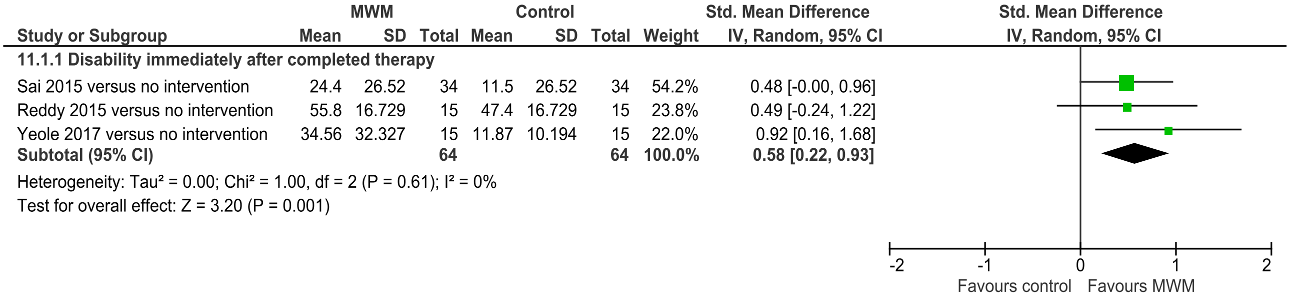

Figure S5 Forest plot of AC disability results immediately post-therapy – MWM versus no-intervention control.
AC = adhesive capsulitis; MWM = Mobilization With Movement.

Study or Subgroup	MWM Mean	MWM SD	MWM Total	Control Mean	Control SD	Control Total	Weight	Std. Mean Difference IV, Random, 95% CI
Reddy 2015 versus no intervention	28.74	10.251	15	21.94	10.251	15	18.2%	0.65 [-0.09, 1.38]
Yeole 2017 versus no intervention	18.87	16.206	15	6.17	10.914	15	17.7%	0.89 [0.14, 1.65]
Jeyakumar 2018 versus no intervention	14.715	10.71	35	0	10.71	35	25.5%	1.36 [0.84, 1.88]
Sai 2015 versus no intervention	19.8	5.906	34	10.3	6.085	34	24.5%	1.57 [1.02, 2.11]
Ranjana 2016 versus no intervention	34.87	10.946	15	7.66	14.682	15	14.1%	2.04 [1.14, 2.95]
**Total (95% CI)**			**114**			**114**	**100.0%**	**1.29 [0.88, 1.71]**

Heterogeneity: Tau² = 0.11; Chi² = 7.69, df = 4 (P = 0.10); I² = 48%
Test for overall effect: Z = 6.10 (P < 0.00001)

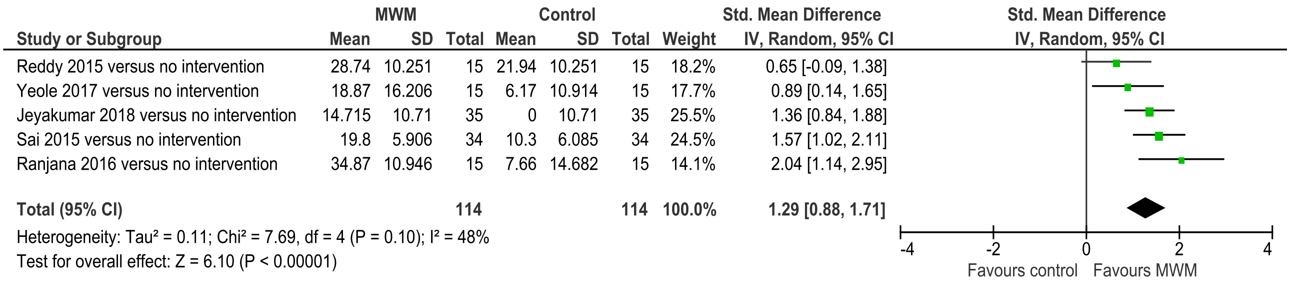

Figure S6 Forest plot of AC external rotation ROM results immediately post-therapy – MWM versus no-intervention control.
AC = adhesive capsulitis; MWM = Mobilization With Movement; ROM = range of motion.

Study or Subgroup	MWM Mean	MWM SD	MWM Total	Control Mean	Control SD	Control Total	Weight	Std. Mean Difference IV, Random, 95% CI
Yeole 2017 versus no intervention	31.2	29.184	15	12.66	13.686	15	24.9%	0.79 [0.04, 1.54]
Reddy 2015 versus no intervention	61.2	15.719	15	45.34	15.719	15	23.9%	0.98 [0.22, 1.75]
Sai 2015 versus no intervention	38.4	9.351	34	25.6	11.017	34	51.2%	1.24 [0.72, 1.76]
**Total (95% CI)**			**64**			**64**	**100.0%**	**1.07 [0.69, 1.44]**

Heterogeneity: Tau² = 0.00; Chi² = 0.99, df = 2 (P = 0.61); I² = 0%
Test for overall effect: Z = 5.60 (P < 0.00001)

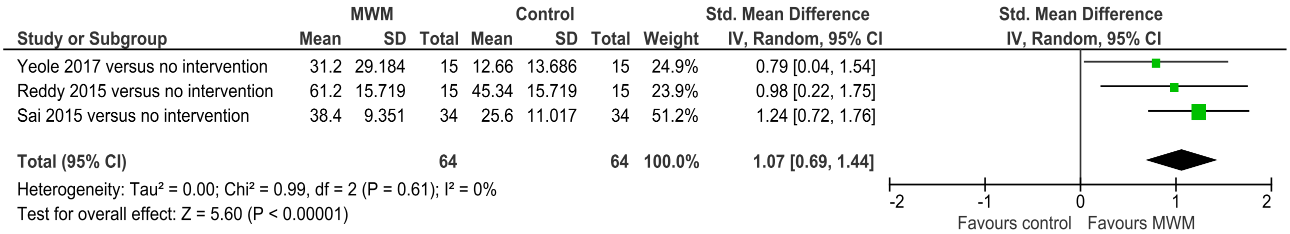

Figure S7 Forest plot of AC flexion ROM results immediately post-therapy – MWM versus no-intervention control.
AC = adhesive capsulitis; MWM = Mobilization With Movement; ROM = range of motion.

Study or Subgroup	MWM Mean	MWM SD	MWM Total	Control Mean	Control SD	Control Total	Weight	Std. Mean Difference IV, Random, 95% CI
Guimarães 2016 versus placebo	10.4	18.012	14	11.5	19.113	13	40.1%	-0.06 [-0.81, 0.70]
Delgado-Gil 2015 versus placebo	0.6	1.757	21	-0.2	1.757	21	59.9%	0.45 [-0.17, 1.06]
**Total (95% CI)**			**35**			**34**	**100.0%**	**0.24 [-0.24, 0.73]**

Heterogeneity: Tau² = 0.00; Chi² = 1.03, df = 1 (P = 0.31); I² = 3%
Test for overall effect: Z = 0.99 (P = 0.32)

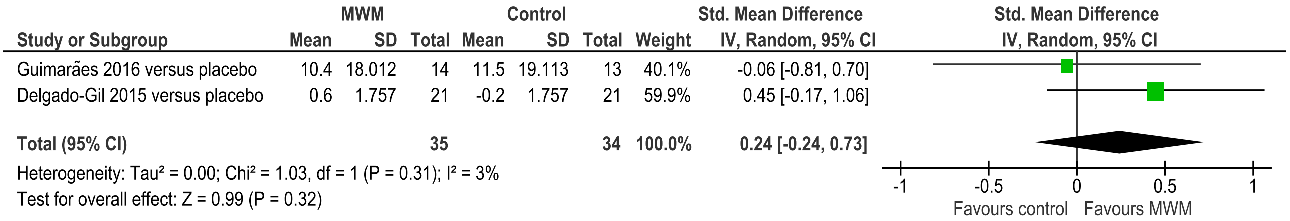

Figure S8 Forest plot of SAPS pain results immediately post-therapy – MWM versus sham mobilization.
MWM = Mobilization With Movement; SAPS = subacromial pain syndrome.

Study or Subgroup	MWM Mean	MWM SD	MWM Total	Control Mean	Control SD	Control Total	Weight	Std. Mean Difference IV, Random, 95% CI
Ishaq 2023 versus no intervention	3.65	0.993	20	3.45	0.874	20	26.9%	0.21 [-0.41, 0.83]
Kachingwe 2013 versus no intervention	55.2	31.9	9	20.8	112.3	8	22.1%	0.41 [-0.56, 1.37]
Dalvi 2024 versus no intervention	3.43	0.809	40	2.7	0.648	40	28.8%	0.99 [0.52, 1.45]
Neelapala 2016 verus no intervention	3.38	0.849	15	1	1.006	16	22.1%	2.48 [1.52, 3.45]
**Total (95% CI)**			**84**			**84**	**100.0%**	**0.98 [0.15, 1.81]**

Heterogeneity: Tau² = 0.56; Chi² = 16.16, df = 3 (P = 0.001); I² = 81%
Test for overall effect: Z = 2.32 (P = 0.02)

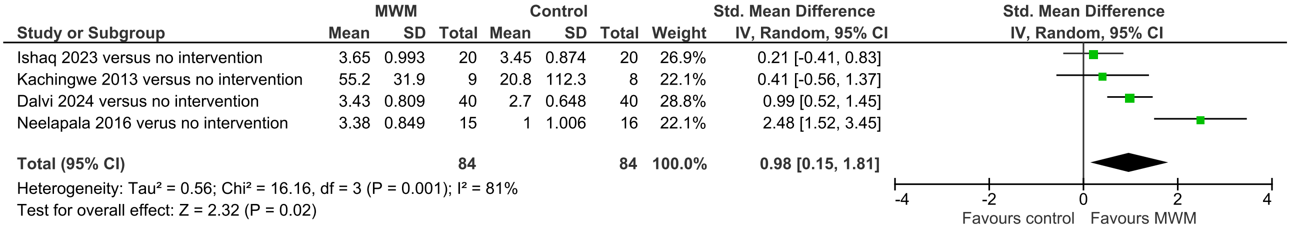

Figure S9 Forest plot of SAPS pain results immediately post-therapy – MWM versus no-intervention control.
MWM = Mobilization With Movement; SAPS = subacromial pain syndrome.

Study or Subgroup	MWM Mean	MWM SD	MWM Total	Control Mean	Control SD	Control Total	Weight	Std. Mean Difference IV, Random, 95% CI
Guimarães 2016 versus placebo	10.5	24.247	14	6.1	24.078	13	45.6%	0.18 [-0.58, 0.93]
Delgado-Gil 2015 versus placebo	6.8	9.117	21	-1.4	9.117	21	54.4%	0.88 [0.25, 1.52]
**Total (95% CI)**			**35**			**34**	**100.0%**	**0.56 [-0.13, 1.25]**

Heterogeneity: Tau² = 0.12; Chi² = 1.96, df = 1 (P = 0.16); I² = 49%
Test for overall effect: Z = 1.59 (P = 0.11)

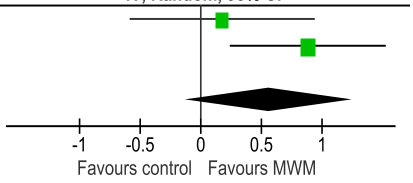

Figure S10 Forest plot of SAPS external rotation ROM results immediately post-therapy – MWM versus sham mobilization.
MWM = Mobilization With Movement; ROM = range of motion; SAPS = subacromial pain syndrome.

Study or Subgroup	MWM Mean	MWM SD	MWM Total	Control Mean	Control SD	Control Total	Weight	Std. Mean Difference IV, Random, 95% CI
Ishaq 2023 versus no intervention	15.67	8.124	20	17.89	10.979	20	33.2%	-0.23 [-0.85, 0.40]
Dalvi 2024 versus no intervention	10.7	6.107	40	10.8	8.031	40	66.8%	-0.01 [-0.45, 0.42]
**Total (95% CI)**			**60**			**60**	**100.0%**	**-0.08 [-0.44, 0.27]**

Heterogeneity: Tau² = 0.00; Chi² = 0.30, df = 1 (P = 0.59); I² = 0%
Test for overall effect: Z = 0.46 (P = 0.65)

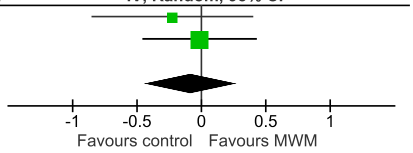

Figure S11 Forest plot of SAPS external rotation ROM results immediately post-therapy – MWM versus no-intervention control.
MWM = Mobilization With Movement; ROM = range of motion; SAPS = subacromial pain syndrome.

Study or Subgroup	MWM Mean	MWM SD	MWM Total	Control Mean	Control SD	Control Total	Weight	Std. Mean Difference IV, Random, 95% CI
Guimarães 2016 versus placebo	3.3	19.744	14	-1.6	19.61	13	49.7%	0.24 [-0.52, 1.00]
Delgado-Gil 2015 versus placebo	31	18.783	21	-3.2	18.783	21	50.3%	1.79 [1.06, 2.51]
**Total (95% CI)**			**35**			**34**	**100.0%**	**1.02 [-0.50, 2.53]**

Heterogeneity: Tau² = 1.05; Chi² = 8.32, df = 1 (P = 0.004); I² = 88%
Test for overall effect: Z = 1.32 (P = 0.19)

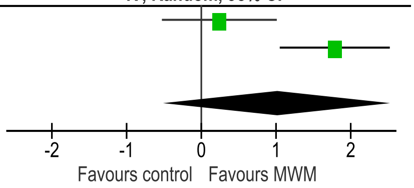

Figure S12 Forest plot of SAPS flexion ROM results immediately post-therapy – MWM versus sham mobilization.
MWM = Mobilization With Movement; ROM = range of motion; SAPS = subacromial pain syndrome.

Study or Subgroup	MWM Mean	MWM SD	MWM Total	Control Mean	Control SD	Control Total	Weight	Std. Mean Difference IV, Random, 95% CI
Ishaq 2023 versus no intervention	32.45	15.979	20	26.72	18	20	29.6%	0.33 [-0.29, 0.95]
Kachingwe 2013 versus no intervention	46.7	31.9	9	27.6	41.7	8	12.2%	0.49 [-0.48, 1.46]
Dalvi 2024 versus no intervention	18.3	10.787	40	12.83	10.984	40	58.2%	0.50 [0.05, 0.94]
**Total (95% CI)**			**69**			**68**	**100.0%**	**0.45 [0.11, 0.79]**

Heterogeneity: Tau² = 0.00; Chi² = 0.19, df = 2 (P = 0.91); I² = 0%
Test for overall effect: Z = 2.58 (P = 0.010)

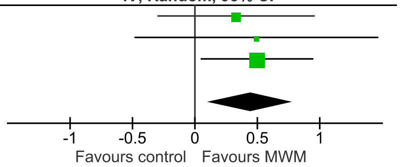

Figure S13 Forest plot of SAPS flexion ROM results immediately post-therapy – MWM versus no-intervention control.
MWM = Mobilization With Movement; ROM = range of motion; SAPS = subacromial pain syndrome.

Study or Subgroup	MWM Mean	MWM SD	MWM Total	Control Mean	Control SD	Control Total	Weight	Std. Mean Difference IV, Random, 95% CI
**17.1.1 Pain immediately after completed therapy - PEDro score ≥5**								
Jung 2020 versus continuous passive motion	27.2	7.54	15	28.67	8.3	15	4.8%	-0.18 [-0.90, 0.54]
Khalil 2022 versus Muscle Energy Technique	4.08	0.5	39	4	0.547	39	5.2%	0.15 [-0.29, 0.60]
Rayudu 2019 versus Muscle Energy Technique	11.93	3.574	30	11.23	3.068	30	5.1%	0.21 [-0.30, 0.71]
Azam 2024 verus Maitland mobilization	4.1052	0.618	19	3.95	0.781	20	4.9%	0.22 [-0.41, 0.85]
Khyathi 2015 versus Spencer mobilization	3.34	3.385	20	2.72	0.964	20	4.9%	0.24 [-0.38, 0.87]
Dilip 2016 versus Gong's mobilization	2.47	0.454	20	2.07	0.702	20	4.9%	0.66 [0.02, 1.30]
Jeyakumar 2018 versus Maitland mobilization	1.315	1.486	35	0	1.486	35	5.1%	0.88 [0.38, 1.37]
Zaghloul 2022 versus scapular mobilization	5.72	1.107	18	4.73	1.036	18	4.8%	0.90 [0.21, 1.59]
Doner 2013 versus stretching	5.15	1.743	20	3.36	1.165	20	4.8%	1.18 [0.51, 1.86]
Shera 2023 versus Spencer mobilization	4.09	0.933	12	2.75	0.972	12	4.4%	1.36 [0.45, 2.26]
Patil 2010 versus Kaltenborn mobilization	4.7	0.93	30	3.2	0.81	30	5.0%	1.70 [1.10, 2.29]
Boruah 2015 versus scapular mobilization	20.48	5.221	25	10.64	5.074	25	4.8%	1.88 [1.21, 2.56]
Razzaq 2022 versus Muscle Energy Technique	5.41	0.49	32	2.07	0.39	29	3.4%	7.40 [5.96, 8.85]
**Subtotal (95% CI)**			**315**			**313**	**62.1%**	**1.13 [0.56, 1.71]**
Heterogeneity: Tau² = 0.99; Chi² = 128.43, df = 12 (P < 0.00001); I² = 91%								
Test for overall effect: Z = 3.85 (P = 0.0001)								
**17.1.2 Pain immediately after completed therapy - PEDro score ≤4**								
Mehta 2018 versus passive stretching	30.6	38.604	30	49	38.604	30	5.1%	-0.47 [-0.98, 0.04]
Fernandes 2020 versus Kaltenborn mobilization	22.07	56.649	28	31.56	81.008	28	5.1%	-0.13 [-0.66, 0.39]
Pankaj 2013 versus Gong's mobilization	2.03	0.641	15	1.97	0.686	15	4.8%	0.09 [-0.63, 0.80]
Patel 2022 versus Muscle Energy Technique	2.66	1.11	21	2.09	0.88	21	4.9%	0.56 [-0.06, 1.18]
Ranjana 2016 versus Maitland mobilization	4.32	0.592	15	3.76	0.369	15	4.7%	1.10 [0.33, 1.88]
Rathod 2019 verus Kaltenborn mobilization	4	0.858	20	3	0.795	20	4.8%	1.19 [0.51, 1.86]
Khan 2023 versus Sleeper stretch	4.87	0.952	22	3.091	1.148	22	4.8%	1.66 [0.96, 2.35]
Nithya 2021 versus Muscle Energy Technique	4.07	0.676	15	1.94	0.465	15	3.9%	3.57 [2.37, 4.78]
**Subtotal (95% CI)**			**166**			**166**	**37.9%**	**0.86 [0.15, 1.57]**
Heterogeneity: Tau² = 0.91; Chi² = 62.51, df = 7 (P < 0.00001); I² = 89%								
Test for overall effect: Z = 2.38 (P = 0.02)								
**Total (95% CI)**			**481**			**479**	**100.0%**	**1.03 [0.59, 1.46]**
Heterogeneity: Tau² = 0.92; Chi² = 194.07, df = 20 (P < 0.00001); I² = 90%								
Test for overall effect: Z = 4.58 (P < 0.00001)								
Test for subgroup differences: Chi² = 0.34, df = 1 (P = 0.56), I² = 0%								

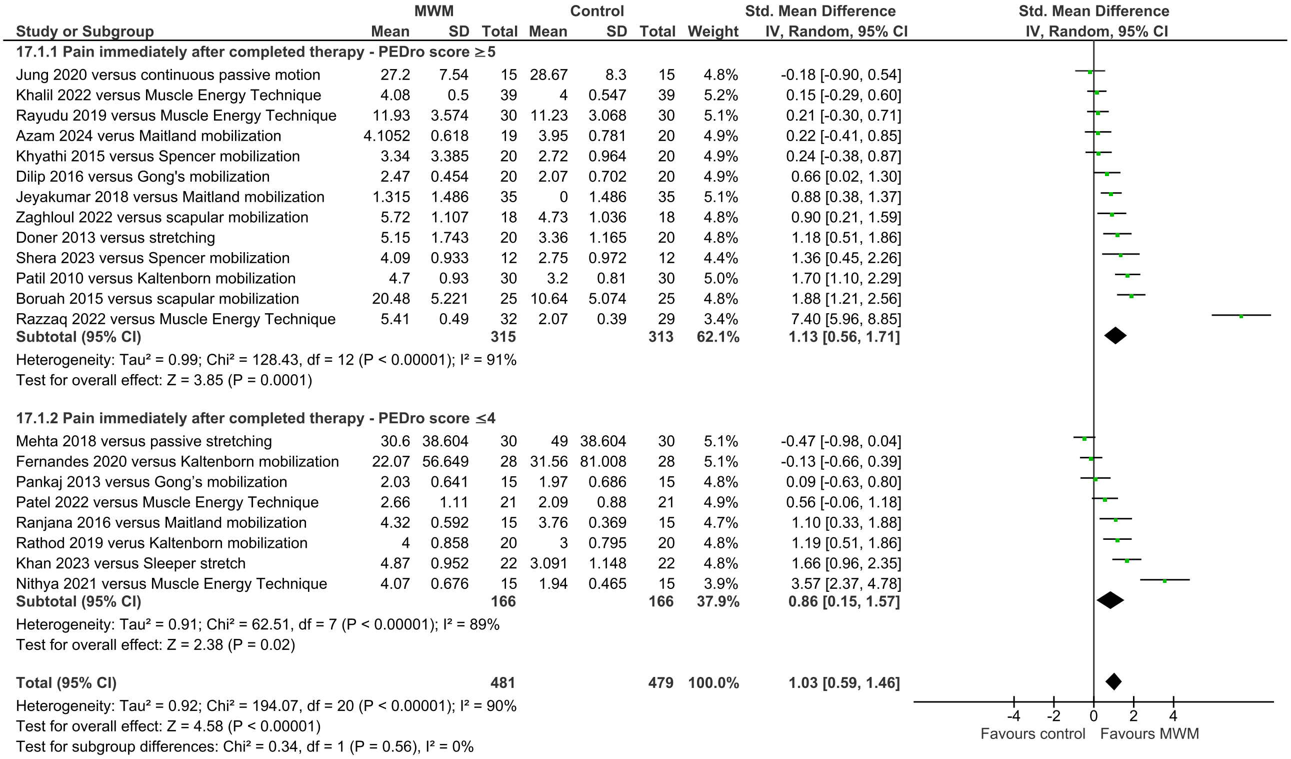

Figure S14 Forest plot of AC pain results immediately post-therapy – MWM versus other types of mobilization – subgrouped by risk of bias.
AC = adhesive capsulitis; MWM = Mobilization With Movement.

Study or Subgroup	MWM Mean	MWM SD	MWM Total	Control Mean	Control SD	Control Total	Weight	Std. Mean Difference IV, Random, 95% CI
**18.1.1 Disability immediately after completed therapy - PEDro score ≥5**								
Rayudu 2019 versus Muscle Energy Technique	16.84	5.584	30	14.07	5.645	30	14.9%	0.49 [-0.03, 1.00]
Yang 2007 versus Maitland end-range mobilization	9	4.061	13	5.2	3.774	14	13.2%	0.94 [0.14, 1.74]
Doner 2013 versus stretching	57.23	21.732	20	34.68	24.539	20	14.1%	0.95 [0.30, 1.61]
Khalil 2022 versus Muscle Energy Technique	68.94	15.665	39	47.93	7.049	39	14.9%	1.71 [1.19, 2.24]
Khyathi 2015 versus Spencer mobilization	23.98	3.012	20	15.87	4.198	20	13.2%	2.18 [1.38, 2.97]
**Subtotal (95% CI)**			**122**			**123**	**70.2%**	**1.24 [0.63, 1.84]**
Heterogeneity: Tau² = 0.36; Chi² = 17.75, df = 4 (P = 0.001); I² = 77%								
Test for overall effect: Z = 4.01 (P < 0.0001)								
**18.1.2 Disability immediately after completed therapy - PEDro score ≤4**								
Mehta 2018 versus passive stretching	29.2	76.375	30	42.9	76.375	30	14.9%	-0.18 [-0.68, 0.33]
Fernandes 2020 versus Kaltenborn mobilization	20.44	52.465	28	29.86	76.644	28	14.9%	-0.14 [-0.67, 0.38]
**Subtotal (95% CI)**			**58**			**58**	**29.8%**	**-0.16 [-0.52, 0.20]**
Heterogeneity: Tau² = 0.00; Chi² = 0.01, df = 1 (P = 0.92); I² = 0%								
Test for overall effect: Z = 0.86 (P = 0.39)								
**Total (95% CI)**			**180**			**181**	**100.0%**	**0.82 [0.17, 1.48]**
Heterogeneity: Tau² = 0.68; Chi² = 50.51, df = 6 (P < 0.00001); I² = 88%								
Test for overall effect: Z = 2.48 (P = 0.01)								
Test for subgroup differences: Chi² = 15.05, df = 1 (P = 0.0001), I² = 93.4%								

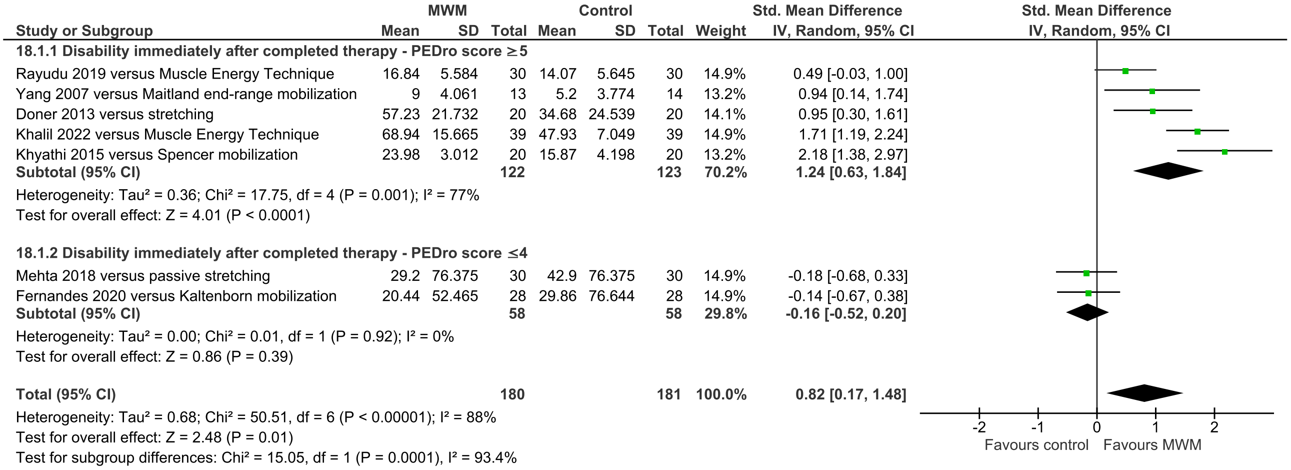

Figure S15 Forest plot of AC disability results immediately post-therapy – MWM versus other types of mobilization – subgrouped by risk of bias.
AC = adhesive capsulitis; MWM = Mobilization With Movement.

Study or Subgroup	MWM Mean	MWM SD	MWM Total	Control Mean	Control SD	Control Total	Weight	Std. Mean Difference IV, Random, 95% CI
**19.1.1 External rotation immediately after completed therapy - PEDro score ≥5**								
Patil 2010 versus Kaltenborn mobilization	26.3	7.28	30	34.5	5.02	30	5.2%	-1.29 [-1.85, -0.73]
Zaghloul 2022 versus scapular mobilization	23.36	5.005	18	27.86	5.205	18	5.0%	-0.86 [-1.55, -0.18]
Yang 2007 versus Maitland end-range mobilization	34.2	14.3	13	36.4	24.3	14	4.8%	-0.11 [-0.86, 0.65]
Rayudu 2019 versus Muscle Energy Technique	14.66	7.267	30	13.5	6.719	30	5.3%	0.16 [-0.34, 0.67]
Arshad 2015 versus Maitland mobilization	43.84	7.185	50	39.62	9.403	50	5.4%	0.50 [0.10, 0.90]
Doner 2013 versus stretching	30.5	19.084	20	20.25	19.867	20	5.1%	0.52 [-0.12, 1.15]
Khalil 2022 versus Muscle Energy Technique	14.15	7.841	39	8.54	8.76	39	5.3%	0.67 [0.21, 1.12]
Minerva 2016 versus Maitland mobilization	16.67	7.161	30	11.5	7.161	30	5.2%	0.71 [0.19, 1.24]
Youssef 2015 versus Maitland mobilization	59.29	14.637	15	48.29	14.637	15	4.9%	0.73 [-0.01, 1.47]
Jeyakumar 2018 versus Maitland mobilization	9.571	10.71	35	0	10.71	35	5.3%	0.88 [0.39, 1.38]
Boruah 2015 versus scapular mobilization	21.2	6.32	25	11.44	5.391	25	5.0%	1.64 [0.99, 2.28]
Azam 2024 verus Maitland mobilization	33.8421	2.637	19	28.2	3.285	20	4.8%	1.85 [1.09, 2.61]
Patel 2022 versus Muscle Energy Technique	34.9	7.68	21	21.76	3.63	21	4.8%	2.15 [1.37, 2.92]
Khyathi 2015 versus Spencer mobilization	19.8	3.205	20	10.95	2.946	20	4.6%	2.82 [1.92, 3.72]
**Subtotal (95% CI)**			**365**			**367**	**70.8%**	**0.71 [0.21, 1.21]**
Heterogeneity: Tau² = 0.80; Chi² = 128.41, df = 13 (P < 0.00001); I² = 90%								
Test for overall effect: Z = 2.80 (P = 0.005)								
**19.1.2 External rotation immediately after completed therapy - PEDro score ≤4**								
Mehta 2018 versus passive stretching	3.8	15.023	30	11.6	15.023	30	5.3%	-0.51 [-1.03, 0.00]
Fernandes 2020 versus Kaltenborn mobilization	14.21	13.109	28	12.86	19.515	28	5.2%	0.08 [-0.44, 0.60]
Rathod 2019 verus Kaltenborn mobilization	20.3	4.669	20	19.5	6.621	20	5.1%	0.14 [-0.48, 0.76]
Ranjana 2016 versus Maitland mobilization	34.87	10.946	15	25.74	14.246	15	4.9%	0.70 [-0.04, 1.44]
Khan 2023 versus Sleeper stretch	30.46	5.388	22	18.63	6.292	22	4.9%	1.98 [1.25, 2.72]
Nithya 2021 versus Muscle Energy Technique	40.53	3.348	15	28.67	2.776	15	3.9%	3.75 [2.51, 5.00]
**Subtotal (95% CI)**			**130**			**130**	**29.2%**	**0.92 [-0.02, 1.87]**
Heterogeneity: Tau² = 1.26; Chi² = 60.54, df = 5 (P < 0.00001); I² = 92%								
Test for overall effect: Z = 1.91 (P = 0.06)								
**Total (95% CI)**			**495**			**497**	**100.0%**	**0.77 [0.33, 1.20]**
Heterogeneity: Tau² = 0.85; Chi² = 189.93, df = 19 (P < 0.00001); I² = 90%								
Test for overall effect: Z = 3.48 (P = 0.0005)								
Test for subgroup differences: Chi² = 0.15, df = 1 (P = 0.70), I² = 0%								

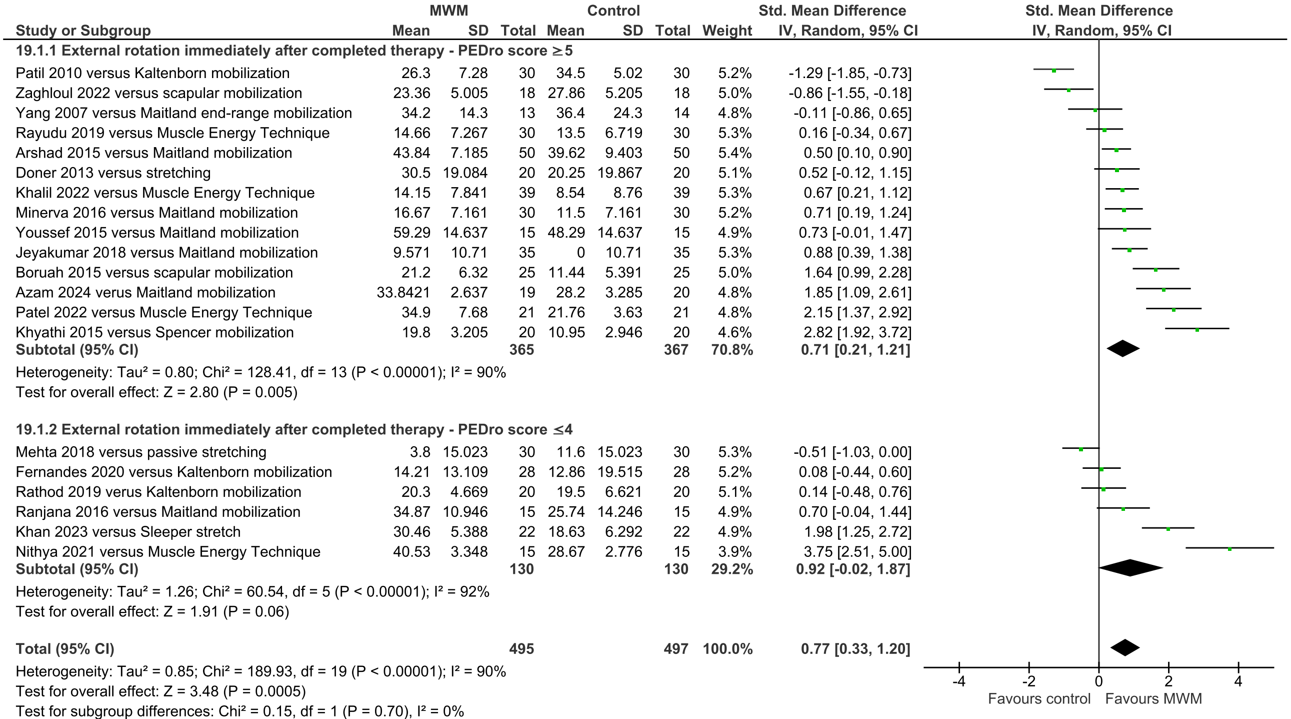

Figure S16 Forest plot of AC external rotation ROM results immediately post-therapy – MWM versus other types of mobilization – subgrouped by risk of bias.
AC = adhesive capsulitis; MWM = Mobilization With Movement; ROM = range of motion.

Study or Subgroup	MWM Mean	MWM SD	MWM Total	Control Mean	Control SD	Control Total	Weight	Std. Mean Difference IV, Random, 95% CI
**20.1.1 Flexion immediately after completed therapy - PEDro score ≥5**								
Zaghloul 2022 versus scapular mobilization	15.07	5.6	18	23.93	5.39	18	7.1%	-1.58 [-2.33, -0.82]
Patil 2010 versus Kaltenborn mobilization	44	8.84	30	52.3	3.97	30	7.4%	-1.20 [-1.75, -0.64]
Arshad 2015 versus Maitland mobilization	105.68	14.089	50	102.12	15.425	50	7.5%	0.24 [-0.15, 0.63]
Doner 2013 versus stretching	38	14.547	20	31.25	15.996	20	7.3%	0.43 [-0.20, 1.06]
Khalil 2022 versus Muscle Energy Technique	28.72	17.615	39	15.82	20.762	39	7.5%	0.66 [0.21, 1.12]
Youssef 2015 versus Maitland mobilization	91.54	33.214	15	66.58	33.214	15	7.1%	0.73 [-0.01, 1.47]
Jung 2020 versus continuous passive motion	37.67	4.95	15	29.33	3.72	15	6.9%	1.85 [0.98, 2.73]
Patel 2022 versus Muscle Energy Technique	67.76	11.92	21	46.66	8.87	21	7.1%	1.97 [1.22, 2.72]
Boruah 2015 versus scapular mobilization	45.96	8.217	25	26.08	7.198	25	7.1%	2.53 [1.78, 3.29]
Razzaq 2022 versus Muscle Energy Technique	90.75	8.99	32	31.8	7.29	29	5.9%	7.07 [5.68, 8.47]
**Subtotal (95% CI)**			**265**			**262**	**70.7%**	**1.18 [0.21, 2.15]**
Heterogeneity: Tau² = 2.29; Chi² = 204.54, df = 9 (P < 0.00001); I² = 96%								
Test for overall effect: Z = 2.39 (P = 0.02)								
**20.1.2 Flexion immediately after completed therapy - PEDro score ≤4**								
Fernandes 2020 versus Kaltenborn mobilization	21.81	12.26	28	28.68	7.23	28	7.4%	-0.67 [-1.21, -0.13]
Mehta 2018 versus passive stretching	7.1	15.601	30	15.2	15.601	30	7.4%	-0.51 [-1.03, 0.00]
Khan 2023 versus Sleeper stretch	62.27	8.29	22	56.36	14.37	22	7.3%	0.49 [-0.11, 1.10]
Rathod 2019 verus Kaltenborn mobilization	36.35	9.778	20	27.9	8.404	20	7.2%	0.91 [0.25, 1.56]
**Subtotal (95% CI)**			**100**			**100**	**29.3%**	**0.04 [-0.70, 0.77]**
Heterogeneity: Tau² = 0.47; Chi² = 19.62, df = 3 (P = 0.0002); I² = 85%								
Test for overall effect: Z = 0.10 (P = 0.92)								
**Total (95% CI)**			**365**			**362**	**100.0%**	**0.83 [0.13, 1.53]**
Heterogeneity: Tau² = 1.67; Chi² = 236.45, df = 13 (P < 0.00001); I² = 95%								
Test for overall effect: Z = 2.31 (P = 0.02)								
Test for subgroup differences: Chi² = 3.42, df = 1 (P = 0.06), I² = 70.7%								

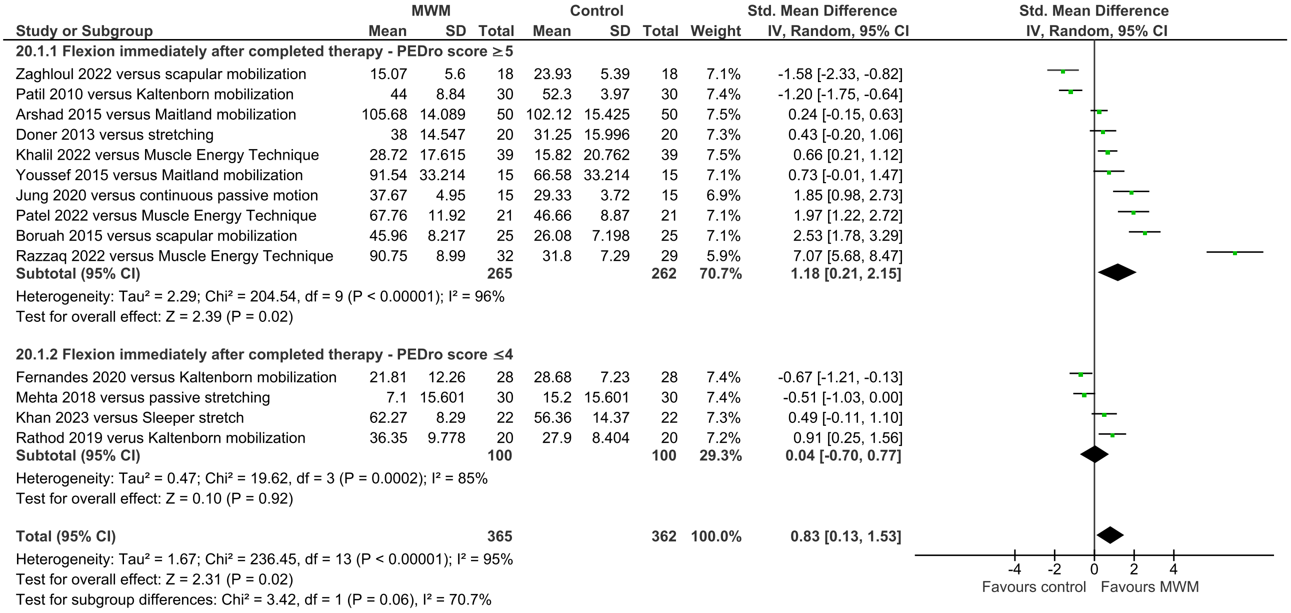

Figure S17 Forest plot of AC flexion ROM results immediately post-therapy – MWM versus other types of mobilization – subgrouped by risk of bias.
AC = adhesive capsulitis; MWM = Mobilization With Movement; ROM = range of motion.

Study or Subgroup	MWM Mean	MWM SD	MWM Total	Control Mean	Control SD	Control Total	Weight	Std. Mean Difference IV, Random, 95% CI
**21.1.1 Diabetes**								
Shera 2023 versus Spencer mobilization	4.09	0.933	12	2.75	0.972	12	4.4%	1.36 [0.45, 2.26]
**Subtotal (95% CI)**			**12**			**12**	**4.4%**	**1.36 [0.45, 2.26]**
Heterogeneity: Not applicable								
Test for overall effect: Z = 2.95 (P = 0.003)								
**21.1.2 Diabetes allowed**								
Mehta 2018 versus passive stretching	30.6	38.604	30	49	38.604	30	5.1%	-0.47 [-0.98, 0.04]
Jung 2020 versus continuous passive motion	27.2	7.54	15	28.67	8.3	15	4.8%	-0.18 [-0.90, 0.54]
Rayudu 2019 versus Muscle Energy Technique	11.93	3.574	30	11.23	3.068	30	5.1%	0.21 [-0.30, 0.71]
Azam 2024 verus Maitland mobilization	4.1052	0.618	19	3.95	0.781	20	4.9%	0.22 [-0.41, 0.85]
Dilip 2016 versus Gong's mobilization	2.47	0.454	20	2.07	0.702	20	4.9%	0.66 [0.02, 1.30]
Jeyakumar 2018 versus Maitland mobilization	1.315	1.486	35	0	1.486	35	5.1%	0.88 [0.38, 1.37]
Zaghloul 2022 versus scapular mobilization	5.72	1.107	18	4.73	1.036	18	4.8%	0.90 [0.21, 1.59]
Doner 2013 versus stretching	5.15	1.743	20	3.36	1.165	20	4.8%	1.18 [0.51, 1.86]
Rathod 2019 verus Kaltenborn mobilization	4	0.858	20	3	0.795	20	4.8%	1.19 [0.51, 1.86]
Khan 2023 versus Sleeper stretch	4.87	0.952	22	3.091	1.148	22	4.8%	1.66 [0.96, 2.35]
Patil 2010 versus Kaltenborn mobilization	4.7	0.93	30	3.2	0.81	30	5.0%	1.70 [1.10, 2.29]
Boruah 2015 versus scapular mobilization	20.48	5.221	25	10.64	5.074	25	4.8%	1.88 [1.21, 2.56]
Razzaq 2022 versus Muscle Energy Technique	5.41	0.49	32	2.07	0.39	29	3.4%	7.40 [5.96, 8.85]
**Subtotal (95% CI)**			**316**			**314**	**62.2%**	**1.20 [0.58, 1.82]**
Heterogeneity: Tau² = 1.17; Chi² = 146.21, df = 12 (P < 0.00001); I² = 92%								
Test for overall effect: Z = 3.79 (P = 0.0002)								
**21.1.3 No diabetes**								
Fernandes 2020 versus Kaltenborn mobilization	22.07	56.649	28	31.56	81.008	28	5.1%	-0.13 [-0.66, 0.39]
Pankaj 2013 versus Gong’s mobilization	2.03	0.641	15	1.97	0.686	15	4.8%	0.09 [-0.63, 0.80]
Khalil 2022 versus Muscle Energy Technique	4.08	0.5	39	4	0.547	39	5.2%	0.15 [-0.29, 0.60]
Khyathi 2015 versus Spencer mobilization	3.34	3.385	20	2.72	0.964	20	4.9%	0.24 [-0.38, 0.87]
Patel 2022 versus Muscle Energy Technique	2.66	1.11	21	2.09	0.88	21	4.9%	0.56 [-0.06, 1.18]
Ranjana 2016 versus Maitland mobilization	4.32	0.592	15	3.76	0.369	15	4.7%	1.10 [0.33, 1.88]
Nithya 2021 versus Muscle Energy Technique	4.07	0.676	15	1.94	0.465	15	3.9%	3.57 [2.37, 4.78]
**Subtotal (95% CI)**			**153**			**153**	**33.3%**	**0.66 [0.06, 1.26]**
Heterogeneity: Tau² = 0.53; Chi² = 36.14, df = 6 (P < 0.00001); I² = 83%								
Test for overall effect: Z = 2.15 (P = 0.03)								
**Total (95% CI)**			**481**			**479**	**100.0%**	**1.03 [0.59, 1.46]**
Heterogeneity: Tau² = 0.92; Chi² = 194.07, df = 20 (P < 0.00001); I² = 90%								
Test for overall effect: Z = 4.58 (P < 0.00001)								
Test for subgroup differences: Chi² = 2.25, df = 2 (P = 0.32), I² = 11.1%								

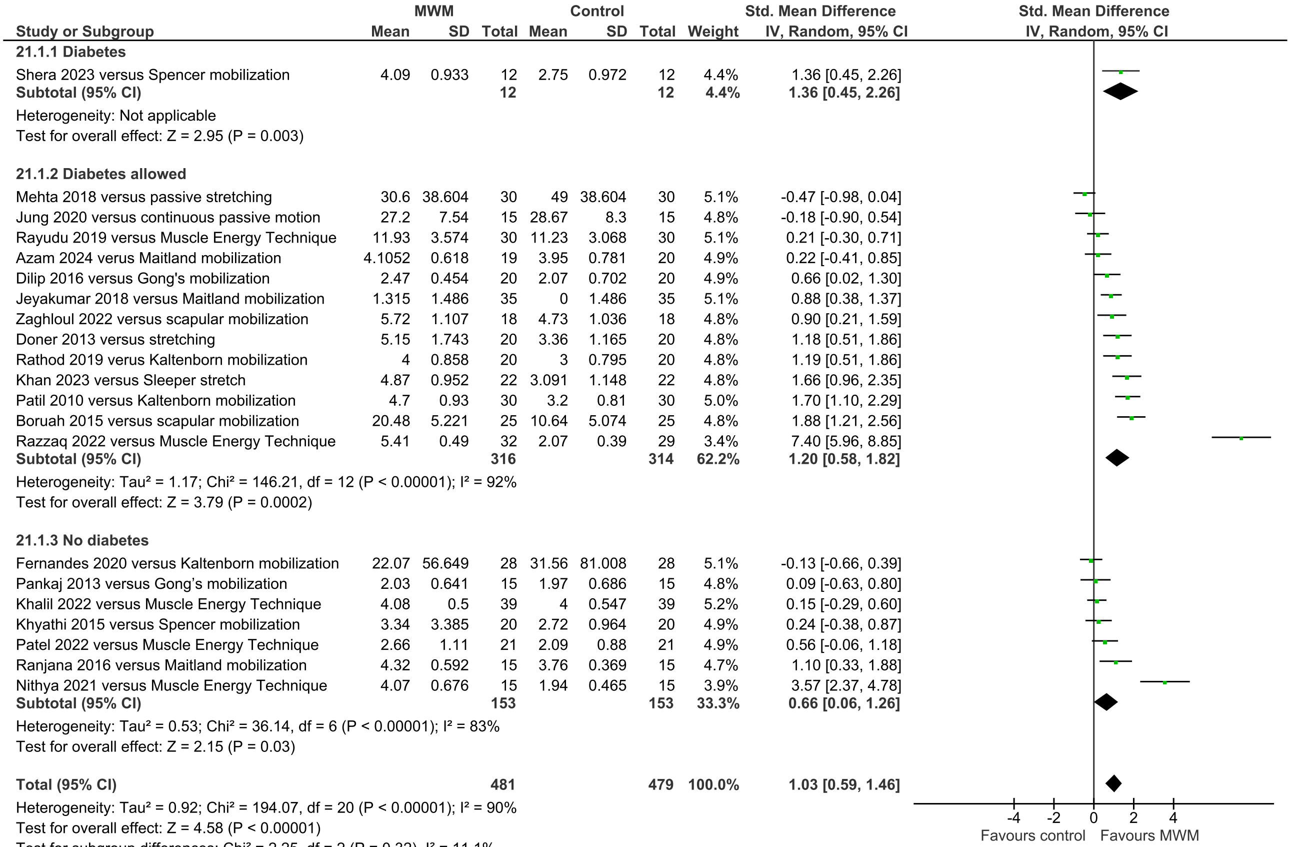

Figure S18 Forest plot of AC pain results immediately post-therapy – MWM versus other types of mobilization – subgrouped by diabetes status.
AC = adhesive capsulitis; MWM = Mobilization With Movement.

Study or Subgroup	MWM Mean	MWM SD	MWM Total	Control Mean	Control SD	Control Total	Weight	Std. Mean Difference IV, Random, 95% CI
**22.1.2 Diabetes**								
Mehta 2018 versus passive stretching	29.2	76.375	30	42.9	76.375	30	14.9%	-0.18 [-0.68, 0.33]
Rayudu 2019 versus Muscle Energy Technique	16.84	5.584	30	14.07	5.645	30	14.9%	0.49 [-0.03, 1.00]
Doner 2013 versus stretching	57.23	21.732	20	34.68	24.539	20	14.1%	0.95 [0.30, 1.61]
**Subtotal (95% CI)**			**80**			**80**	**43.9%**	**0.40 [-0.23, 1.02]**
Heterogeneity: Tau² = 0.23; Chi² = 7.65, df = 2 (P = 0.02); I² = 74%								
Test for overall effect: Z = 1.24 (P = 0.22)								
**22.1.3 No diabetes**								
Fernandes 2020 versus Kaltenborn mobilization	20.44	52.465	28	29.86	76.644	28	14.9%	-0.14 [-0.67, 0.38]
Yang 2007 versus Maitland end-range mobilization	9	4.061	13	5.2	3.774	14	13.2%	0.94 [0.14, 1.74]
Khalil 2022 versus Muscle Energy Technique	68.94	15.665	39	47.93	7.049	39	14.9%	1.71 [1.19, 2.24]
Khyathi 2015 versus Spencer mobilization	23.98	3.012	20	15.87	4.198	20	13.2%	2.18 [1.38, 2.97]
**Subtotal (95% CI)**			**100**			**101**	**56.1%**	**1.16 [0.09, 2.23]**
Heterogeneity: Tau² = 1.08; Chi² = 33.68, df = 3 (P < 0.00001); I² = 91%								
Test for overall effect: Z = 2.12 (P = 0.03)								
**Total (95% CI)**			**180**			**181**	**100.0%**	**0.82 [0.17, 1.48]**
Heterogeneity: Tau² = 0.68; Chi² = 50.51, df = 6 (P < 0.00001); I² = 88%								
Test for overall effect: Z = 2.48 (P = 0.01)								
Test for subgroup differences: Chi² = 1.44, df = 1 (P = 0.23), I² = 30.7%								

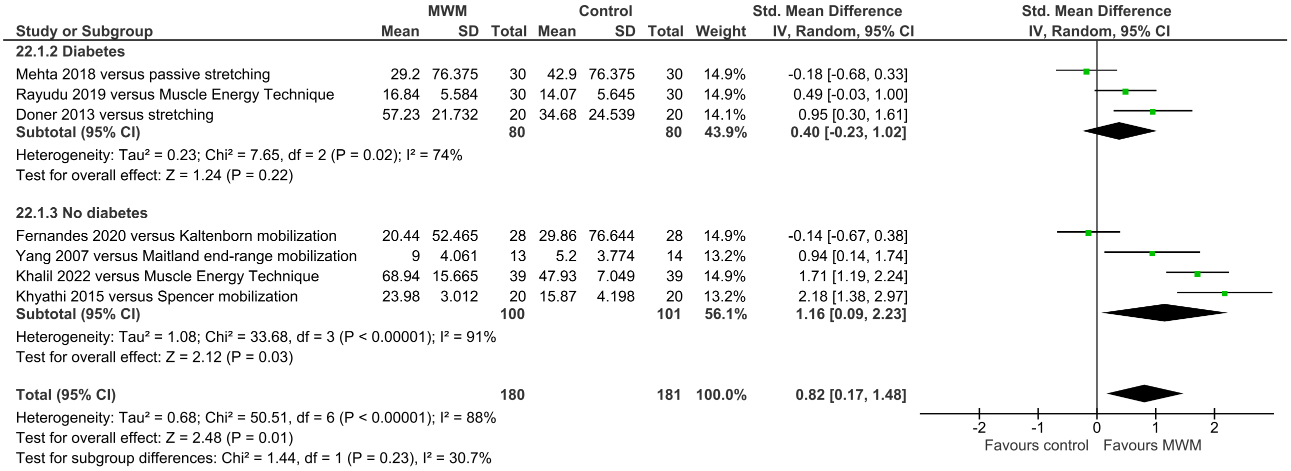

Figure S19 Forest plot of AC disability results immediately post-therapy – MWM versus other types of mobilization – subgrouped by diabetes status.
AC = adhesive capsulitis; MWM = Mobilization With Movement.

Study or Subgroup	MWM Mean	MWM SD	MWM Total	Control Mean	Control SD	Control Total	Weight	Std. Mean Difference IV, Random, 95% CI
**23.1.1 Diabetes**								
Youssef 2015 versus Maitland mobilization	59.29	14.637	15	48.29	14.637	15	4.9%	0.73 [-0.01, 1.47]
Khyathi 2015 versus Spencer mobilization	19.8	3.205	20	10.95	2.946	20	4.6%	2.82 [1.92, 3.72]
**Subtotal (95% CI)**			**35**			**35**	**9.4%**	**1.76 [-0.29, 3.80]**
Heterogeneity: Tau² = 2.00; Chi² = 12.31, df = 1 (P = 0.0005); I² = 92%								
Test for overall effect: Z = 1.69 (P = 0.09)								
**23.1.2 Diabetes allowed**								
Patil 2010 versus Kaltenborn mobilization	26.3	7.28	30	34.5	5.02	30	5.2%	-1.29 [-1.85, -0.73]
Zaghloul 2022 versus scapular mobilization	23.36	5.005	18	27.86	5.205	18	5.0%	-0.86 [-1.55, -0.18]
Mehta 2018 versus passive stretching	3.8	15.023	30	11.6	15.023	30	5.3%	-0.51 [-1.03, 0.00]
Rathod 2019 verus Kaltenborn mobilization	20.3	4.669	20	19.5	6.621	20	5.1%	0.14 [-0.48, 0.76]
Rayudu 2019 versus Muscle Energy Technique	14.66	7.267	30	13.5	6.719	30	5.3%	0.16 [-0.34, 0.67]
Arshad 2015 versus Maitland mobilization	43.84	7.185	50	39.62	9.403	50	5.4%	0.50 [0.10, 0.90]
Doner 2013 versus stretching	30.5	19.084	20	20.25	19.867	20	5.1%	0.52 [-0.12, 1.15]
Minerva 2016 versus Maitland mobilization	16.67	7.161	30	11.5	7.161	30	5.2%	0.71 [0.19, 1.24]
Jeyakumar 2018 versus Maitland mobilization	9.571	10.71	35	0	10.71	35	5.3%	0.88 [0.39, 1.38]
Boruah 2015 versus scapular mobilization	21.2	6.32	25	11.44	5.391	25	5.0%	1.64 [0.99, 2.28]
Azam 2024 verus Maitland mobilization	33.8421	2.637	19	28.2	3.285	20	4.8%	1.85 [1.09, 2.61]
Khan 2023 versus Sleeper stretch	30.46	5.388	22	18.63	6.292	22	4.9%	1.98 [1.25, 2.72]
**Subtotal (95% CI)**			**329**			**330**	**61.6%**	**0.46 [-0.06, 0.99]**
Heterogeneity: Tau² = 0.77; Chi² = 112.95, df = 11 (P < 0.00001); I² = 90%								
Test for overall effect: Z = 1.72 (P = 0.09)								
**23.1.3 No diabetes**								
Yang 2007 versus Maitland end-range mobilization	34.2	14.3	13	36.4	24.3	14	4.8%	-0.11 [-0.86, 0.65]
Fernandes 2020 versus Kaltenborn mobilization	14.21	13.109	28	12.86	19.515	28	5.2%	0.08 [-0.44, 0.60]
Khalil 2022 versus Muscle Energy Technique	14.15	7.841	39	8.54	8.76	39	5.3%	0.67 [0.21, 1.12]
Ranjana 2016 versus Maitland mobilization	34.87	10.946	15	25.74	14.246	15	4.9%	0.70 [-0.04, 1.44]
Patel 2022 versus Muscle Energy Technique	34.9	7.68	21	21.76	3.63	21	4.8%	2.15 [1.37, 2.92]
Nithya 2021 versus Muscle Energy Technique	40.53	3.348	15	28.67	2.776	15	3.9%	3.75 [2.51, 5.00]
**Subtotal (95% CI)**			**131**			**132**	**29.0%**	**1.10 [0.26, 1.95]**
Heterogeneity: Tau² = 0.96; Chi² = 46.20, df = 5 (P < 0.00001); I² = 89%								
Test for overall effect: Z = 2.56 (P = 0.01)								
**Total (95% CI)**			**495**			**497**	**100.0%**	**0.77 [0.33, 1.20]**
Heterogeneity: Tau² = 0.85; Chi² = 189.93, df = 19 (P < 0.00001); I² = 90%								
Test for overall effect: Z = 3.48 (P = 0.0005)								
Test for subgroup differences: Chi² = 2.70, df = 2 (P = 0.26), I² = 25.8%								

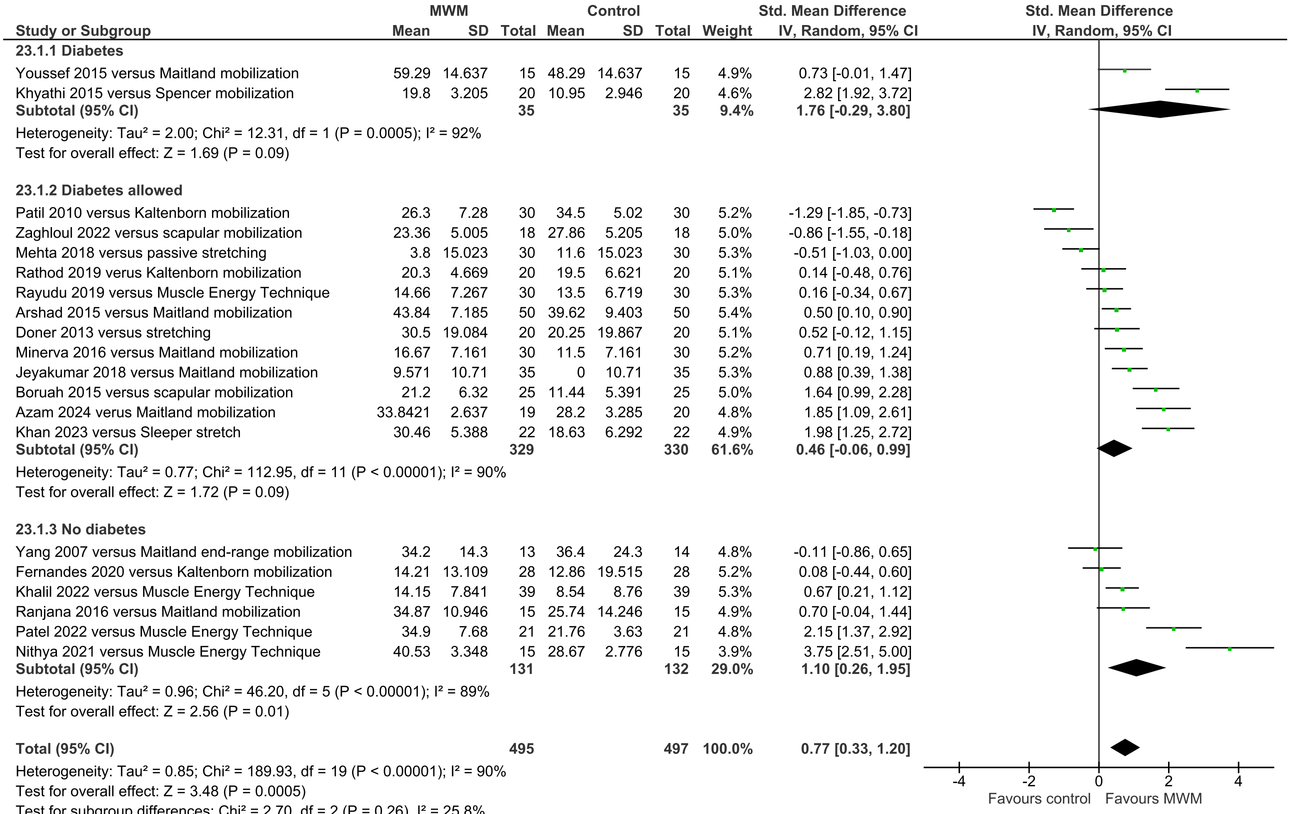

Figure S20 Forest plot of AC external rotation ROM results immediately post-therapy – MWM versus other types of mobilization – subgrouped by diabetes status.
AC = adhesive capsulitis; MWM = Mobilization With Movement; ROM = range of motion.

Study or Subgroup	MWM Mean	MWM SD	MWM Total	Control Mean	Control SD	Control Total	Weight	Std. Mean Difference IV, Random, 95% CI
**24.1.1 Diabetes**								
Youssef 2015 versus Maitland mobilization	91.54	33.214	15	66.58	33.214	15	7.1%	0.73 [-0.01, 1.47]
**Subtotal (95% CI)**			**15**			**15**	**7.1%**	**0.73 [-0.01, 1.47]**
Heterogeneity: Not applicable								
Test for overall effect: Z = 1.93 (P = 0.05)								
**24.1.2 Diabetes allowed**								
Zaghloul 2022 versus scapular mobilization	15.07	5.6	18	23.93	5.39	18	7.1%	-1.58 [-2.33, -0.82]
Patil 2010 versus Kaltenborn mobilization	44	8.84	30	52.3	3.97	30	7.4%	-1.20 [-1.75, -0.64]
Mehta 2018 versus passive stretching	7.1	15.601	30	15.2	15.601	30	7.4%	-0.51 [-1.03, 0.00]
Arshad 2015 versus Maitland mobilization	105.68	14.089	50	102.12	15.425	50	7.5%	0.24 [-0.15, 0.63]
Doner 2013 versus stretching	38	14.547	20	31.25	15.996	20	7.3%	0.43 [-0.20, 1.06]
Khan 2023 versus Sleeper stretch	62.27	8.29	22	56.36	14.37	22	7.3%	0.49 [-0.11, 1.10]
Rathod 2019 verus Kaltenborn mobilization	36.35	9.778	20	27.9	8.404	20	7.2%	0.91 [0.25, 1.56]
Jung 2020 versus continuous passive motion	37.67	4.95	15	29.33	3.72	15	6.9%	1.85 [0.98, 2.73]
Boruah 2015 versus scapular mobilization	45.96	8.217	25	26.08	7.198	25	7.1%	2.53 [1.78, 3.29]
Razzaq 2022 versus Muscle Energy Technique	90.75	8.99	32	31.8	7.29	29	5.9%	7.07 [5.68, 8.47]
**Subtotal (95% CI)**			**262**			**259**	**71.0%**	**0.93 [-0.02, 1.87]**
Heterogeneity: Tau² = 2.19; Chi² = 201.65, df = 9 (P < 0.00001); I² = 96%								
Test for overall effect: Z = 1.91 (P = 0.06)								
**24.1.3 No diabetes**								
Fernandes 2020 versus Kaltenborn mobilization	21.81	12.26	28	28.68	7.23	28	7.4%	-0.67 [-1.21, -0.13]
Khalil 2022 versus Muscle Energy Technique	28.72	17.615	39	15.82	20.762	39	7.5%	0.66 [0.21, 1.12]
Patel 2022 versus Muscle Energy Technique	67.76	11.92	21	46.66	8.87	21	7.1%	1.97 [1.22, 2.72]
**Subtotal (95% CI)**			**88**			**88**	**21.9%**	**0.63 [-0.71, 1.97]**
Heterogeneity: Tau² = 1.31; Chi² = 33.28, df = 2 (P < 0.00001); I² = 94%								
Test for overall effect: Z = 0.93 (P = 0.35)								
**Total (95% CI)**			**365**			**362**	**100.0%**	**0.83 [0.13, 1.53]**
Heterogeneity: Tau² = 1.67; Chi² = 236.45, df = 13 (P < 0.00001); I² = 95%								
Test for overall effect: Z = 2.31 (P = 0.02)								
Test for subgroup differences: Chi² = 0.15, df = 2 (P = 0.93), I² = 0%								

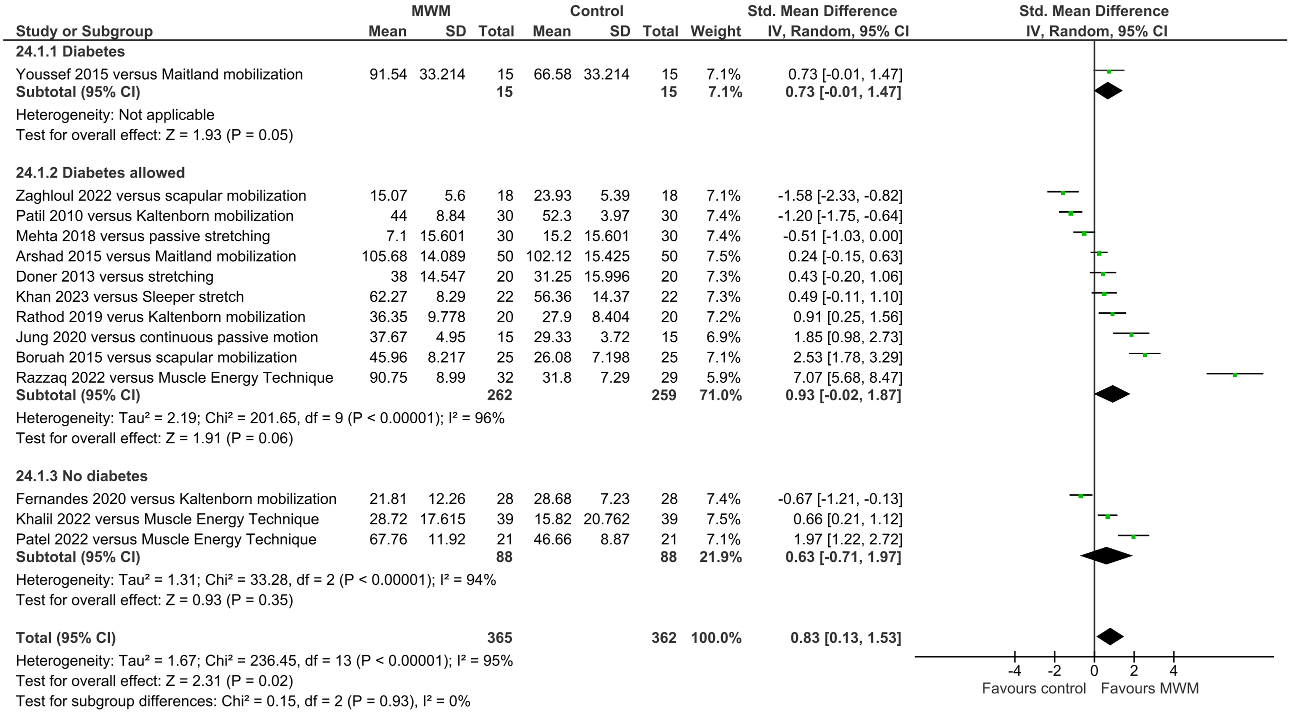

Figure S21 Forest plot of AC flexion ROM results immediately post-therapy – MWM versus other types of mobilization – subgrouped by diabetes status.
AC = adhesive capsulitis; MWM = Mobilization With Movement; ROM = range of motion.

Study or Subgroup	MWM Mean	MWM SD	MWM Total	Control Mean	Control SD	Control Total	Weight	Std. Mean Difference IV, Random, 95% CI
**25.1.1 Duration of pain stated in report**								
Mehta 2018 versus passive stretching	30.6	38.604	30	49	38.604	30	5.1%	-0.47 [-0.98, 0.04]
Jung 2020 versus continuous passive motion	27.2	7.54	15	28.67	8.3	15	4.8%	-0.18 [-0.90, 0.54]
Fernandes 2020 versus Kaltenborn mobilization	22.07	56.649	28	31.56	81.008	28	5.1%	-0.13 [-0.66, 0.39]
Pankaj 2013 versus Gong's mobilization	2.03	0.641	15	1.97	0.686	15	4.8%	0.09 [-0.63, 0.80]
Azam 2024 verus Maitland mobilization	4.1052	0.618	19	3.95	0.781	20	4.9%	0.22 [-0.41, 0.85]
Khyathi 2015 versus Spencer mobilization	3.34	3.385	20	2.72	0.964	20	4.9%	0.24 [-0.38, 0.87]
Patel 2022 versus Muscle Energy Technique	2.66	1.11	21	2.09	0.88	21	4.9%	0.56 [-0.06, 1.18]
Dilip 2016 versus Gong's mobilization	2.47	0.454	20	2.07	0.702	20	4.9%	0.66 [0.02, 1.30]
Jeyakumar 2018 versus Maitland mobilization	1.315	1.486	35	0	1.486	35	5.1%	0.88 [0.38, 1.37]
Ranjana 2016 versus Maitland mobilization	4.32	0.592	15	3.76	0.369	15	4.7%	1.10 [0.33, 1.88]
Doner 2013 versus stretching	5.15	1.743	20	3.36	1.165	20	4.8%	1.18 [0.51, 1.86]
Rathod 2019 verus Kaltenborn mobilization	4	0.858	20	3	0.795	20	4.8%	1.19 [0.51, 1.86]
Shera 2023 versus Spencer mobilization	4.09	0.933	12	2.75	0.972	12	4.4%	1.36 [0.45, 2.26]
Patil 2010 versus Kaltenborn mobilization	4.7	0.93	30	3.2	0.81	30	5.0%	1.70 [1.10, 2.29]
Boruah 2015 versus scapular mobilization	20.48	5.221	25	10.64	5.074	25	4.8%	1.88 [1.21, 2.56]
Nithya 2021 versus Muscle Energy Technique	4.07	0.676	15	1.94	0.465	15	3.9%	3.57 [2.37, 4.78]
Razzaq 2022 versus Muscle Energy Technique	5.41	0.49	32	2.07	0.39	29	3.4%	7.40 [5.96, 8.85]
**Subtotal (95% CI)**			**372**			**370**	**80.2%**	**1.12 [0.58, 1.67]**
Heterogeneity: Tau² = 1.15; Chi² = 176.71, df = 16 (P < 0.00001); I² = 91%								
Test for overall effect: Z = 4.07 (P < 0.0001)								
**25.1.2 Duration of pain clarified via mail**								
Khalil 2022 versus Muscle Energy Technique	4.08	0.5	39	4	0.547	39	5.2%	0.15 [-0.29, 0.60]
Rayudu 2019 versus Muscle Energy Technique	11.93	3.574	30	11.23	3.068	30	5.1%	0.21 [-0.30, 0.71]
Zaghloul 2022 versus scapular mobilization	5.72	1.107	18	4.73	1.036	18	4.8%	0.90 [0.21, 1.59]
Khan 2023 versus Sleeper stretch	4.87	0.952	22	3.091	1.148	22	4.8%	1.66 [0.96, 2.35]
**Subtotal (95% CI)**			**109**			**109**	**19.8%**	**0.69 [0.04, 1.34]**
Heterogeneity: Tau² = 0.35; Chi² = 15.61, df = 3 (P = 0.001); I² = 81%								
Test for overall effect: Z = 2.08 (P = 0.04)								
**Total (95% CI)**			**481**			**479**	**100.0%**	**1.03 [0.59, 1.46]**
Heterogeneity: Tau² = 0.92; Chi² = 194.07, df = 20 (P < 0.00001); I² = 90%								
Test for overall effect: Z = 4.58 (P < 0.00001)								
Test for subgroup differences: Chi² = 1.02, df = 1 (P = 0.31), I² = 1.9%								

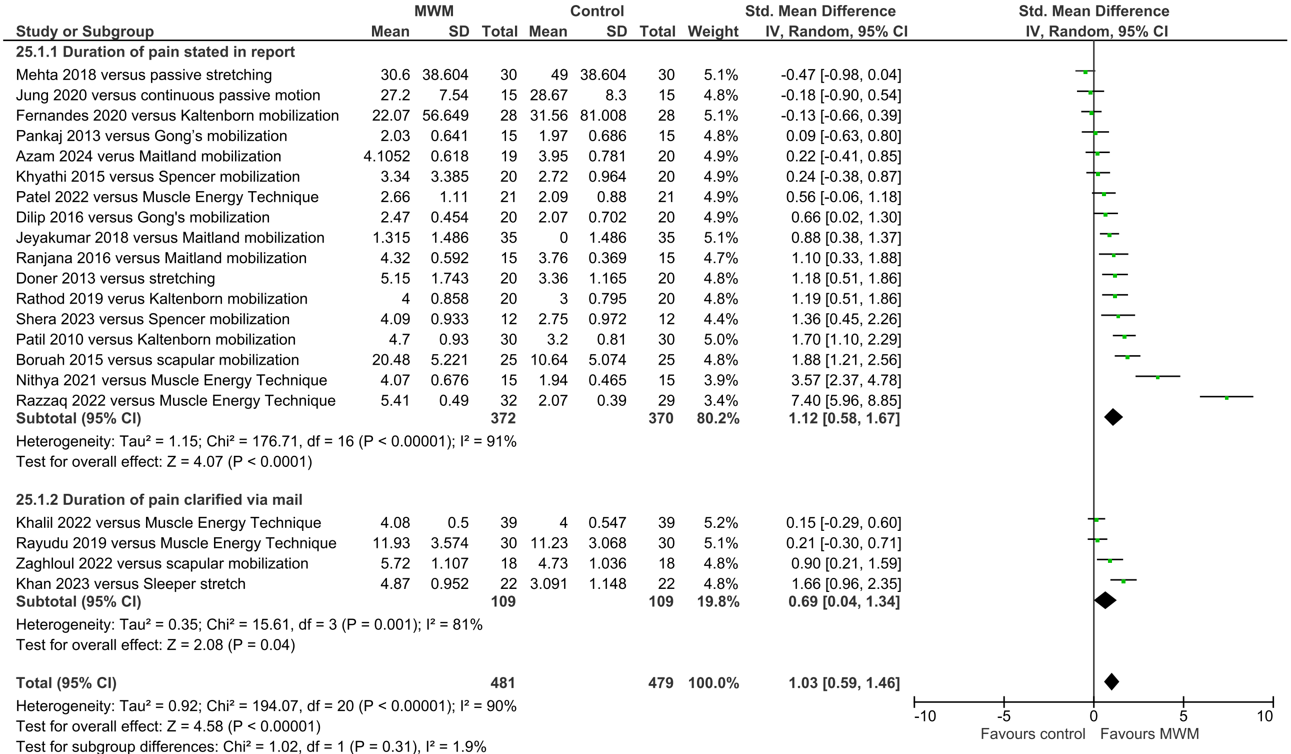

Figure S22 Forest plot of difference in immediate AC pain results between trials with and without duration of pain specified in manuscripts (post-hoc analysis).

References

1. Abu El Kasem ST, Alaa FAA, Abd El-Raoof NA, Abd-Elazeim AS: Efficacy of Mulligan thoracic sustained natural apophyseal glides on sub-acromial pain in patients with sub-acromial impingement syndrome: a single-blinded randomized controlled trial. *The Journal of manual & manipulative therapy* 2024:1-10.
2. Ajis TT, Kumar NS, Kumaresan A, Pavithra, Anitha, Kumaran M, Alagesan J: A Study to Compare the Effect of Dry Needling Technique to Mobilization on Pain Reduction in PA Shoulder. *Indian Journal of Physiotherapy & Occupational Therapy* 2024, 18:902-907.
3. Anwar M, Mughal MW, Izhar N, Rasheed M: Effectiveness of Maitland Mobilization Technique in Comparison with Mulligan Mobilization Technique in Management of Frozen Shoulder. *Pakistan Journal of Medical and Health Sciences* 2023.
4. Azin Z, Kamali F, Salehi Dehno N, Abolahrari-Shirazi S: Comparison of Manual Therapy Technique to Therapeutic Exercise in the Treatment of Patients With Subacromial Impingement Syndrome: A Randomized Clinical Trial. *J Manipulative Physiol Ther* 2023, 46(2):98-108.
5. Çelik EB, Tuncer A: Comparing the Efficacy of Manual Therapy and Exercise to Synchronized Telerehabilitation with Self-Manual Therapy and Exercise in Treating Subacromial Pain Syndrome: A Randomized Controlled Trial. *Healthcare (Basel)* 2024, 12(11).
6. Choi J-M, Cho E-Y, Lee B-H: Effects of Dynamic Stretching Combined with Manual Therapy on Pain, ROM, Function, and Quality of Life of Adhesive Capsulitis. *Healthcare (2227-9032)* 2024, 12(1):45.
7. Dabholkar A, Dabholkar T, Yardi S: Efficacy of Scapular Movement with Mobilization in Patients with Shoulder Impingement. *Indian Journal of Physiotherapy & Occupational Therapy* 2013, 7(3):20-24.
8. Eliason A, Werner S, Engström B, Harringe M: Home training with or without joint mobilization compared to no treatment: a randomized controlled trial. *Journal of physical therapy science* 2022, 34(2):153-160.
9. Goyal M, Bhattacharjee S, Goyal K: Combined Effect of End Range

Mobilization (ERM) and Mobilization with Movement (MWM) Techniques on Range of Motion and Disability in Frozen Shoulder Patients: A Randomized Clinical Trial. *Journal of Exercise Science and Physiotherapy* 2013, 9:74.
10. Gumucio JC, Traro FP, Guzman-Venegas R: Does the Axial Rotation of the Clavicle Posed by the Mulligan Maneuver Produce a Pressure Reduction in the Subacromial Space? An exploratory ex vivo study. *International Journal of Morphology* 2022, 40(5):1165-1168.
11. Gutiérrez-Espinoza H, Pinto-Concha S, Sepúlveda-Osses O, Araya-Quintanilla F: Effectiveness of scapular mobilization in people with subacromial impingement syndrome: A randomized controlled trial. *Ann Phys Rehabil Med* 2023, 66(5):101744.
12. Haider R, Ahmad A, Saum-re-Zahra., Hanif MK: To Compare Effects of Maitland and Mulligan's Mobilization Techniques in the Treatment of Frozen Shoulder. *Annals of King Edward Medical University, Lahore* 2014, 20:257-264.
13. Hasbiah H, Awal M, Halimah A, Sarman A: Different Influence of End Range Mobilization and Mobilization With Movement on Improving Motional Scope of Non Specific Frozen Shoulder. *International Journal of Sciences: Basic and Applied Research (IJSBAR)* 2018, 37(3):136-149.
14. Haveela B, Dowle P, Chandrasekhar P: Effectiveness of Mulligan’s Technique and Spencer’s Technique in Adjunct to Conventional Therapy in Frozen Shoulder: A Randomised Controlled Trial. *International Journal for Advance Research and Development* 2018, 3(1):253-260.
15. Hussein ZA: Efficacy of mobilization techniques and range of motion in patients with adhesive capsulitis of the shoulder pain. *International Journal of Research in Pharmaceutical Sciences* 2019, 10(1):313-317.
16. Jacob L, Lasbleiz S, Sanchez K, Morchoisne O, Lefèvre-Colau MM, Nguyen C, Rannou F, Feydy A, Portal JJ, Schnitzler A *et al*: Arthro-distension with early and intensive mobilization for shoulder adhesive capsulitis: A randomized controlled trial. *Ann Phys Rehabil Med* 2024, 67(6):101852.
17. Jain J, Khan J, Pal R, Jha SK: A Comparative Study Between Joint Mobilisation and Muscle Energy Technique in Patients with Adhesive Capsulitis. *International Journal of Pharmaceutical and Clinical Research* 2023, 15(6):2037-2042.
18. Jie H, Lingfeng X, Xiaoling H, Xiaohua H: Effects of mulligan's mobilization with movement combined with stretching therapy in the management of frozen shoulder. *Physiotherapy (United Kingdom)* 2015, 101(SUPPL. 1):eS683-eS684.
19. Karakus S, Gelecek N, Yesilyaprak SS: Effects of proprioceptive neuromuscular facilitation and mulligan concepts on the pain, functional level and quality of life on subacromial impingement syndrome. *Orthopaedic journal of sports medicine* 2014, 2(11 Supplement 3).
20. Karasuno H, Hamada J, Yano Y, Tsutsui H, Hagiwara Y, Endo K, Saito T: Adduction Manipulation of the Glenohumeral Joint versus Physiotherapy for Atraumatic Rotator Cuff Tears: A Randomized Controlled Trial. *Journal of clinical medicine* 2023, 12(12).
21. Kazmi SAM, Devi J, Yamin F, Kumar S: Comparative study on the efficacy of Maitland technique (Grade IV) and mulligan technique, in the treatment of frozen shoulder. *Pakistan Journal of rehabilitation* 2013, 2(1):10-14.
22. Khandelwal P, Khan J, Renuka PT, Khan U: THE IMPACT OF SCAPULAR MOBILIZATION ON THE EFFECTIVENESS OF ROM EXERCISES IN PATIENTS WITH ADHESIVE CAPSULITIS. *International Journal of Current Pharmaceutical Research* 2023, 15(4):80-84.
23. Lin P, Yang M, Huang D, Lin H, Wang J, Zhong C, Guan L: Effect of proprioceptive neuromuscular facilitation technique on the treatment of frozen shoulder: a pilot randomized controlled trial. *BMC musculoskeletal disorders* 2022, 23(1):367.
24. Madhumita R, Ramana K, Anitha A, Kamalakannan M: Effect of Mulligan Techniques on Pain Reduction and Improving Functional Activity of Shoulder Among Periarthritis Shoulder Individuals. *Indian Journal of*

Physiotherapy & Occupational Therapy 2024, 18:50-56.
25. Premkumar M, Sureshkumar AR, Madhuripu, Kavitha S: Effect of mulligan mobilization with ultrasound VS conventional rehabilitation for grade II supraspinatus tendinitis – Quasi experimental study. *Journal of Orthopaedic Reports* 2024, 3(3):100321.
26. Menek B, Algun C, Tarakci D: Effectiveness of mulligan mobilization on range of motion and function in individuals with subacromial impingement syndrome. *Osteoporosis International* 2018, 29(1 Supplement 1):S461-S462.
27. Menek B, Tarakci D, Algun ZC: The effect of Mulligan mobilization on pain and life quality of patients with Rotator cuff syndrome: A randomized controlled trial. *Journal of back and musculoskeletal rehabilitation* 2019, 32(1):171-178.
28. Michener LA, McClure PW, Tate AR, Bailey LB, Seitz AL, Straub RK, Thigpen CA: Adding Manual Therapy to an Exercise Program Improves Long-Term Patient Outcomes Over Exercise Alone in Patients With Subacromial Shoulder Pain: A Randomized Clinical Trial. *JOSPT Open* 2024.
29. Moradi M, Shadmehr A, Fischer T, Attarbashi Moghaddam B, Ebrahimzade MH, Jalaei S: Comparison of the efficacy of manual treatment according to fascial distortion model versus joint mobilization in patients with shoulder impingement: A randomized clinical trial. *J Bodyw Mov Ther* 2023, 36:410-416.
30. Raghav S, Singh A: Comparison of Effectiveness of Mulligan 'MWM' Technique Versus Kaltenborn Mobilization Technique on Pain and End Range of Motion in Patients with Adhesive Capsulitis of Shoulder Joint : A Randomized Controlled Trial. *Journal of Exercise Science and Physiotherapy* 2019, 15.
31. Rana AALI, Fatima S, Sajjad SALI, Niaz M, Hayat MK, Ahmad I: Effectiveness of maitland vs. mulligan mobilization techniques in adhesive capsulitis of shoulder joint. *Pakistan Journal of Medical and Health Sciences* 2021, 15(9):2561-2564.
32. Razaq HB, Basharat A, Sheikh SA, Zehra RE, Mufti HA, Faraz K, Waqar Z: Effects of combining both mobilization with movement and spencer's technique in diabetic adhesive capsulitis patients. *International journal of health sciences* 2023.
33. Lirio Romero C, Torres Lacomba M, Castilla Montoro Y, Prieto Merino D, Pacheco da Costa S, Velasco Marchante MJ, Bodes Pardo G: Mobilization With Movement for Shoulder Dysfunction in Older Adults: A Pilot Trial. *Journal of chiropractic medicine* 2015, 14(4):249-258.
34. Saha T: Effectiveness of movement with mobilization to improve range of motion among adhesive capsulitis patients attended at CRP: Bangladesh Health Professions Institute, Bangladesh; 2015.
35. Satpute KH, Bhandari P, Hall T: Efficacy of Hand Behind Back Mobilization With Movement for Acute Shoulder Pain and Movement Impairment: A Randomized Controlled Trial. *J Manipulative Physiol Ther* 2015, 38(5):324-334.
36. Sharma D, Prasad V, Rastogi D, Rastogi M, Srivastava A: Comparing the efficacy of movement with mobilization in respect to graded mobilization in adhesive capsulitis of shoulder. *International Journal of Health Sciences* 2022, 6(S2): 7915-7922.
37. Si JF, Liu F, Zhang YS, Song L, Liu WM: Mulligan dynamic joint mobilization operation combined with acupuncture and tuina in the treatment of periarthritis of shoulder of 35 cases. *Chinese medicine modern distance education of china [zhong guo zhong yi yao xian dai yuan chen jiao yu]* 2016, 14(23):105-107.
38. Conte da Silva A, Aily JB, Mattiello SM: Ischemic compression associated with joint mobilization does not promote additional clinical effects in individuals with rotator cuff related shoulder pain: A randomized clinical trial. *J Bodyw Mov Ther* 2023, 36:335-342.
39. Srivastava N, Joshi S: Comparision between the Effectiveness of Mobilization with Movement and End Range Mobilization along with Conventional Therapy for Management of Frozen Shoulder. *Indian Journal of Physiotherapy & Occupational Therapy* 2017, 11(4):176-179.
40. Srivastava S, Eapen C, Mittal H: Comparison of mobilisation with movement and cryotherapy in shoulder impingement syndrome-A randomised clinical trial. *Journal of Clinical and*

Diagnostic Research 2018, 12(10):YC01-YC05.
41. Subashini A: To Compare Effects of Mulligan 'S and Maitland Moblization Techniques in the Treatment of Periarthritis Shoulder. *Bulletin of Pure and Applied Sciences-Zoology* 2024:836-840.
42. Subhash R, Makhija M: Effectiveness of mobilization with movement in weight bearing position on pain, shoulder range of motion and function in patients with shoulder dysfunction. *Indian Journal of Public Health Research and Development* 2020, 11(6):912-916.
43. Taghipour M, Ramezani M, Oliaei F, Bahrami M: Comparison of Taping and Mobilization on Pain, Range of Motion and Shoulder Disability in Sub Acromial Impingement Syndrome in Dialysis Patients. *Iranian Rehabilitation Journal* 2024, 22(1):1-10.
44. Tauqeer S, Arooj A, Shakeel H: Effects of manual therapy in addition to stretching and strengthening exercises to improve scapular range of motion, functional capacity and pain in patients with shoulder impingement syndrome: a randomized controlled trial. *BMC musculoskeletal disorders* 2024, 25(1):192.
45. Teys P, Bisset L, Vicenzino B: The initial effects of a Mulligan's mobilization with movement technique on range of movement and pressure pain threshold in pain-limited shoulders. *Manual therapy* 2008, 13(1):37-42.
46. Wang Y, Wang C, Chen H, Ye X: [Shoulder joint pain of rotator cuff injury treated with electroacupuncture and Mulligan's mobilization: a randomized controlled trial]. *Zhongguo zhen jiu = Chinese acupuncture & moxibustion* 2018, 38(1):17-21.
47. Yeonki C: Effects of mobilization with movement combined with exercise (EMWM) on ADH, ROM and functional performance in patients with impingement syndrome of the shoulder. *Journal of The Korean Society of Integrative Medicine* 2019, 7(2):153-163.
